# Supplementary material for: Occupational justice and social inclusion among people living with HIV and people with mental illness: a scoping review
Source: BMJ Open. 2020 Aug 11;10(8):e036916. doi: 10.1136/bmjopen-2020-036916 (PMC7418773; doi:10.1136/bmjopen-2020-036916)
Supplement: Supplementary data [file bmjopen-2020-036916supp001.pdf]

Appendix 1. PubMed Search Strategy

PubMed

Set 1

1. Social Justice [MeSH]

2. Social Isolation [MeSH]

3. Social Marginalization [MeSH]

4. Social Participation [MeSH]

5. Rehabilitation, Vocational [MeSH]

6. Injustice OR justice OR social inclusion OR social exclusion OR social isolation OR social separation OR social barriers OR social distance OR social acceptance OR social rejection OR social participation OR deprivation OR marginalization OR alienation

7. 1 OR 2 OR 3 OR 4 OR 5 OR 6 *(represents Social inclusion set)*

Set 2

1. Social stigma [MeSH]

2. Prejudice [MeSH]

3. Stigma OR prejudice OR stigmatise OR stigmatisation OR stigmatize OR stigmatization OR discrimination

4. 1 OR 2 OR 3 *(represents Stigma set)*

Set 3

1. Occupational justice OR Occupational injustice OR Occupational deprivation OR Occupational alienation OR Occupational marginalisation OR Occupational imbalance OR Occupational OR occupation OR occupations OR activities OR work OR employment OR unemployment OR engagement (*this set is used to narrow search to occupation as defined by OT not PubMed's definition)*

Set 4

1. Mental Disorders [MeSH] *(this heading includes substance-related disorders)*

2. Mentally Ill Persons [MeSH]

3. Mental disorders OR mental illness OR mentally ill OR Psychiatric disorder OR psychiatric illness OR psychological disorder OR Developmental Disability OR Intellectual Development Disorder OR Intellectual disability OR Mental retardation OR Mental deficiency
4. 1 OR 2 OR 3 *(represents Mental Disorders set)*

Set 5

1. HIV [MeSH]
2. HIV Infections [MeSH]
3. Acquired Immunodeficiency Syndrome (MeSH)
4. HIV OR human immune deficiency virus OR AIDS OR acquired immunodeficiency syndrome OR acquired immune deficiency syndrome OR HIV/AIDS
5. 1 OR 2 OR 3 OR 4

Now combine Sets, 1 AND 2 AND 3 AND 4 or Sets 1 AND 2 AND 3 AND 5
Limit to last 20 years

English

Date: 31/01/19
