# Supplementary material for: Occupational justice and social inclusion among people living with HIV and people with mental illness: a scoping review
Source: BMJ Open. 2020 Aug 11;10(8):e036916. doi: 10.1136/bmjopen-2020-036916 (PMC7418773; doi:10.1136/bmjopen-2020-036916)
Supplement: Author's manuscript [file bmjopen-2020-036916.draft_revisions.pdf]

BMJ Open is committed to open peer review. As part of this commitment we make the peer review history of every article we publish publicly available.

When an article is published we post the peer reviewers' comments and the authors' responses online. We also post the versions of the paper that were used during peer review. These are the versions that the peer review comments apply to.

The versions of the paper that follow are the versions that were submitted during the peer review process. They are not the versions of record or the final published versions. They should not be cited or distributed as the published version of this manuscript.

BMJ Open is an open access journal and the full, final, typeset and author-corrected version of record of the manuscript is available on our site with no access controls, subscription charges or pay-per-view fees (http://bmjopen.bmj.com).

If you have any questions on BMJ Open's open peer review process please email info.bmjopen@bmj.com

BMJ Open

Occupational justice and social inclusion amongst people living with HIV and people with mental illness: A scoping review

Journal:	*BMJ Open*
Manuscript ID	bmjopen-2020-036916
Article Type:	Original research
Date Submitted by the Author:	10-Jan-2020
Complete List of Authors:	NHUNZVI, CLEMENT; University of Zimbabwe, College of Health Sciences, Rehabilitation Department<br>Langhaug, Lisa; African Mental Health Research Initiative (AMARI) , Department of Psychiatry, College of Health Sciences. University of Zimbabwe.<br>Mavindidze, Edwin; Ingutsheni Central Hospital, Occupational Therapy<br>Harding, Richard; King's College London, Florence Nightingale Faculty of Nursing Midwifery and Palliative Care, Cicely Saunders Institute.<br>Galvaan, Roshan ; University of Cape Town, Health and Rehabilitation Sciences
Keywords:	MENTAL HEALTH, HIV & AIDS < INFECTIOUS DISEASES, REHABILITATION MEDICINE, PUBLIC HEALTH

SCHOLARONE™
Manuscripts

I, the Submitting Author has the right to grant and does grant on behalf of all authors of the Work (as defined in the below author licence), an exclusive licence and/or a non-exclusive licence for contributions from authors who are: i) UK Crown employees; ii) where BMJ has agreed a CC-BY licence shall apply, and/or iii) in accordance with the terms applicable for US Federal Government officers or employees acting as part of their official duties; on a worldwide, perpetual, irrevocable, royalty-free basis to BMJ Publishing Group Ltd ("BMJ") its licensees and where the relevant Journal is co-owned by BMJ to the co-owners of the Journal, to publish the Work in this journal and any other BMJ products and to exploit all rights, as set out in our licence.

The Submitting Author accepts and understands that any supply made under these terms is made by BMJ to the Submitting Author unless you are acting as an employee on behalf of your employer or a postgraduate student of an affiliated institution which is paying any applicable article publishing charge ("APC") for Open Access articles. Where the Submitting Author wishes to make the Work available on an Open Access basis (and intends to pay the relevant APC), the terms of reuse of such Open Access shall be governed by a Creative Commons licence – details of these licences and which Creative Commons licence will apply to this Work are set out in our licence referred to above.

Other than as permitted in any relevant BMJ Author's Self Archiving Policies, I confirm this Work has not been accepted for publication elsewhere, is not being considered for publication elsewhere and does not duplicate material already published. I confirm all authors consent to publication of this Work and authorise the granting of this licence.

For peer review only

Title: Occupational justice and social inclusion amongst people living with HIV and people with mental illness: A scoping review.

*Clement Nhunzvi[1,2,3]; Lisa Langhaug[3]; Edwin Mavindidze[4]; Richard Harding[5] & Roshan Galvaan[2]

* **Corresponding author: Clement Nhunzvi**

Department of Rehabilitation
University of Zimbabwe, College of Health Sciences
P.O. Box A178
Avondale, Harare
Zimbabwe
clemynhu@gmail.com; nhncle001@myuct.ac.za
TEL: +263 4 797800 Cell: +263 775 814 587

Affiliations

[1] Department of Rehabilitation, College of Health Sciences. University of Zimbabwe. Zimbabwe.

[2] Division of Occupational Therapy, Department of Health and Rehabilitation Sciences, Faculty of Health Sciences, University of Cape Town. South Africa.

[3] African Mental Health Research Initiative (AMARI), Department of Psychiatry, College of Health Sciences. University of Zimbabwe. Zimbabwe.

[4] Occupational Therapy Department, Ingutsheni Central Hospital, Bulawayo. Zimbabwe.

[5] Florence Nightingale Faculty of Nursing Midwifery and Palliative Care, Kings College London, Cicely Saunders Institute. UK.

Keywords: social inclusion, occupational justice, scoping review, HIV, mental illness

Word count: 3987

For peer review only

For peer review only - http://bmjopen.bmj.com/site/about/guidelines.xhtml

Abstract

Objective To explore ways in which occupational justice and social inclusion are conceptualised, defined, and operationalised in highly stigmatised and chronic conditions of mental illness and HIV.

Design This scoping review protocol followed Arksey and O`Malley`s (2005) Scoping Review Framework.

Data sources and eligibility. The following databases were searched for the period January 1997 – January 2019: Medline via PubMed, Scopus, Academic Search Premier, Cumulative Index to Nursing and Allied Health Literature (CINAHL), Africa-Wide Information, Humanities International Complete, Web of Science, PsychInfo, SocINDEX and grey literature.

Eligible articles were primary studies, reviews or theoretical papers which conceptualised, defined and/or operationalised social inclusion or occupational justice in mental illness or HIV.

Study appraisal and synthesis. We undertook a three-part article screening process. Screening and data extraction were undertaken independently by two researchers. Arksey's framework and thematic analysis informed the collation and synthesis of included papers.

Results. From 3352 records, we reviewed 139 full articles and retained 27 in this scoping review. Definitions of social inclusion and occupational justice in the domains of mental illness and HIV were heterogeneous and lacked definitional clarity. The two concepts were conceptualised as either processes or personal experiences, with key features of community participation, respect for human rights, and establishment and maintenance of healthy relationships. Conceptual commonalities between social inclusion and occupational justice were premised on social justice.

Conclusions To address the current lack of clarity, we propose further exploration of these two concepts together. More importantly among people with comorbid mental health disorders like substance use disorders and HIV from low-income contexts. The main features of that expansion should reflect contextual realities influencing community participation, respect of human rights, relational experiences and meaningful occupational participation. From this broadened understanding, quantitative measures should be used in evaluating interventions seeking to improve occupational justice and social inclusion.

For peer review only - http://bmjopen.bmj.com/site/about/guidelines.xhtml

Title: Occupational justice and social inclusion among people living with HIV and people with mental illness: A scoping review.

Article Summary

Strengths and limitations of this study

- The methodology as provided by the scoping review design, facilitated for comprehensive mapping of literature.
- We used a rigorous strategy to explore research foci, definitions and utilisations of the concepts of social inclusion and occupational justice in mental illness and HIV.
- Data synthesis was limited to work published in English originally or with available English translated copies.
- We focused on mental illness, which is made up of several different conditions and could have introduced generalisation bias. However, most of the included studies were also not condition specific fitting our primary aim for conceptual review.
- Focus was limited to conceptual and theoretical aspects of the concepts more than interventions and outcomes of interventions.

For peer review only

For peer review only - http://bmjopen.bmj.com/site/about/guidelines.xhtml

Introduction -

The global burden of disease from HIV remains substantially high with about 37.9 million people living with HIV by World Health Organisation estimates of 2018. However, in the last two decades, the world has seen a combination of a significantly decreased mortality and a small decrease in incidence leading to an increase in the number of people living with HIV from 8·74 million (1990) to 36·82 million (2017).[1] Nevertheless, in some key populations, this decline is being impacted by the rising and ever complex relationship between mental illness and HIV.[2 3] The prevalence of common mental disorders has been shown to be significantly higher among people living with HIV despite being on ART and is further impounded by stigma.[4]

People with chronic and usually stigmatised conditions such as mental illnesses, physical disabilities and HIV, face barriers to full participation in their communities.[5] Poverty, lack of education, lack of suitable housing, and unemployment are some of the social and economic barriers to accessing adequate and sustained healthcare faced by this group.[6] These barriers may similarly be experienced by people who face discrimination based on their class, race or gender identity or sexuality and thus, when persons with mental illness also share these characteristics they may be severely stigmatised or discriminated against.[7 8]

Social inclusion and occupational justice as concepts are relevant to direct research and practice toward the moral imperative to address exclusions and injustices experienced by stigmatised groups[9]. Focus on these outcomes ensure that health and social well-being are addressed beyond the medical management of the disease. Health-related quality of life as a holistic construct for promoting continuum of care and health and well-being beyond viral suppression in HIV,[10 11] including social inclusion and occupational justice. These concepts direct the health and social care community to foreground social justice issues of people with mental illness and HIV as part of marginalized groups at risk of being deprived of respect, rights and opportunities to achieve optimal health-related quality of life.

Social inclusion and occupational justice are potentially key in informing the promotion of human-rights based, sustainable person and community-centred interventions that promote recovery for persons with chronic, and stigmatised conditions.[12 13] In order to aid integration, and operationalisation of occupational justice and social inclusion in practice, we need to understand how the concepts are conceptualised and applied in population groups affected by chronic and stigmatised conditions. Synthesised summaries of research evidence can inform

For peer review only - http://bmjopen.bmj.com/site/about/guidelines.xhtml

primary research and implementation science ,[14] therefore we selected a scoping review design to advance this field.[15] This was a particularly appropriate method for this area due to the diverse disciplinary location of existing literature.[16 17] This scoping review aimed to explore and appraise the definitions, current utilisation, and relationships between the concepts of social inclusion and occupational justice in mental illness and HIV literature.

Methods

This scoping review followed our published study protocol[15], developed using Arksey and O`Malley`s Scoping Review framework,[17] as well as guidelines for scoping review protocols in occupational therapy .[14 18] In this paper, a scoping review is taken to be a form of knowledge synthesis that addresses an exploratory research question rather than the highly focused question in a systematic review. [16 17] For reporting, we followed the Preferred Reporting Items for Systematic reviews and Meta-Analyses extension for Scoping Reviews (PRISMA-ScR) Checklist. [19]

We followed an iterative process to develop and refine the research question.[17] Based on the subject area terminology, literature, and our understanding of current practice trends in managing conditions that are chronic, and stigmatised, we asked the following question:

How are occupational justice and social inclusion conceptualised, defined, and operationalised, and how are these concepts related in the highly stigmatised chronic conditions of mental illness and HIV?

The objectives of our scoping review were:

- To identify articles that define or conceptualise occupational justice and social inclusion related to mental illness or HIV.
- To describe how these are operationalised or utilised.
- To identify and describe relationships between occupational justice and social inclusion.
- To determine potential areas for further development, integration, and application of these concepts.

For peer review only - http://bmjopen.bmj.com/site/about/guidelines.xhtml

Search Strategy

With the aid of a subject librarian, we identified appropriate databases using a journal indexing system. We searched twelve databases in January 2018: PubMed, Scopus, Academic Search Premier, the Cumulative Index to Nursing and Allied Health Literature (CINAHL), Africa-Wide Information, Humanities International Complete, Web of Science, PsychInfo, and SocINDEX, Grey literature Report, Web of Science Conference Proceedings, and Open Grey. We used PubMed as the free platform for accessing articles indexed on Medline database. The selected databases captured a comprehensive sample of literature from a variety of disciplines including social work, psychiatry, nursing and occupational therapy.

The first and last authors (CN & RG) worked with the librarian, through an iterative process, to develop an inclusive list of search terms and applicable filtering methods including Boolean phrases and MESH terms for each database.[15] Primary search terms related to the primary concepts of occupational justice and social inclusion while secondary search terms encompassed the broader terms of mental health, occupational therapy, mental illness, HIV, and rehabilitation. For the purposes of search strategy development, we restricted our search to literature published between January 1997 and 31 January 2019 a period which has seen the emergence and rapid growth in literature on occupational justice.[20] We conducted a preliminary search on PubMed and this enabled refinement of our search strategy to maximise sensitivity and specificity. We adapted the PubMed search strategy (Appendix 1 – provided as an online supplementary appendix) accordingly for other databases.

Study selection

The first author (CN) reviewed the titles identified in the search for eligibility. The aim was to identify articles that i) indicated a research focus on mental illness, or HIV, or both and ii) titles that included the key terms of occupational justice, social inclusion or both. Article types included primary studies, reviews, opinion papers and other theoretical papers without primary data. Articles were not eliminated where there was uncertainty with the title until it was examined more in-depth by looking at the abstract. Two independent reviewers, the first and third authors (CN & EM), reviewed titles and abstracts of preliminarily selected articles using predetermined inclusion and exclusion criteria detailed in our protocol.[15] These same reviewers each further screened full-text articles to determine if they met the inclusion/exclusion criteria. At this stage, articles were included if their explicit focus was on social inclusion or occupational justice in mental illness and or HIV; there was definitional part for the concepts and reported some operationalisation of the two concepts. Discrepancies were resolved by

For peer review only - http://bmjopen.bmj.com/site/about/guidelines.xhtml

consensus or by seeking adjudication from the second author (LL). The Cohen's κ statistic to determine inter-rater agreement was calculated for the title and abstract review and the full article review stage, giving more than 90% agreement between reviewers with Cohen's κ statistic of 0.78 and 0.83 respectively.

Data Extraction

A common extraction table was designed, guided by the study objectives, to extract standard bibliometric information study characteristic and main findings. The first five articles were reviewed by both the first and third authors, with the remaining articles divided between the two authors. We then checked for accuracy and completeness against each other's work. Discrepancies were resolved by revisiting the article, discussing, and reaching consensus.

For peer review only

For peer review only - http://bmjopen.bmj.com/site/about/guidelines.xhtml

Data Synthesis

Data were synthesised descriptively to give a structured summary of the dataset and to capture the characteristics of the studies included and the definitional range of social inclusion and occupational justice. Study grouping followed the publication trends over time and study designs used. Descriptive statistics were calculated using Microsoft Excel 2016 for frequencies. We used deductive thematic analysis to organise the extracted definitions and related concepts for occupational justice and social inclusion.

Results

Retained studies' characteristics

As described in Figure 1, we screened (n=3352) titles and after reviewing (n=139) full articles, (n=27) were included in this scoping review. Of the (n=27) sources included for final review, (n=23) were published between 2009-2018, with the majority of these (n=6) published in 2012 (Table 1). Most publications were by authors in the mental health field and from high income countries, with 68% of the primary studies being conducted in Europe[21-33], and 9% in Australia.[34 35] No primary studies were conducted in North America, Africa, or Asia. More than a third of the studies (n=10) utilised a qualitative research design[22-25 28-30 32 34 36 37], five (16%) utilised a quantitative research design[21 26 27 33 35] and only one study utilised a mixed methods design.[31] Six (19%) were review papers and the remainder (n=5) were opinion[38 39], lectureship[40], commentary[41] and theoretical analysis papers.[42] The two concepts were predominantly explored around mental illness (n=26) with less focus on HIV (n=1). The majority (n=21) of the published research investigated social inclusion as it related to mental health conditions, and all the occupational justice papers were focussed on mental illness. Only one paper was included which discussed social inclusion in relation to people living with HIV.[43] We found no published literature that explored occupational justice and social inclusion in populations with comorbid mental illness and HIV.

Insert Figure 1: **Scoping review flow diagram

For peer review only - http://bmjopen.bmj.com/site/about/guidelines.xhtml

Table 1: Summary of articles defining social inclusion and occupational justice in mental illness and HIV

Author(s) Year	Participants<br><br>Setting	Design	Aim	Definition(s) of social inclusion and or occupational justice	How operationalized	Associated key terms used
2a: Studies with primary data collection						
Mazzi *et al.*<br><br>2018	30 people with non-affective psychosis. In a social inclusion intervention program<br><br>Italy	Cross-sectional study	"To assess whether a social inclusion intervention is associated with better outcomes in terms of personal and social recovery, with particular reference to the areas of social functioning and activity, and subjective dimensions such as self-esteem, self-stigma and perceived quality of life" (p.1)	Social inclusion is the opportunity for an individual to participate in key functions or activities and in the economic, social and cultural life of his/her community, exercising the rights of his/her citizenship and enjoying an adequate standard of living and wellbeing.	As an outcome and intervention<br>As a human rights issue	social withdrawal, quality of life, discrimination, social recovery, social inclusion activities, social disadvantage
Saavedra *et al.*<br><br>2018	31 service users with severe mental illness. In workshops for social integration<br><br>Spain	Mixed methods study	To evaluate the impact of an artistic workshop on a group of people diagnosed/screened for with severe mental illness with focus on the impact of creative practices on well-being and social inclusion outcomes.	Social inclusion stated as a personal construct measured through perception of social isolation, social relationship and social acceptability	As an outcome to be evaluated based on personal perception<br><br>Measured using Social Inclusion Questionnaire	stigma, wellbeing, recovery, social isolation, social relationship, social acceptability

For peer review only - http://bmjopen.bmj.com/site/about/guidelines.xhtml

Table 1: Summary of articles defining social inclusion and occupational justice in mental illness and HIV						
**Author(s) Year**	**Participants**<br><br>**Setting**	**Design**	**Aim**	**Definition(s) of social inclusion and or occupational justice**	**How operationalized**	**Associated key terms used**
Berry & Greenwood<br><br>2017	51 young outpatient service users with first episode psychosis. In Early Intervention in Psychosis, Community Mental Health and Assertive Outreach services<br><br>United Kingdom	Longitud-inal study	"To investigate the direct and indirect associations between dysfunctional attitudes, self-stigma, hopefulness, social inclusion and vocational activity for young people with psychosis" (p.197).	Social inclusion comprises social activity and community belonging.	As measured by levels of social activity and community belonging using Social Relationships Scale and Social Inclusion Scale	self-stigma, hopefulness, social activity, community belonging, social network, social contact, vocational activity
Turner *et al.*<br><br>2017	71 people with psychotic-related conditions. In a research programme on schizophrenia<br><br>Ireland	Quantita-tive descriptive study	To explore "the level of social inclusion among people with psychotic-related conditions using a standardised interview" (p.195)	Social inclusion is a multi-dimensional phenomenon with a number of domains including socially valued role functioning, social support, absence of stigma, and integration in rehabilitation community and wider community	As a multi-dimensional phenomenon<br>As both objective and subjective outcome<br>Assessed using Social Inclusion Interview Schedule	supportive relationships, stigma, integration, social exclusion, social support, rehabilitation.

For peer review only - http://bmjopen.bmj.com/site/about/guidelines.xhtml

Table 1: Summary of articles defining social inclusion and occupational justice in mental illness and HIV

**Author(s) Year**	**Participants**<br><br>**Setting**	**Design**	**Aim**	**Definition(s) of social inclusion and or occupational justice**	**How operationalized**	**Associated key terms used**
Raitakari *et al.*<br><br>2016	16 mental health service users. In two mental health floating support services (FSS)<br><br>Finland	Qualitative study	To explore how community integration is understood and tackled in mental health floating support services (FSSs) and, more precisely, in service user–practitioner home visit interaction	Social inclusion is articulated in political discourse as a way to tackle social exclusion and to increase citizens' participation and activity in society	As supported integration in community life.<br><br>As participation in what marginalised individuals are usually excluded from	social exclusion, citizenship, community integration, housing, interaction, mental health, recovery
Killapsy *et al.*<br><br>2014	67 mental health service users with psychosis. Living in the boroughs of London and the inner-city<br><br>United Kingdom	Quantita-tive study	To investigate change in social inclusion after the development of a psychotic illness (clinically diagnosed) and associated factors	"Social inclusion refers to the opportunities that individuals have to participate in key areas of economic, social and cultural life" (p.148)	Focus on participation and access to services and opportunities<br><br>Five domains of social inclusion: social integration, consumption, access to services, productivity, political engagement<br>Assessed using the Social Inclusion Questionnaire User Experience	social exclusion, social disadvantage, social integration, employment, social isolation

For peer review only

Table 1: Summary of articles defining social inclusion and occupational justice in mental illness and HIV

Author(s) Year	Participants<br><br>Setting	Design	Aim	Definition(s) of social inclusion and or occupational justice	How operationalized	Associated key terms used
Salles & Barros<br><br>2013a	17 mental health service users and 12 individuals from their social networks. In Psychosocial Care Centres<br><br>Brazil	Qualitative study	"To identify and analyse the conceptions expressed by the interviewees about social inclusion and mental illness" (p.37)	"Social inclusion is a process of promoting rights, access, choice and participation. For individuals with mental health problems, this also means access to the best possible forms of treatment" (p.37)	As a dynamic, multi-dimensional process.	work participation poverty
Salles & Barros<br><br>2013b	17 mental health service users and 12 individuals from their social networks. In Psychosocial Care Centres<br><br>Brazil	Qualitative study	"To identify the daily life experiences of users of a psychosocial care center related to processes of social exclusion and inclusion" (p.704).	Social inclusion is a process of the individual's choice on how to live her daily life" (p.710) and not just about experiencing full participation in society.	As a multidimensional process<br>As a human rights issue<br>As subjective experience centred on work participation	social exclusion, discrimination, prejudice, stigma, social isolation, social participation, empowerment, poverty

For peer review only

For peer review only - http://bmjopen.bmj.com/site/about/guidelines.xhtml

Table 1: Summary of articles defining social inclusion and occupational justice in mental illness and HIV

**Author(s) Year**	**Participants**<br><br>**Setting**	**Design**	**Aim**	**Definition(s) of social inclusion and or occupational justice**	**How operationalized**	**Associated key terms used**
Clewes *et al.*<br><br>2013	Single Case-study with bipolar depression. In occupational therapy interventions under UK National Health Services (NHS)<br><br>United Kingdom	Case study	To illustrate how the combination of medical outpatient clinic and occupational therapy intervention together made a big difference in a person's life.	Social inclusion was taken as meaningful inclusion in the areas of life where the participant wished to be.	As personal experience.<br><br>As a degree of autonomy, ownership, responsibility, and independence.	stigma, recovery, social policy, empowerment, rights, engagement, spirituality, client leading.
Nieminen *et al.*<br><br>2012	23 mental health service users. In an intervention group follow up study<br><br>The European Union	Qualitative study	To describe how the mental health service users experienced social inclusion and employment in the European Union EMILIA project	Social inclusion is a subjective sense of belonging and active citizenship that enhances social integration	As an experience and feeling of active citizenship	empowerment, social network, finance and housing employment. social exclusion. stigma, prejudice
Fieldhouse<br><br>2012	Eight mental health service users in a two-year action research project<br><br>United Kingdom	Qualitative research	To describe an action research project that explored the recovery journeys of a group of assertive outreach service users who had progressed from being socially excluded and occupationally deprived to being participants in their local communities and to use this knowledge to inform local service development	Social inclusion is when people with mental health challenges enjoy rewarding social relations through renewed engagement in mainstream occupations resulting in fuller community participation.	As active participation in context or local community<br><br>As an outcome of occupation-based interventions.	social participation, social relationships, stigma, social exclusion, community participation, belonging, social capital

For peer review only - http://bmjopen.bmj.com/site/about/guidelines.xhtml

Table 1: Summary of articles defining social inclusion and occupational justice in mental illness and HIV

**Author(s) Year**	**Participants**<br><br>**Setting**	**Design**	**Aim**	**Definition(s) of social inclusion and or occupational justice**	**How operationalized**	**Associated key terms used**
Stain *et al.*<br><br>2012	1825 adults with psychosis in an Australian national survey of psychosis.<br><br>Australia	Quantitative study	"To explore the impact of psychosis on an individual's social and community participation" (p. 879)	"Social inclusion refers to the participation of a person in society and is evidenced by an individual having the opportunities, resources and abilities to build and maintain relationships, engage in education and employment, and participate in community events and organisations" (p.880)	As participation in mainstream life as empowered individuals	social isolation, social anxiety, stigma, social participation, community participation
Smyth *et al.*<br><br>2011	Eight mental health service users in mental health rehabilitation services in inner-city area<br><br>United Kingdom	Qualitative study	To explore the experiences of social inclusion for mental health service users and factors associated when engaging in everyday community occupations	Social inclusion is developing fair access to opportunity in key social and economic spheres for marginalised groups.	As an issue of participation and access to services and opportunities	stigma occupational deprivation, discrimination, social networks
Ramon *et al.*<br><br>2011	27 key informants in an evaluation study of the EMILIA project<br><br>The European Union	Qualitative study	To identify how participation in the EMILIA project affects the lives of mental health service users in relation to social inclusion, employment and recovery	Social inclusion is a subjective sense of belonging and active citizenship that enhances social integration	As an experience and feeling about participation in society	employment, well-being, quality of life, social networks.

For peer review only - http://bmjopen.bmj.com/site/about/guidelines.xhtml

Table 1: Summary of articles defining social inclusion and occupational justice in mental illness and HIV

Author(s) Year	Participants<br><br>Setting	Design	Aim	Definition(s) of social inclusion and or occupational justice	How operationalized	Associated key terms used
Hamer *et al.*<br><br>2017	82 mental health service users<br><br>New Zealand & Brazil	Qualitative study	To present service users' stories of distressing exclusion that interrupted their rights to occupational justice, and marginalised them from occupation.<br>The paper also presents the practices of inclusion that service users engaged in that restored their rights and responsibilities as occupied and active citizens	Social inclusion is the extent to which people are confident about and able to exercise their rights and participate, by choice, in the ordinary activities of citizens<br><br>Occupational justice recognises the person's right to inclusive participation in everyday occupations regardless of age, ability, gender, social class, or other differences.	Social inclusion as a policy issue<br>As a dynamic concept, derived from subjective experiences<br><br>Social inclusion is enhanced through occupational justice<br><br>As inclusive participation<br>As a human right issue	occupational injustice, citizenship, stigma, discrimination
Fieldhouse<br><br>2012	Eight mental health service users in a two-year action research project<br><br>United Kingdom	Qualitative Study	To examine the impact of community participation on their recovery and social inclusion and how service users' experiences informed joint planning between mental health services and the learning community to promote social inclusion.	Social inclusion as the process of enabling citizenship through fuller community participation.<br><br>Occupational justice defined as the process of lobbying for the occupational needs of individuals and communities as part of a fair, inclusive, and empowering society; as a community reintegration issue.	As active participation in mainstream society	social participation, social relationships, stigma, social exclusion, community participation, belonging

For peer review only - http://bmjopen.bmj.com/site/about/guidelines.xhtml

Table 1: Summary of articles defining social inclusion and occupational justice in mental illness and HIV						
**Author(s) Year**	**Participants**<br><br>**Setting**	**Design**	**Aim**	**Definition(s) of social inclusion and or occupational justice**	**How operationalized**	**Associated key terms used**
Farrell & Bryant<br><br>2009	Nine recruiters of volunteers who had mental illness.<br><br>United Kingdom	Qualitative study	To explore the recruiters' understanding of mental health problems, drawing on their experiences	Occupational justice is an intrinsic part of social justice, permitting equitable opportunity and the means to choose, organise and perform meaningful occupations<br><br>Social inclusion stated but not defined	As a process and an outcome<br><br>Occupational justice as an intrinsic aspect of social inclusion	volunteering, discrimination, stigmatization, social exclusion, social attitudes, prejudice, occupational deprivation, occupational marginalisation, occupational apartheid
2b: Review, commentary, lectureship and opinion papers						
Le Boutillier & Croucher<br><br>2010	Mental health service users	Opinion paper	To present an alternative to the polarised view of social inclusion	Social inclusion is a multidimensional virtuous circle aimed at improving rights of access to the social and economic world, new opportunities, recovery of social identity and meaningful life and also reduced impact of disability on everyday life.	As a multidimensional system	Social exclusion, occupational justice, occupational balance, occupational alienation, occupational deprivation

For peer review only - http://bmjopen.bmj.com/site/about/guidelines.xhtml

Table 1: Summary of articles defining social inclusion and occupational justice in mental illness and HIV

**Author(s) Year**	**Participants**<br><br>**Setting**	**Design**	**Aim**	**Definition(s) of social inclusion and or occupational justice**	**How operationalized**	**Associated key terms used**
Cobigo & Stuart<br><br>2010	Mental health service users	Review	To review recent research on approaches to improving social inclusion for people with mental disabilities	Social inclusion is when one feels accepted and recognized as an individual beyond the disability; has positive personal relationships with family, friends, and acquaintances; is actively involved in recreation, leisure, and other social activities; has appropriate living accommodations; has healthy employment;<br>and has appropriate formal (service system) and informal (family and caregiver) supports.	As an acceptance, human rights, outcome of interventions issue	stigma, discrimination, legislation, community support, disability rights, justice, human rights
Caceres *et al.*<br><br>2008	Global literature on MSM with HIV	Review	To analyse reasons for continued risk of HIV and its consequences in MSM globally	"a social inclusion perspective on HIV prevention and AIDS care implies the adoption of a broad range of strategies to understand and confront social vulnerability" (p.11)	As a perspective to addressing vulnerability	social exclusion _ describes the alienation or disenfranchisement that certain individuals or groups experience within society.<br>stigma discrimination, prejudice, human rights, poverty, migration, employment participation, sexuality
Lloyd *et al.*<br><br>2006	Mental health service users	Review	To describe a selected number of activities that promote social inclusion.	Social inclusion involves being able to re-join or participate in leisure, friendship and work communities.	Participating and accessing services and opportunities	connectedness and interdependence

For peer review only - http://bmjopen.bmj.com/site/about/guidelines.xhtml

Table 1: Summary of articles defining social inclusion and occupational justice in mental illness and HIV

**Author(s) Year**	**Participants**<br><br>**Setting**	**Design**	**Aim**	**Definition(s) of social inclusion and or occupational justice**	**How operationalized**	**Associated key terms used**
Farone<br><br>2006	Mental health service users, with a focus on schizophrenia	Review	To examine empirical evidence describing experiences with social or community integration for people with psychiatric disabilities, with a particular interest in schizophrenia	Social inclusion discussed but not defined	A link to mental and emotional well-being	community integration, community inclusion, social integration, stigma, social support, social networks
Evans & Repper<br><br>2000	Mental health service users	Review	To challenge common misconceptions surrounding employment, work, and mental health problems of mental health service users.	Social inclusion is defined as a need, aspiration, and citizenship issue among mental health service users. Social inclusion as an outcome of work participation.	A social need. An outcome of work participation	social exclusion, stigma, unemployment, poverty
Mandiberg 2012	People with psychiatric disabilities	Commentary	To describe the failure of social inclusion as a concept and present an alternative approach through community development	Social inclusion refers to full participation in the broader community for people with severe mental illnesses	As experience of participation in the broader community	work integration, community development, social enterprises
Townsend<br><br>2012	Mental health service users<br><br>Canada	Lectureship	To propose for an interdisciplinary knowledge exchange with a critical occupational perspective on the question: What lessons on boundaries and bridges to adult mental health can be drawn by connecting the capabilities and occupational frameworks of justice?	Occupational justice as the enjoyment of the 'occupational rights' of all people to engage and be socially included in their desired occupations, and thereby to contribute positively to their own well-being and the well-being of their communities.	As a human rights, capabilities and justice issue	occupational rights, occupational possibilities occupational deprivation, alienation, imbalance and marginalisation

For peer review only - http://bmjopen.bmj.com/site/about/guidelines.xhtml

Table 1: Summary of articles defining social inclusion and occupational justice in mental illness and HIV						
**Author(s) Year**	**Participants**<br><br>**Setting**	**Design**	**Aim**	**Definition(s) of social inclusion and or occupational justice**	**How operationalized**	**Associated key terms used**
Harrison & Sellers<br><br>2008	Mental health service users and mental health team<br><br>United Kingdom	Opinion Paper	To explore the implications and challenges for occupational therapy roles in mental health services regarding socially inclusive practice and to discuss policy that is designed to broaden professional roles.	Occupational justice identifies inequalities in opportunities to participate in occupations.	As a human rights issue about participation in occupations<br><br>As a policy issue	social exclusion, participation, poverty, occupational deprivation
Hamer<br><br>2017	Mental health service users	Review	To discuss how social inclusion for mental health service users can be enhanced through occupational justice and the protection of their rights as citizens to have meaningful employment.	Social inclusion can be defined as the extent to which people are confident about and able to exercise their rights and participate, by choice, in the ordinary activities of citizens.<br><br>Occupational justice stated but not defined	As a human right issue	social exclusion, stigma, occupational justice, employment participation, poverty

For peer review only

Table 1: Summary of articles defining social inclusion and occupational justice in mental illness and HIV						
**Author(s) Year**	**Participants<br><br>Setting**	**Design**	**Aim**	**Definition(s) of social inclusion and or occupational justice**	**How operationalized**	**Associated key terms used**
Pettican & Bryant<br><br>2007	Mental health service users	Theoretical analysis	To explore the potential of occupational justice and its related concepts. To provide the occupational therapy profession with a theoretical justification for occupational therapists adopting an occupation-focused role in community mental health teams	Social inclusion is a drive aiming to overcome discrimination and stigma faced by people with mental health problems, in order to facilitate their having equal access to mainstream employment, education and leisure opportunities.<br><br>Occupational justice is recognising and providing for the occupational needs of individuals and communities as part of a fair and empowering society	Social inclusion conceptualized as a policy drive issue<br><br>As a justice and human rights issue<br>As an occupational participation issue	occupational deprivation, occupational imbalance, occupational alienation, social justice

For peer review only

Social inclusion: concepts and definitions

Social inclusion was defined with high variability, with only two studies using the same definition by defining social inclusion as a subjective sense of belonging and active citizenship that enhances social integration.[28 30] Conceptualisations and definitions used ambiguous words such as community, participation and integration to define social inclusion.. Some studies defined social inclusion in terms of paid work and participation in community events,[26 27] others focussed on social acceptance and absence of stigma,[28 30 33 44] while still others saw it as a political discourse.[29] Stain et al.[35] tried to capture these varieties, and defined social inclusion as

> "the participation of a person in society, evidenced by an individual having the opportunities, resources and abilities to build and maintain relationships, engage in education and employment, and participate in community events and organisations" (p.880).

The variations revealed notable thematic areas that emerged from the analysis of the definitions, namely community participation; human rights; and relational (relating to relations, and notions of acceptance and belonging).

Community participation

The most prominent shared features of the definitions of social inclusion in mental illness and HIV research focussed on it being a process and an experience centred on community participation. However, the terms 'community participation' and 'community' were used in many ways without clear description. These varied interpretations of community participation included reference to people with HIV or mental illness as: individuals with opportunities to participate in key activities in their communities like paid employment[26 27 35 45]; being integrated into the community[25 29 33]; having a sense of belonging within the community[21 28 30 39]; and exercising active citizenship.[27 43 46]

Human rights

Social inclusion was also defined and conceptualised as a human rights issue.[27 34 37 44 46] while still kept community participation as the penultimate indicator for social inclusion. When discussing human rights, the authors highlighted the right to use one's ability to access resources and opportunities for community growth. Social inclusion was also conceptualised

For peer review only - http://bmjopen.bmj.com/site/about/guidelines.xhtml

as the right to engage in productive occupations, with full access to work and or educational activities within the community despite one's health concerns.[46]

Relational

Social inclusion was further conceptualised and defined as a subjective experience[21 28 30 31 33 35] where those who are socially included should experience belonging in establishing, maintaining and experiencing positive relationships. While a number of authors[21 28 30 31 33 35] talked about relationships as being a key component, this was not defined or discussed in depth, instead it was emphasised that for social inclusion to be a reality, an individual should experience positive social relationships with their significant others, family, friends and acquaintances. [21 28 30 31 33 35] Social inclusion was also conceptualised as experiencing social support and positive support networks.[33]

Diversity in definitions reflected in measurement tools

The diversity in definitions and features was also present in instruments used. Most quantitative studies included a social inclusion measure, including: Social Inclusion Questionnaire,[31] Social Relationships Scale and Social Inclusion Scale,[21] Social Inclusion Interview Schedule,[33] and Social Inclusion Questionnaire User Experience.[26] Social inclusion was also portrayed in some definitions as a subjective personal concept where it is the individual with mental illness or person living with HIV, who subjectively experiences inclusion and should have a choice on what determines their experience of such inclusion.[22 28 30 32 39] It is their perception of the quality of their relationships, their acceptability to others, and how integrated they are, which was emphasised.

In summary, social inclusion was conceptualised as processes and experiences of empowered and equitable community participation for all, in which there is respect for human rights and healthy relational well-being is promoted.

Conceptualising and defining occupational justice

In the last two decades, only six research papers on mental illness used the concept of occupational justice and provided a definition. [23 24 34 38 40 42] (Table 1) Five different definitions were found (Table 1). As with social inclusion, occupational justice was defined with great variability, with it being referred to as both a process and as an experience. [23 24 34 38 40 42] Two major themes emerged pointing to social justice in which occupational justice was framed as an occupational rights issue and as a matter of community participation.

For peer review only - http://bmjopen.bmj.com/site/about/guidelines.xhtml

Occupational rights

Occupational justice as experiencing or enjoying one's occupational rights [23 24 34 38 40 42] emerged as key to conceptualizing and defining occupational justice for persons with mental illness. Where occupational rights were taken to mean people's rights to participate in a range of meaningful and contextual occupations enabling them to flourish, fulfil their potential and experience life satisfaction in ways consistent with their contexts. Townsend (2012) defined occupational justice as the enjoyment of 'occupational rights' by all people to engage in occupations and feel socially included in their desired occupations, thereby contributing positively to their own well-being and to that of their communities.[40] Occupational justice was also highlighted as an advocacy process of lobbying for the occupational rights and needs of individuals and communities as part of an equitable, inclusive, and empowering society.[24]

Community participation

The process of promoting occupational justice was viewed as related to promoting social inclusion and community participation through advancing participation.[34] In another paper community participation was emphasised, with occupational justice defined as situations when people are seen as having the opportunity to choose to participate in the community.[23] The core emphasis in the definitions was the acknowledgement of occupational justice as a means of actioning community participation and social justice.[23 34]

However, in all the definitions there was an overuse of the term occupation. While occupation was framed as purposeful everyday activities people engage in, it carries diverse meanings outside occupational science and occupational therapy. This rendered many of the definitions and arguments cyclical in nature.

Commonalities between social inclusion and occupational justice

** Insert Figure 2: **Commonalities between occupational justice and social inclusion definitions – associated terms**

Although not clearly articulated, all the papers which discussed the two concepts together seemed to infer that occupational justice was an important ingredient of social inclusion.[23 24 34 40] Notwithstanding the considerable variability in definitions and conceptualisations of the two concepts, some commonalities were identified (Figure 2). Both social inclusion and

For peer review only - http://bmjopen.bmj.com/site/about/guidelines.xhtml

occupational justice seek to promote equitable access to opportunities for engagement and to seek for fairness and justice in individual's community participation.[23 24 34 40]

Hamer et al. provided the closest account of the relationship between the concepts positing that social inclusion was enhanced through occupational justice.[34] Here they argued that recognition of people's right to inclusive participation in everyday occupations (occupational justice) enhanced the extent to which the person became confident about and were able to exercise their rights and participate by choice in the ordinary activities as citizens (social inclusion).[34] Both concepts were centred on key thematic areas of human rights, equality, inclusivity, and community participation.

Significantly, both social inclusion and occupational justice had a social justice focus, emphasizing the right to inclusive participation in a community and individuals exercising choice of participation as part of their citizenry beyond their health conditions. Occupational justice specifies that the participation in meaningful occupations is central, while social inclusion highlights community participation in general. Both concepts also highlight the need to address discriminatory practices, by doing away with stigma.[33 34] Hamer and colleagues highlight that social inclusion is the process of experiencing inclusive participation in the community as a citizen, while occupational justice promotes social inclusion through participation in meaningful and valued activities.[34]

Discussion

We explored and appraised the definitions, current utilisation, and relationships between social inclusion and occupational justice in literature on mental illness or HIV. We identified and critically appraised 27 articles that presented variety of definitions. Most studies were on mental illness and conducted in high-income countries. Despite the great variability, key thematic concepts used to define social inclusion and occupational justice included community participation, human rights, and relationships. The two concepts are theoretically related with a social justice focus. There is also emphasis on the multidimensional nature of the concepts framed as both a process and a personal experience. The main source of these definitions being qualitative studies using service user's experiences and experts opinions.

While definitions of social inclusion vary, our scoping review findings confirm that the definitions are still in line with the original concept, that of poverty reduction with a focus on reducing stigma and discrimination.[32 33 43] Social inclusion emerged from European societies, in response to a welfare crisis and desire to fight disadvantage.[47] We propose that this focus

For peer review only - http://bmjopen.bmj.com/site/about/guidelines.xhtml

on welfare and fighting disadvantage should remain and should be taken up in the mental health and HIV fields, consistent with poverty reduction, justice, and equality as pillars of social inclusion.

The most striking observation was the lack of clarity in the definition of social inclusion, which is still evident, despite its existing for almost five decades. Efforts to be all inclusive, multidimensional, person-centred and contextual can explain the variations and, thus, ambiguity in the definitions.[47 48] However, this lack of definitional clarity could hamper its universal concept utilisation, measurement, and further exploration with a common goal.[49 50] This lack of a single, universal understanding has positive and negative implications for research and application of the concept in clinical practice. Without a universally agreed upon definition, comparisons between studies and practice remains difficult.[50] This was echoed in the variety of social inclusion measures applied in the quantitative studies.[21 26 31 33] Given the variation in measures, a common understanding of what constitutes social inclusion in mental health has not been developed, despite calls for this in recent literature.[49-51] Therefore construct validity of measures is difficult to test. In contrast, the variations in defining the concepts reflects diversity and the importance of contexts, rather than a singular adoption of a "universal" idea. Given the diversities in experiences of mental illness and living with HIV, where different regions have their own social, economic, political, cultural, and historical realities influencing mental illness and HIV, conceptualisations of social inclusion may benefit from remaining open to multiple definitions in order to reflect the realities of different regions. It is possible to have multiple, but mutual interpretations that could be understood as complementary or even contesting, in line with contextual realities.

Embracing plural definitions may be especially important in low and middle-income countries, where such research needs further growth.[47] For example different activities purported to enhance social inclusion, like paid employment, have different meanings and impact across regions and would influence how it is conceptualised as part of social inclusion. We found a preference towards paid employment as a key determinant of community participation in social inclusion.[28 35 37 45 52] This can show the dominant knowledge systems of capitalist societies[47] where most of the studies were conducted. Other communal ways of engagement outside of a neo-liberal market structure could be explored as they may facilitate new ways of understanding inclusion.

For peer review only - http://bmjopen.bmj.com/site/about/guidelines.xhtml

Some of the social inclusion definitions had an individual focus, for example the individual had to be actively participating at the expense of the collective found in communal societies.[26 27 31] Given that the studies were mainly from high-income regions, there remains some missing voices in informing the definitions, given the largely communal orientation found in indigenous communities in low and middle-income settings. The challenge in some of the reviewed definitions was to try to focus on the individual and the impact of disease, yet the social justice agenda may better be approached from a population level with a focus on broader social determinants of health which has been the case in many countries addressing developmental and intellectual disabilities. [53] Defining social inclusion from the perspective of people who experience poverty, unemployment, social inequality and forms of violence affecting collectives rather than individuals can strengthen the concept of social inclusion and promote its utilisation in such contexts.

Though the concept of occupational justice has been present in the literature for about three decades,[20] we found limited evidence of its conceptualisation and application in mental health and no studies in HIV. [23 24 34 38 40 42] The studies found were also from high-income countries, despite the global-justice theoretical orientation of the concept.[23 24 34] We found key features that could guide occupational justice utilisation and further theorisation: community participation by having one's occupational rights upheld, occupational needs met, empowerment and equity in occupational participation. To some extent, the lack of diversity in regions informing the concept offers an opportunity to strengthen the concept by adding insights from contexts with potential for different realities, experiences and viewpoints, such as Africa. Also some authors conceptualised occupational justice as participation in occupation(s) in an equitable manner[34], they took participation as synonymous with justice, without spelling the nature of the occupation and position of the person accessing the occupations. These have great potential in influencing how the accessed occupation impacts health, well-being and feeling of social inclusion.

Despite the definitional lack of clarity, social inclusion and occupational justice are related concepts that can be used together to frame research and practice. The commonality between the concepts is the need to promote equitable access to opportunities for community participation with fairness and equity. [23 24 34 40] The relationship between the concepts could be further developed using diverse communities to build evidence on how engagement in meaningful everyday activities underlie inclusive communities for people with mental illness and living with HIV. Focus on occupational justice presents a perspective on the nuances of

For peer review only - http://bmjopen.bmj.com/site/about/guidelines.xhtml

everyday occupational participation and what that means for the social inclusion of those engaging should be routinely explored.

We therefore propose an expansion of the relationship between the conceptualisation of the two concepts, using most affected population groups, such as people with comorbid mental health disorders like substance use disorders and HIV in low-income contexts. This population group is known to have unmet broader health and social care needs hinged to the double stigma associated with substance abuse and HIV.[54] That expansion should reflect the contextual realities influencing community participation, respect of human rights, and having healthy relationships, actioned through engagement in meaningful occupations. Measures with a broader scope and cross-cultural validation like the Social and Community Opportunities Profile (SCOPE),[55 56] can be instrumental in developing standardized measures.

Ethics and dissemination

Since the scoping review methodology consists of reviewing and collecting data from publicly available literature, this scoping review did not require ethics approval. Dissemination of findings is ongoing through peer-reviewed journals; seminars and conferences; targeting clinicians, academics, researchers, service users and policy makers.

Patient and public involvement:

Patient and public involvement (PPI) representatives were not directly involved in the design of this scoping review protocol. However, experiences of the first author in working with adults afflicted with HIV and mental health disorders in Zimbabwe informed the need to explore issues faced by this population beyond biomedical care. We also built our research question from insights being generated in his doctoral studies exploring occupational perspectives on social inclusion among young adults dually afflicted with substance use disorders and HIV. Social inclusion speaks to life beyond medical management which was not being given sufficient attention and hence the need to conduct a scoping review.

Conclusion

To our knowledge, this scoping review is the first to appraise the concepts of occupational justice and social inclusion in populations afflicted by mental illness and HIV. Our findings have the potential to initiate critical conversations in the field and expand understanding and utilisation of occupational justice and social inclusion to critique and enhance global mental health. We have also presented commonalities which will give us a better theoretical

For peer review only - http://bmjopen.bmj.com/site/about/guidelines.xhtml

foundation to inform further research, practice, and training, especially from underrepresented societies.

Author Contributions:

All authors have made substantive intellectual contributions to the conduct and write-up of this review. CN and RG conceptualised the review approach and provided general guidance to the research team. Then CN and EM were involved in systematic and independent screening and data extraction. CN provided primary input at all stages, developed all draft documents and had overall responsibility for the review. LL, RG and RH gave substantial review and critique through the review process and manuscript. All the authors reviewed and commented on the drafts of the manuscript and they all read and approved the final manuscript.

Acknowledgements:

We would like to thank Mary Shelton – UCT librarian for assistance in developing our search strategy. This article also benefited from writing soft skills and reviews by Dr Helen Jack through African Mental Health Research Initiative (AMARI).

Funding: This work was supported through the DELTAS Africa Initiative [DEL-15-01]. The DELTAS Africa Initiative is an independent funding scheme of the African Academy of Sciences (AAS)'s Alliance for Accelerating Excellence in Science in Africa (AESA) and supported by the New Partnership for Africa's Development Planning and Coordinating Agency (NEPAD Agency) with funding from the Wellcome Trust [DEL-15-01] and the UK government. The views expressed in this publication are those of the author(s) and not necessarily those of AAS, NEPAD Agency, Wellcome Trust or the UK government.

Competing interests: None.

References

1. Pandey A, Galvani AP. The global burden of HIV and prospects for control. *The Lancet HIV* doi: 10.1016/S2352-3018(19)30230-9
2. Bauer-Staeb C, Jorgensen L, Lewis G, et al. Prevalence and risk factors for HIV, hepatitis B, and hepatitis C in people with severe mental illness: a total population study of Sweden. *Lancet Psychiatry* 2017;4(9):685-93. doi: 10.1016/s2215-0366(17)30253-5 [published Online First: 2017/07/09]
3. Hughes E, Bassi S, Gilbody S, et al. Prevalence of HIV, hepatitis B, and hepatitis C in people with severe mental illness: a systematic review and meta-analysis. *Lancet Psychiatry* 2016;3(1):40-48. doi: 10.1016/s2215-0366(15)00357-0 [published Online First: 2015/12/02]
4. Lowther K, Selman L, Harding R, et al. Experience of persistent psychological symptoms and perceived stigma among people with HIV on antiretroviral therapy (ART): a systematic review.

For peer review only - http://bmjopen.bmj.com/site/about/guidelines.xhtml

Int J Nurs Stud 2014;51(8):1171-89. doi: 10.1016/j.ijnurstu.2014.01.015 [published Online First: 2014/03/08]
5. Jackson-Best F, Edwards N. Stigma and intersectionality: a systematic review of systematic reviews across HIV/AIDS, mental illness, and physical disability. *BMC Public Health* 2018;18(1):919. doi: 10.1186/s12889-018-5861-3
6. Aidala AA, Wilson MG, Shubert V, et al. Housing Status, Medical Care, and Health Outcomes Among People Living With HIV/AIDS: A Systematic Review. *American Journal of Public Health* 2016;106(1):e1-e23. doi: 10.2105/ajph.2015.302905
7. Breuer E, Myer L, Struthers H, et al. HIV/AIDS and mental health research in sub-Saharan Africa: a systematic review. *Afr J AIDS Res* 2011;10(2):101-22. doi: 10.2989/16085906.2011.593373 [published Online First: 2011/06/01]
8. Levi-Minzi MA, Surratt HL. HIV stigma among substance abusing people living with HIV/AIDS: implications for HIV treatment. *AIDS patient care and STDs* 2014;28(8):442-51.
9. Nilsson I, Townsend E. Occupational justice—Bridging theory and practice. *Scandinavian journal of occupational therapy* 2010;17(1):57-63.
10. Lazarus JV, Safreed-Harmon K, Barton SE, et al. Beyond viral suppression of HIV – the new quality of life frontier. *BMC Medicine* 2016;14(1):94. doi: 10.1186/s12916-016-0640-4
11. Kall M, Marcellin F, Harding R, et al. Patient-reported outcomes to enhance person-centred HIV care. *The Lancet HIV* doi: 10.1016/S2352-3018(19)30345-5
12. Baumgartner JN, Burns JK. Measuring social inclusion-a key outcome in global mental health. *Int J Epidemiol* 2014;43(2):354-64. doi: 10.1093/ije/dyt224
13. Patel V, Saxena S, Frankish H, et al. Sustainable development and global mental health—a Lancet Commission. *Lancet* 2016;387(10024):1143-45. doi: 10.1016/S0140-6736(16)00208-7
14. McKinstry C, Brown T, Gustafsson L. Scoping reviews in occupational therapy: The what, why, and how to. *Australian occupational therapy journal* 2014;61(2):58-66.
15. Nhunzvi C, Langhaug L, Mavindidze E, et al. Occupational justice and social inclusion in mental illness and HIV: a scoping review protocol. *BMJ open* 2019;9(3):e024049.
16. Grant MJ, Booth A. A typology of reviews: An analysis of 14 review types and associated methodologies. *Health Info Libr J* 2009;26:91-108. doi: 10.1111/j.1471-1842.2009.00848.x
17. Arksey H, Malley LO. Scoping Studies: Towards a Methodological Framework. *Int J Soc Res Methodol* 2005;8(1):19-32. doi: 10.1080/1364557032000119616
18. Halas G, Schultz AS, Rothney J, et al. A scoping review protocol to map the research foci trends in tobacco control over the last decade. *BMJ open* 2015;5(1):e006643.
19. Tricco AC, Lillie E, Zarin W, et al. PRISMA Extension for Scoping Reviews (PRISMA-ScR): Checklist and Explanation. *Ann Intern Med* 2018;169(7):467-73. doi: 10.7326/m18-0850 [published Online First: 2018/09/05]
20. Serrata Malfitano AP, Gomes da Mota de Souza R, Esquerdo Lopes R. Occupational justice and its related concepts: An historical and thematic scoping review. *OTJR: Occupation, participation and health* 2016;36(4):167-78.
21. Berry C, Greenwood K. Direct and indirect associations between dysfunctional attitudes, self-stigma, hopefulness and social inclusion in young people experiencing psychosis. *Schizophrenia Research* 2017 doi: 10.1016/j.schres.2017.06.037
22. Clewes J, Shivamurthy S, Wrigley S. Brightening His Days: A Reflective Single Case-Study of Social Integration and Recovery. *Occupational Therapy in Mental Health* 2013;29(2):159-80.
23. Farrell C, Bryant W. Voluntary work for adults with mental health problems: An exploration of the perspectives of recruiters. *The British Journal of Occupational Therapy* 2009;72(5):188-96. doi: 10.1177/030802260907200502
24. Fieldhouse J. Community participation and recovery for mental health service users: an action research inquiry. *British Journal of Occupational Therapy* 2012;75(9):419-28.
25. Fieldhouse J. Mental health, social inclusion, and community development: lessons from Bristol. *Community Development Journal* 2012;47(4):571-87. doi: 10.1093/cdj/bss028

For peer review only - http://bmjopen.bmj.com/site/about/guidelines.xhtml

26. Killaspy H, White S, Lalvani N, et al. The impact of psychosis on social inclusion and associated factors. *International Journal of Social Psychiatry* 2014;60(2):148-54. doi: 10.1177/0020764012471918
27. Mazzi F, Baccari F, Mungai F, et al. Effectiveness of a social inclusion program in people with non-affective psychosis. *BMC Psychiatry* 2018;18(1) doi: 10.1186/s12888-018-1728-5
28. Nieminen I, Ramon S, Dawson I, et al. Experiences of social inclusion and employment of mental health service users in a european union project. *International Journal of Mental Health* 2012;41(4):3-23. doi: 10.2753/IMH0020-7411410401
29. Raitakari S, Haahtela R, Juhila K. Tackling community integration in mental health home visit integration in Finland. *Health & Social Care in the Community* 2016;24(5):E53-E62. doi: 10.1111/hsc.12246
30. Ramon S, Griffiths CA, Nieminen I, et al. Towards social inclusion through lifelong learning in mental health: Analysis of change in the lives of the EMILIA project service users. *International Journal of Social Psychiatry* 2011;57(3):211-23. doi: 10.1177/0020764009354943
31. Saavedra J, Pérez E, Crawford P, et al. Recovery and creative practices in people with severe mental illness: evaluating well-being and social inclusion. *Disability and Rehabilitation* 2018;40(8):905-11. doi: 10.1080/09638288.2017.1278797
32. Smyth G, Harries P, Dorer G. Exploring mental health service users' experiences of social inclusion in their community occupations. *British Journal of Occupational Therapy* 2011;74(7):323-31. doi: 10.4276/030802211X13099513661072
33. Turner N, Ferguson L, Hill M, et al. An exploratory study of the extent of social inclusion among people with psychosis and psychotic-related conditions. *International Journal of Social Psychiatry* 2017;63(3):195-202. doi: 10.1177/0020764017691551
34. Hamer HP, Kidd J, Clarke S, et al. Citizens un-interrupted: Practices of inclusion by mental health service users. *Journal of Occupational Science* 2017;24(1):76-87. doi: 10.1080/14427591.2016.1253497
35. Stain HJ, Galletly CA, Clark S, et al. Understanding the social costs of psychosis: The experience of adults affected by psychosis identified within the second Australian national survey of psychosis. *Australian and New Zealand Journal of Psychiatry* 2012;46(9):879-89. doi: 10.1177/0004867412449060
36. Moraes Salles M, Barros Sn. THE SOCIAL EXCLUSION/INCLUSION OF USERS OF A PSYCHOSOCIAL CARE CENTER IN EVERYDAY LIFE. *Texto & Contexto Enfermagem* 2013;22(3):704-12.
37. Salles MM, Barros S. Social representation of users of a Psychosocial Care Center and those in their social network on mental illness and social inclusion. *Saude E Sociedade* 2013;22(4):1059-71. doi: 10.1590/s0104-12902013000400009
38. Harrison D, Sellers A. Occupation for mental health and social inclusion. *British Journal of Occupational Therapy* 2008;71(5):216-19.
39. Le Boutillier C, Croucher A. Social inclusion and mental health. *British Journal of Occupational Therapy* 2010;73(3):136-39.
40. Townsend EA. Boundaries and bridges to adult mental health: Critical occupational and capabilities perspectives of justice. *Journal of Occupational Science* 2012;19(1):8-24.
41. Mandiberg JM. Commentary: the failure of social inclusion: an alternative approach through community development. *Psychiatric Services* 2012;63(5):458-60.
42. Pettican A, Bryant W. Sustaining a focus on occupation in community mental health practice. *British Journal of Occupational Therapy* 2007;70(4):140-46.
43. Cáceres CF, Aggleton P, Galea JT. Sexual diversity, social inclusion and HIV/AIDS

AIDS 2008;22(Suppl2):S45-S55.
44. Cobigo V, Stuart H. Social inclusion and mental health. *Current Opinion in Psychiatry* 2010;23(5):453-57. doi: 10.1097/YCO.0b013e32833bb305
45. Evans J, Repper J. Employment, social inclusion and mental health. *Journal of Psychiatric and Mental Health Nursing* 2000;7(1):15-24. doi: 10.1046/j.1365-2850.2000.00260.x

For peer review only - http://bmjopen.bmj.com/site/about/guidelines.xhtml

46. Hamer H. Returning citizens: Social inclusion and occupational justice for mental health service users. *Cadernos Brasileiros de Saúde Mental/Brazilian Journal of Mental Health* 2017;9(21):89-113.
47. Rawal N. Social inclusion and exclusion: A review. *Dhaulagiri Journal of Sociology and Anthropology* 2008;2:161-80.
48. Silver H. The contexts of social inclusion. 2015.
49. Baumgartner JN, Burns JK. Measuring social inclusion—a key outcome in global mental health. *International journal of epidemiology* 2013;43(2):354-64.
50. O'Donnell P, O'Donovan D, Elmusharaf K. Measuring social exclusion in healthcare settings: a scoping review. *International journal for equity in health* 2018;17(1):15.
51. Huxley P, Thornicroft G. Social inclusion, social quality and mental illness. *The British Journal of Psychiatry* 2003;182(4):289-90.
52. Lloyd C, Tse S, Deane FP. Community participation and social inclusion: How practitioners can make a difference. *AeJAMH (Australian e-Journal for the Advancement of Mental Health)* 2006;5(3):1-10. doi: 10.5172/jamh.5.3.185
53. Martin L, Cobigo V. Definitions matter in understanding social inclusion. *Journal of Policy and Practice in Intellectual Disabilities* 2011;8(4):276-82.
54. Duffy M, Sharer M, Cornman H, et al. Integrating Mental Health and HIV Services in Zimbabwean Communities: A Nurse and Community-led Approach to Reach the Most Vulnerable. *Journal of the Association of Nurses in AIDS Care* 2015:1-13. doi: 10.1016/j.jana.2015.09.003
55. Huxley PJ, Chan K, Chiu M, et al. The social and community opportunities profile social inclusion measure: Structural equivalence and differential item functioning in community mental health residents in Hong Kong and the United Kingdom. *International Journal of Social Psychiatry* 2016;62(2):133-40.
56. Chan K, Evans S, Ng YL, et al. A Concept Mapping Study on Social Inclusion in Hong Kong. *Social Indicators Research* 2014;119(1):121-37. doi: 10.1007/s11205-013-0498-1

For peer review only

For peer review only - http://bmjopen.bmj.com/site/about/guidelines.xhtml

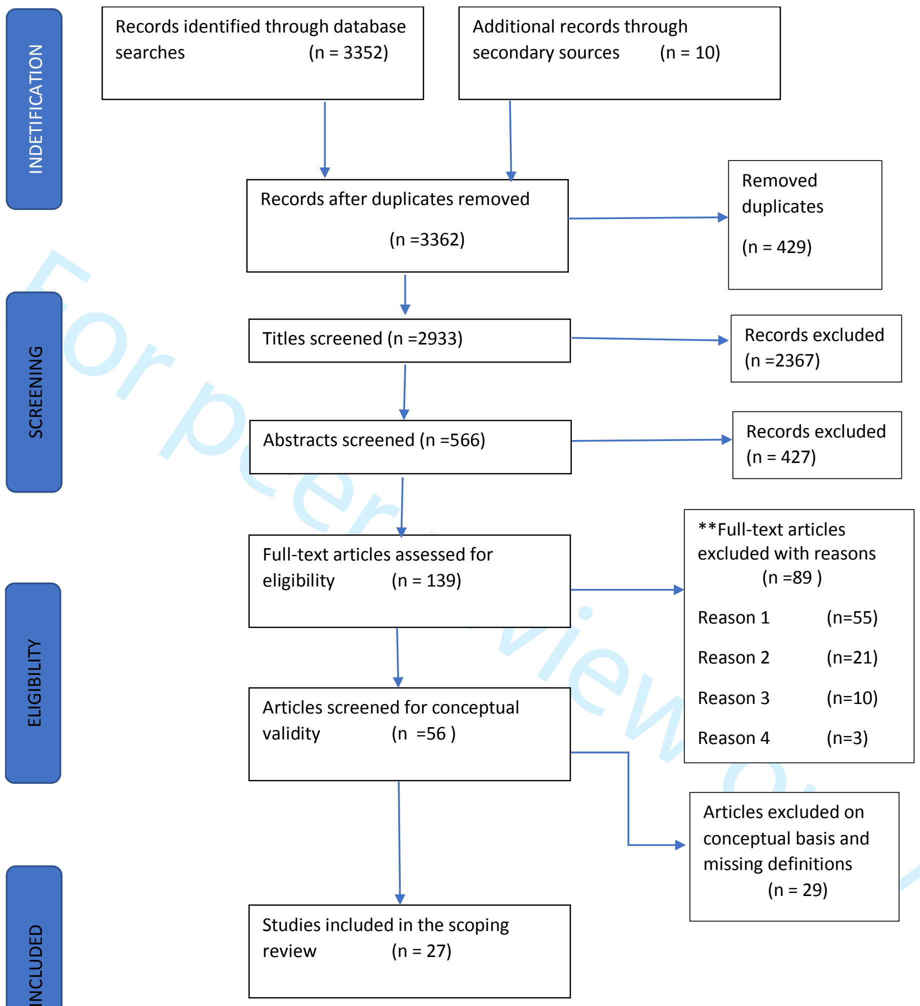

****Reasons for full-text articles exclusion**
(n =89)

Reason 1 (n=55): No explicit mention of either of the concepts or their related terms

Reason 2 (n=21): Not applied to target population groups, that is HIV and or mental illness

Reason 3 (n=10): No English version of full article

Reason 4 (n=3): Book reviews/chapters

Figure 1: Scoping review flow diagram

Figure 2: Commonalities between occupational justice and social inclusion definitions – associated terms

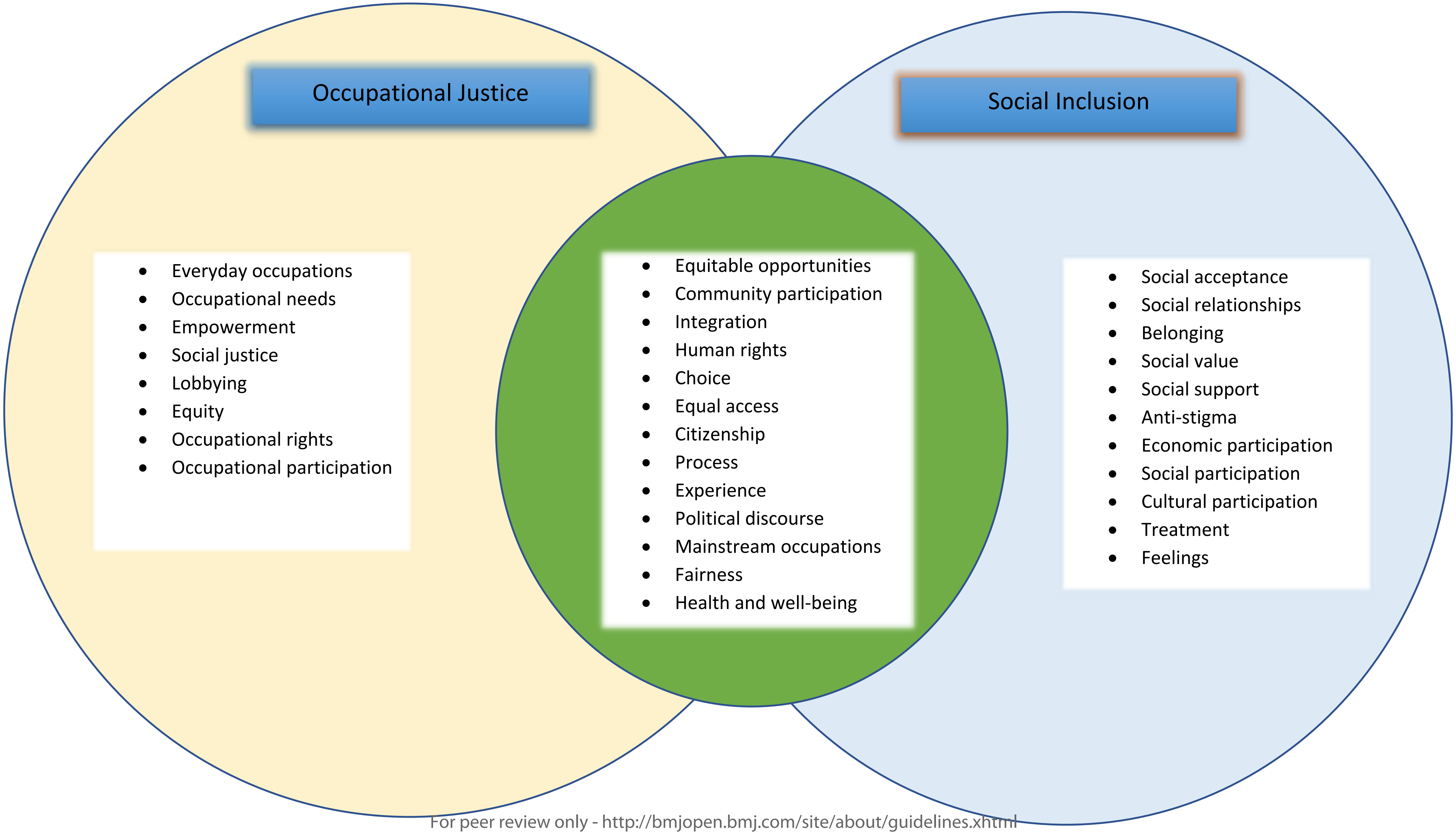

Appendix 1. PubMed Search Strategy

PubMed

Set 1

1. Social Justice [MeSH]

2. Social Isolation [MeSH]

3. Social Marginalization [MeSH]

4. Social Participation [MeSH]

5. Rehabilitation, Vocational [MeSH]

6. Injustice OR justice OR social inclusion OR social exclusion OR social isolation OR social separation OR social barriers OR social distance OR social acceptance OR social rejection OR social participation OR deprivation OR marginalization OR alienation

7. 1 OR 2 OR 3 OR 4 OR 5 OR 6 *(represents Social inclusion set)*

Set 2

1. Social stigma [MeSH]

2. Prejudice [MeSH]

3. Stigma OR prejudice OR stigmatise OR stigmatisation OR stigmatize OR stigmatization OR discrimination

4. 1 OR 2 OR 3 *(represents Stigma set)*

Set 3

1. Occupational justice OR Occupational injustice OR Occupational deprivation OR Occupational alienation OR Occupational marginalisation OR Occupational imbalance OR Occupational OR occupation OR occupations OR activities OR work OR employment OR unemployment OR engagement (*this set is used to narrow search to occupation as defined by OT not PubMed's definition)*

Set 4

1. Mental Disorders [MeSH] *(this heading includes substance-related disorders)*

2. Mentally Ill Persons [MeSH]

For peer review only

3. Mental disorders OR mental illness OR mentally ill OR Psychiatric disorder OR psychiatric illness OR psychological disorder OR Developmental Disability OR Intellectual Development Disorder OR Intellectual disability OR Mental retardation OR Mental deficiency
4. 1 OR 2 OR 3 *(represents Mental Disorders set)*

Set 5

1. HIV [MeSH]
2. HIV Infections [MeSH]
3. Acquired Immunodeficiency Syndrome (MeSH)
4. HIV OR human immune deficiency virus OR AIDS OR acquired immunodeficiency syndrome OR acquired immune deficiency syndrome OR HIV/AIDS
5. 1 OR 2 OR 3 OR 4

Now combine Sets, 1 AND 2 AND 3 AND 4 or Sets 1 AND 2 AND 3 AND 5
Limit to last 20 years

English

Date: 31/01/19

For peer review only

Preferred Reporting Items for Systematic reviews and Meta-Analyses extension for Scoping Reviews (PRISMA-ScR) Checklist

SECTION	ITEM	PRISMA-ScR CHECKLIST ITEM	REPORTED ON PAGE #
**TITLE**			
Title	1	Identify the report as a scoping review.	Page 1
**ABSTRACT**			
Structured summary	2	Provide a structured summary that includes (as applicable): background, objectives, eligibility criteria, sources of evidence, charting methods, results, and conclusions that relate to the review questions and objectives.	Page 2
**INTRODUCTION**			
Rationale	3	Describe the rationale for the review in the context of what is already known. Explain why the review questions/objectives lend themselves to a scoping review approach.	Page 5
Objectives	4	Provide an explicit statement of the questions and objectives being addressed with reference to their key elements (e.g., population or participants, concepts, and context) or other relevant key elements used to conceptualize the review questions and/or objectives.	Page 5
**METHODS**			
Protocol and registration	5	Indicate whether a review protocol exists; state if and where it can be accessed (e.g., a Web address); and if available, provide registration information, including the registration number.	Yes ref 15
Eligibility criteria	6	Specify characteristics of the sources of evidence used as eligibility criteria (e.g., years considered, language, and publication status), and provide a rationale.	Page 6
Information sources*	7	Describe all information sources in the search (e.g., databases with dates of coverage and contact with authors to identify additional sources), as well as the date the most recent search was executed.	Page 6
Search	8	Present the full electronic search strategy for at least 1 database, including any limits used, such that it could be repeated.	Page 6. Appendix 1
Selection of sources of evidence†	9	State the process for selecting sources of evidence (i.e., screening and eligibility) included in the scoping review.	Page 6
Data charting process‡	10	Describe the methods of charting data from the included sources of evidence (e.g., calibrated forms or forms that have been tested by the team before their use, and whether data charting was done independently or in duplicate) and any processes for obtaining and confirming data from investigators.	Page 8
Data items	11	List and define all variables for which data were sought and any assumptions and simplifications made.	Page 9. Table 1
Critical appraisal of individual sources of evidence§	12	If done, provide a rationale for conducting a critical appraisal of included sources of evidence; describe the methods used and how this information was used in any data synthesis (if appropriate).	Click here to enter text.

For peer review only

St. Michael's
Inspired Care. Inspiring Science.

For peer review only - http://bmjopen.bmj.com/site/about/guidelines.xhtml

SECTION	ITEM	PRISMA-ScR CHECKLIST ITEM	REPORTED ON PAGE #
Synthesis of results	13	Describe the methods of handling and summarizing the data that were charted.	Page 8
**RESULTS**			
Selection of sources of evidence	14	Give numbers of sources of evidence screened, assessed for eligibility, and included in the review, with reasons for exclusions at each stage, ideally using a flow diagram.	Page 8. Figure 1
Characteristics of sources of evidence	15	For each source of evidence, present characteristics for which data were charted and provide the citations.	Page 8-14. Table 1
Critical appraisal within sources of evidence	16	If done, present data on critical appraisal of included sources of evidence (see item 12).	Click here to enter text.
Results of individual sources of evidence	17	For each included source of evidence, present the relevant data that were charted that relate to the review questions and objectives.	Table 1
Synthesis of results	18	Summarize and/or present the charting results as they relate to the review questions and objectives.	Page 14-18
**DISCUSSION**			
Summary of evidence	19	Summarize the main results (including an overview of concepts, themes, and types of evidence available), link to the review questions and objectives, and consider the relevance to key groups.	Page 18-20
Limitations	20	Discuss the limitations of the scoping review process.	Page 2
Conclusions	21	Provide a general interpretation of the results with respect to the review questions and objectives, as well as potential implications and/or next steps.	Page 21
**FUNDING**			
Funding	22	Describe sources of funding for the included sources of evidence, as well as sources of funding for the scoping review. Describe the role of the funders of the scoping review.	Page 22

JBI = Joanna Briggs Institute; PRISMA-ScR = Preferred Reporting Items for Systematic reviews and Meta-Analyses extension for Scoping Reviews.
* Where *sources of evidence* (see second footnote) are compiled from, such as bibliographic databases, social media platforms, and Web sites.
† A more inclusive/heterogeneous term used to account for the different types of evidence or data sources (e.g., quantitative and/or qualitative research, expert opinion, and policy documents) that may be eligible in a scoping review as opposed to only studies. This is not to be confused with *information sources* (see first footnote).
‡ The frameworks by Arksey and O'Malley (6) and Levac and colleagues (7) and the JBI guidance (4, 5) refer to the process of data extraction in a scoping review as data charting.
§ The process of systematically examining research evidence to assess its validity, results, and relevance before using it to inform a decision. This term is used for items 12 and 19 instead of "risk of bias" (which is more applicable to systematic reviews of interventions) to include and acknowledge the various sources of evidence that may be used in a scoping review (e.g., quantitative and/or qualitative research, expert opinion, and policy document).

From: Tricco AC, Lillie E, Zarin W, O'Brien KK, Colquhoun H, Levac D, et al. PRISMA Extension for Scoping Reviews (PRISMAScR): Checklist and Explanation. Ann Intern Med. 2018;169:467–473. doi: 10.7326/M18-0850.

For peer review only - http://bmjopen.bmj.com/site/about/guidelines.xhtml

BMJ Open

Occupational justice and social inclusion amongst people living with HIV and people with mental illness: A scoping review

Journal:	*BMJ Open*
Manuscript ID	bmjopen-2020-036916.R1
Article Type:	Original research
Date Submitted by the Author:	16-Apr-2020
Complete List of Authors:	NHUNZVI, CLEMENT; University of Zimbabwe, College of Health Sciences, Rehabilitation Department<br>Langhaug, Lisa; African Mental Health Research Initiative (AMARI) , Department of Psychiatry, College of Health Sciences. University of Zimbabwe.<br>Mavindidze, Edwin; Ingutsheni Central Hospital, Occupational Therapy<br>Harding, Richard; King's College London, Florence Nightingale Faculty of Nursing Midwifery and Palliative Care, Cicely Saunders Institute.<br>Galvaan, Roshan ; University of Cape Town, Health and Rehabilitation Sciences
**Primary Subject Heading**:	Mental health
Secondary Subject Heading:	HIV/AIDS, Mental health, Public health, Rehabilitation medicine
Keywords:	MENTAL HEALTH, HIV & AIDS < INFECTIOUS DISEASES, REHABILITATION MEDICINE, PUBLIC HEALTH

SCHOLARONE™
Manuscripts

I, the Submitting Author has the right to grant and does grant on behalf of all authors of the Work (as defined in the below author licence), an exclusive licence and/or a non-exclusive licence for contributions from authors who are: i) UK Crown employees; ii) where BMJ has agreed a CC-BY licence shall apply, and/or iii) in accordance with the terms applicable for US Federal Government officers or employees acting as part of their official duties; on a worldwide, perpetual, irrevocable, royalty-free basis to BMJ Publishing Group Ltd ("BMJ") its licensees and where the relevant Journal is co-owned by BMJ to the co-owners of the Journal, to publish the Work in this journal and any other BMJ products and to exploit all rights, as set out in our licence.

The Submitting Author accepts and understands that any supply made under these terms is made by BMJ to the Submitting Author unless you are acting as an employee on behalf of your employer or a postgraduate student of an affiliated institution which is paying any applicable article publishing charge ("APC") for Open Access articles. Where the Submitting Author wishes to make the Work available on an Open Access basis (and intends to pay the relevant APC), the terms of reuse of such Open Access shall be governed by a Creative Commons licence – details of these licences and which Creative Commons licence will apply to this Work are set out in our licence referred to above.

Other than as permitted in any relevant BMJ Author's Self Archiving Policies, I confirm this Work has not been accepted for publication elsewhere, is not being considered for publication elsewhere and does not duplicate material already published. I confirm all authors consent to publication of this Work and authorise the granting of this licence.

For peer review only

Title: Occupational justice and social inclusion amongst people living with HIV and people with mental illness: A scoping review.

*Clement Nhunzvi[1,2,3]; Lisa Langhaug[3]; Edwin Mavindidze[4]; Richard Harding[5] & Roshan Galvaan[2]

* **Corresponding author: Clement Nhunzvi**

Department of Rehabilitation
University of Zimbabwe, College of Health Sciences
P.O. Box A178
Avondale, Harare
Zimbabwe
clemynhu@gmail.com; nhncle001@myuct.ac.za
TEL: +263 4 797800 Cell: +263 775 814 587

Affiliations

[1] Department of Rehabilitation, College of Health Sciences. University of Zimbabwe. Zimbabwe.

[2] Division of Occupational Therapy, Department of Health and Rehabilitation Sciences, Faculty of Health Sciences, University of Cape Town. South Africa.

[3] African Mental Health Research Initiative (AMARI), Department of Psychiatry, College of Health Sciences. University of Zimbabwe. Zimbabwe.

[4] Occupational Therapy Department, Ingutsheni Central Hospital, Bulawayo. Zimbabwe.

[5] Florence Nightingale Faculty of Nursing Midwifery and Palliative Care, Kings College London, Cicely Saunders Institute. UK.

Keywords: social inclusion, occupational justice, scoping review, HIV, mental illness

Word count: 4587

For peer review only

Abstract

Objective To explore ways in which occupational justice and social inclusion are conceptualised, defined, and operationalised in highly stigmatised and chronic conditions of mental illness and HIV.

Design This scoping review protocol followed Arksey and O`Malley`s (2005) Scoping Review Framework.

Data sources and eligibility. The following databases were searched for the period January 1997 – January 2019: Medline via PubMed, Scopus, Academic Search Premier, Cumulative Index to Nursing and Allied Health Literature (CINAHL), Africa-Wide Information, Humanities International Complete, Web of Science, PsychInfo, SocINDEX, and grey literature.

Eligible articles were primary studies, reviews or theoretical papers which conceptualised, defined and/or operationalised social inclusion or occupational justice in mental illness or HIV.

Study appraisal and synthesis. We undertook a three-part article screening process. Screening and data extraction were undertaken independently by two researchers. Arksey's framework and thematic analysis informed the collation and synthesis of included papers.

Results. From 3352 records, we reviewed 139 full articles and retained 27 for this scoping review. Definitions of social inclusion and occupational justice in the domains of mental illness and HIV were heterogeneous and lacked definitional clarity. The two concepts were conceptualised as either processes or personal experiences, with key features of community participation, respect for human rights, and establishment and maintenance of healthy relationships. Conceptual commonalities between social inclusion and occupational justice were premised on social justice.

Conclusions To address lack of clarity, we propose further and concurrent exploration of these concepts, specifically with reference to persons with comorbid mental health disorders such as substance use disorders and HIV living in low-income countries. This should reflect contextual realities influencing community participation, respect for human rights, and meaningful occupational participation. From this broadened understanding, quantitative measures should be applied to improve the standardization of measurements for occupational justice and social inclusion in policy, research and practice.

For peer review only - http://bmjopen.bmj.com/site/about/guidelines.xhtml

Title: Occupational justice and social inclusion among people living with HIV and people with mental illness: A scoping review.

Article Summary

Strengths and limitations of this study

- The methodology as provided by the scoping review design, facilitated comprehensive mapping of the literature, and presented a foundation for further exploration utilisation of the concepts to inform policy, research and practice.
- We used a rigorous strategy to explore research foci, definitions and utilisations of the concepts of social inclusion and occupational justice in mental illness and HIV.
- Data synthesis was limited to work published in English originally or with available English translated copies.
- We focused on mental illness, which is made up of several different conditions and could have introduced generalisation bias. However, most of the included studies were also not condition specific, fitting our primary aim for conceptual review.
- Focus was limited to conceptual and theoretical aspects of the concepts more than interventions and outcomes of interventions.

For peer review only

For peer review only - http://bmjopen.bmj.com/site/about/guidelines.xhtml

Introduction -

The global burden of disease from HIV remains substantially high with approximately 37.9 million people living with HIV[1] However, in the last two decades, the world has seen a combination of a significantly decreased mortality and a small decrease in incidence leading to an increase in the number of people living with HIV from 8·74 million (1990) to 36·9 million (2017).[2]This increase in the number of people living with HIV and decreased mortality rates are largely a result of the scaling up of HIV treatments.[1 3] However, what remains as a concern are persisting gaps in the treatment continuum towards the UNAIDS 90-90-90 target. Among those living with HIV who knew their status globally, 17% were still not on life-saving antiretroviral therapy UNAIDS Global AIDS update 2019.[1] Moreover, only 53% of those on treatment were virally suppressed. [1 3] One reason for these discrepancies, mainly seen in key populations, is the rising and ever complex relationship between mental illness and HIV.[4 5] Secondary to both biological and psychosocial factors, people living with HIV are at an increased risk of experiencing poor mental health [6 7] which negatively impacts on their health-seeking behaviours, adherence to antiretroviral treatments [8] and quality of life.[9] The prevalence of common mental disorders is also significantly higher among people living with HIV, irrespective of their being on ART, and is further impounded by stigma.[10] There is also a known bidirectional relationship between HIV and mental health, worsened by associated health and social inequalities. This often leaves people with severe mental illnesses at an increased risk for HIV infection.[11 12]

Individuals with chronic and usually stigmatised conditions such as mental illnesses, physical disabilities and HIV, face barriers to full participation in their communities.[13] Poverty, lack of access to education, lack of suitable housing, and unemployment are some of the social and economic barriers to accessing adequate and sustained healthcare faced by this group.[14] These barriers may similarly be experienced by people who face discrimination based on their class, race or gender identity or sexuality and thus, when persons with mental illness also share these characteristics they may be severely stigmatised or discriminated against.[15 16]

To sustain the aforementioned progress in the management of people with HIV and its comorbidities such as mental illness, there is a renewed call to take a community-led, equality and social justice approach[3 12] with concepts like occupational justice and social inclusion holding promise to inform this agenda. Occupational justice is an advanced form of social justice, concerned with equity and fairness for individuals, groups and communities access to

For peer review only

For peer review only - http://bmjopen.bmj.com/site/about/guidelines.xhtml

resources and opportunities that supports their engagement in diverse, healthy, and meaningful occupations.[17 18] On the other hand, social inclusion entails multi-dimensional processes or states where prevailing contextual conditions enable full and active participation in all aspects of everyday life.[19 20] This can include civic, social, economic, and political activities, as well as participation in decision making processes irrespective of personal characteristic differences.[19 20]

Social inclusion and occupational justice form aspects of social justice are therefore relevant to direct research and practice as we address exclusions and injustices experienced by stigmatised groups[21]. Focus on these social justice outcomes ensures that health and social well-being are addressed beyond the clinical management of the disease. Health-related quality of life that includes social inclusion and occupational justice will be a holistic construct for promoting continuum of care and health and well-being beyond viral suppression in HIV. [22 23] These concepts direct the health and social care communities to view individuals with mental illness and HIV as being part of marginalized groups at risk of being deprived of respect, rights and opportunities to achieve optimal health-related quality of life.

Social inclusion and occupational justice are potentially key concepts that can inform the promotion of human-rights based, sustainable, person and community-centred interventions that promote recovery for persons with chronic, and stigmatised conditions.[24 25] In order to aid integration, and operationalisation of occupational justice and social inclusion in practice, we need to understand how the concepts are conceptualised and applied in population groups affected by chronic and stigmatised conditions. Synthesised summaries of research evidence can inform primary research and implementation science,[26] therefore we selected a scoping review design to help advance this field.[27] This was a particularly appropriate method for this area due to the diverse disciplinary locations of the existing literature.[28 29] This scoping review aimed to explore and appraise the definitions, current utilisation, and relationships between the concepts of social inclusion and occupational justice in mental illness and HIV literature.

Methods

This scoping review followed our published study protocol[27], developed using Arksey and O'Malley's Scoping Review framework,[29] as well as guidelines for scoping review protocols in occupational therapy.[26 30] In this paper, a scoping review is taken to be a form of knowledge synthesis that addresses an exploratory research question rather than the highly focused question in a systematic review. [28 29] For reporting, we followed the Preferred Reporting Items

For peer review only - http://bmjopen.bmj.com/site/about/guidelines.xhtml

for Systematic reviews and Meta-Analyses extension for Scoping Reviews (PRISMA-ScR) Checklist. [31]

We followed an iterative process to develop and refine the research question.[29] Based on the subject area terminology, literature, and our understanding of current practice trends in managing conditions that are chronic, and stigmatised, we asked the following question:

How are occupational justice and social inclusion conceptualised, defined, and operationalised, and how are these concepts related in the highly stigmatised chronic conditions of mental illness and HIV?

The objectives of our scoping review were:

- To identify articles that define or conceptualise occupational justice and social inclusion related to mental illness or HIV.
- To describe how these are operationalised or utilised.
- To identify and describe relationships between occupational justice and social inclusion.
- To determine potential areas for further development, integration, and application of these concepts.

Search Strategy

With the aid of a subject librarian, we identified appropriate databases using a journal indexing system. We searched twelve databases in January2019: i) PubMed; ii) Scopus; iii) Academic Search Premier; iv) the Cumulative Index to Nursing and Allied Health Literature (CINAHL); v) Africa-Wide Information; vi) Humanities International Complete; vii) Web of Science; viii) PsychInfo; ix) SocINDEX; x) Grey Literature Report; xi) Web of Science Conference Proceedings; and, xii) Open Grey. We used PubMed as the free platform for accessing articles indexed on Medline database. The selected databases captured a comprehensive sample of literature from a variety of disciplines including social work, psychiatry, nursing and occupational therapy.

The first and last authors (CN & RG) worked with the librarian, through an iterative process, to develop an inclusive list of search terms and applicable filtering methods including Boolean phrases and MESH terms for each database.[27] We developed a general search strategy with primary search terms related to the primary concepts of occupational justice and social

For peer review only - http://bmjopen.bmj.com/site/about/guidelines.xhtml

inclusion while secondary search terms encompassed the broader terms of mental health, occupational therapy, mental illness, HIV, and rehabilitation (Table 1). For the purposes of search strategy development, we restricted our search to literature published between January 1997 and January 2019, a period which has seen the emergence and rapid growth in literature on occupational justice.[32] We conducted a preliminary search on PubMed and this enabled refinement of our search strategy to maximise sensitivity and specificity. We adapted the PubMed search strategy (Appendix 1 – provided as an online supplementary appendix) accordingly for other databases.

Table 1: General Search Strategy

Key Word	Alternative words
**Occupational therapy**	Occupational rehabilitation
**Mental health**	Mental illness OR Mental disorder OR Psychiatric disability
**Occupational justice**	Occupational injustice OR Occupational marginalisation OR Occupational alienation OR Occupational imbalance OR Occupational deprivation
**Social inclusion**	Social exclusion OR Social isolation OR social integration
**HIV**	HIV OR HIV/AIDS OR HIV infection OR AIDS

Study selection

The first author (CN) reviewed the titles identified in the search for eligibility. The aim was to identify articles that i) indicated a research focus on mental illness, or HIV, or both and ii) titles that included the key terms of occupational justice, social inclusion or both. Article types included primary studies, reviews, opinion papers and other theoretical papers without primary data. Articles were not eliminated where there was uncertainty with the title until it was examined more in-depth by looking at the abstract. Two independent reviewers, the first and third authors (CN & EM), reviewed titles and abstracts of preliminarily selected articles using predetermined inclusion and exclusion criteria (Table 2), detailed in our protocol.[27] These same reviewers each further screened full-text articles to determine if they met the inclusion/exclusion criteria. At this stage, articles were included if their explicit focus was on social inclusion or occupational justice in mental illness and or HIV; concepts were defined and reported some operationalisation of the two concepts. Discrepancies were resolved by consensus or by seeking adjudication from the second author (LL). The Cohen's κ statistic to

For peer review only - http://bmjopen.bmj.com/site/about/guidelines.xhtml

determine inter-rater agreement was calculated for the title and abstract review and the full article review stage, giving more than 90% agreement between reviewers with Cohen's κ statistic of 0.78 and 0.83 respectively.

Table 2: Inclusion and exclusion criteria for acceptable articles

**Criteria for inclusion**	**Criteria for exclusion**
Minimum criteria required in the abstract:<br>• Explicit mention of mental illness, and or HIV/AIDS.<br>• Explicit mention of either occupational justice and its varieties or social inclusion and its varieties. (varieties were informed by how articles with these terms were indexed in databases)<br>• Date range (January 1997 - January 2019).<br>• English language<br>• English translation of abstract and article available	• Used animal subjects.

Data Extraction

Guided by the study objectives, a common extraction table was designed, to extract standard bibliometric information study characteristic and main findings. The first five articles were reviewed by both the first and third authors, with the remaining articles divided between the two authors. We then checked for accuracy and completeness against each other's work. Discrepancies were resolved by revisiting the article, discussing, and reaching consensus.

review only

Data Synthesis

Data were synthesised descriptively to give a structured summary of the dataset and to capture the characteristics of the studies included and the definitional range of social inclusion and occupational justice. Study grouping followed the publication trends over time and study designs used. Descriptive statistics were calculated using Microsoft Excel 2016 for frequencies. We used deductive thematic analysis to organise the extracted definitions and related concepts for occupational justice and social inclusion.

Patient and public involvement:

Patient and public involvement (PPI) representatives were not directly involved in the design of this scoping review protocol. However, experiences of the first author in working with adults afflicted with HIV and mental health disorders in Zimbabwe informed the need to explore issues faced by this population beyond biomedical care. We also built our research question from insights being generated in his doctoral studies exploring occupational perspectives on social inclusion among young adults dually afflicted with substance use disorders and HIV. Social inclusion speaks to life beyond medical management which was not being given sufficient attention and hence the need to conduct a scoping review.

Results

Retained studies' characteristics

As described in Figure 1, we screened (n=3352) titles and after reviewing (n=139) full articles, (n=27) were included in this scoping review. Of the (n=27) sources included for final review, (n=23) were published between 2009-2018, with the majority of these (n=6) published in 2012 (Table 3). Most publications were by authors in the mental health field and from high income countries, with 68% of the primary studies being conducted in Europe[33-45], and 9% in Australia.[46 47] No primary studies were conducted in North America, Africa, or Asia. More than a third of the studies (n=10) utilised a qualitative research design[34-37 40-42 44 46 48 49], five (16%) utilised a quantitative research design[33 38 39 45 47], and only one study utilised a mixed methods design.[43] Six (19%) were review papers and the remainder (n=5) were opinion[50 51], lectureship[52], commentary[53] and theoretical analysis papers.[54] The two concepts were predominantly explored around mental illness (n=26) with significantly less focus on HIV (n=1). The majority (n=21) of the published research investigated social inclusion as it related to mental health conditions, and all the occupational justice papers were focussed on mental

For peer review only - http://bmjopen.bmj.com/site/about/guidelines.xhtml

illness. Only one paper was included which discussed social inclusion in relation to people living with HIV.[55] We found no published literature that explored occupational justice and social inclusion in populations with comorbid mental illness and HIV.

Insert Figure 1: **Scoping review flow diagram

For peer review only

For peer review only - http://bmjopen.bmj.com/site/about/guidelines.xhtml

Table 3: Summary of articles defining social inclusion and occupational justice in mental illness and HIV						
**Author(s) Year**	**Participants**<br><br>**Setting**	**Design**	**Aim**	**Definition(s) of social inclusion and or occupational justice**	**How operationalized**	**Associated key terms used**
3a: Studies with primary data collection						
Mazzi *et al.*<br><br>2018	30 people with non-affective psychosis. In a social inclusion intervention program<br><br>Italy	Cross-sectional study	"To assess whether a social inclusion intervention is associated with better outcomes in terms of personal and social recovery, with particular reference to the areas of social functioning and activity, and subjective dimensions such as self-esteem, self-stigma and perceived quality of life" (p.1)	Social inclusion is the opportunity for an individual to participate in key functions or activities and in the economic, social and cultural life of his/her community, exercising the rights of his/her citizenship and enjoying an adequate standard of living and wellbeing.	As an outcome and intervention<br>As a human rights issue	social withdrawal, quality of life, discrimination, social recovery, social inclusion activities, social disadvantage
Saavedra *et al.*<br><br>2018	31 service users with severe mental illness. In workshops for social integration<br><br>Spain	Mixed methods study	To evaluate the impact of an artistic workshop on a group of people diagnosed/screened for with severe mental illness with focus on the impact of creative practices on well-being and social inclusion outcomes.	Social inclusion stated as a personal construct measured through perception of social isolation, social relationship and social acceptability	As an outcome to be evaluated based on personal perception<br><br>Measured using Social Inclusion Questionnaire	stigma, wellbeing, recovery, social isolation, social relationship, social acceptability

For peer review only - http://bmjopen.bmj.com/site/about/guidelines.xhtml

Table 3: Summary of articles defining social inclusion and occupational justice in mental illness and HIV

**Author(s) Year**	**Participants**<br><br>**Setting**	**Design**	**Aim**	**Definition(s) of social inclusion and or occupational justice**	**How operationalized**	**Associated key terms used**
Berry & Greenwood<br><br>2017	51 young outpatient service users with first episode psychosis. In Early Intervention in Psychosis, Community Mental Health and Assertive Outreach services<br><br>United Kingdom	Longitud-inal study	"To investigate the direct and indirect associations between dysfunctional attitudes, self-stigma, hopefulness, social inclusion and vocational activity for young people with psychosis" (p.197).	Social inclusion comprises social activity and community belonging.	As measured by levels of social activity and community belonging using Social Relationships Scale and Social Inclusion Scale	self-stigma, hopefulness, social activity, community belonging, social network, social contact, vocational activity
Turner *et al.*<br><br>2017	71 people with psychotic-related conditions. In a research programme on schizophrenia<br><br>Ireland	Quantita-tive descriptive study	To explore "the level of social inclusion among people with psychotic-related conditions using a standardised interview" (p.195)	Social inclusion is a multi-dimensional phenomenon with a number of domains including socially valued role functioning, social support, absence of stigma, and integration in rehabilitation community and wider community	As a multi-dimensional phenomenon<br>As both objective and subjective outcome<br>Assessed using Social Inclusion Interview Schedule	supportive relationships, stigma, integration, social exclusion, social support, rehabilitation.

For peer review only - http://bmjopen.bmj.com/site/about/guidelines.xhtml

Table 3: Summary of articles defining social inclusion and occupational justice in mental illness and HIV						
**Author(s) Year**	**Participants**<br><br>**Setting**	**Design**	**Aim**	**Definition(s) of social inclusion and or occupational justice**	**How operationalized**	**Associated key terms used**
Raitakari *et al.*<br><br>2016	16 mental health service users. In two mental health floating support services (FSS)<br><br>Finland	Qualitative study	To explore how community integration is understood and tackled in mental health floating support services (FSSs) and, more precisely, in service user–practitioner home visit interaction	Social inclusion is articulated in political discourse as a way to tackle social exclusion and to increase citizens' participation and activity in society	As supported integration in community life.<br><br>As participation in what marginalised individuals are usually excluded from	social exclusion, citizenship, community integration, housing, interaction, mental health, recovery
Killapsy *et al.*<br><br>2014	67 mental health service users with psychosis. Living in the boroughs of London and the inner-city<br><br>United Kingdom	Quantita-tive study	To investigate change in social inclusion after the development of a psychotic illness (clinically diagnosed) and associated factors	"Social inclusion refers to the opportunities that individuals have to participate in key areas of economic, social and cultural life" (p.148)	Focus on participation and access to services and opportunities<br><br>Five domains of social inclusion: social integration, consumption, access to services, productivity, political engagement<br>Assessed using the Social Inclusion Questionnaire User Experience	social exclusion, social disadvantage, social integration, employment, social isolation

For peer review only

Table 3: Summary of articles defining social inclusion and occupational justice in mental illness and HIV						
**Author(s) Year**	**Participants**<br><br>**Setting**	**Design**	**Aim**	**Definition(s) of social inclusion and or occupational justice**	**How operationalized**	**Associated key terms used**
Salles & Barros<br><br>2013a	17 mental health service users and 12 individuals from their social networks. In Psychosocial Care Centres<br><br>Brazil	Qualitative study	"To identify and analyse the conceptions expressed by the interviewees about social inclusion and mental illness" (p.37)	"Social inclusion is a process of promoting rights, access, choice and participation. For individuals with mental health problems, this also means access to the best possible forms of treatment" (p.37)	As a dynamic, multi-dimensional process.	work participation poverty
Salles & Barros<br><br>2013b	17 mental health service users and 12 individuals from their social networks. In Psychosocial Care Centres<br><br>Brazil	Qualitative study	"To identify the daily life experiences of users of a psychosocial care center related to processes of social exclusion and inclusion" (p.704).	Social inclusion is a process of the individual's choice on how to live her daily life" (p.710) and not just about experiencing full participation in society.	As a multidimensional process<br>As a human rights issue<br>As subjective experience centred on work participation	social exclusion, discrimination, prejudice, stigma, social isolation, social participation, empowerment, poverty

For peer review only

Table 3: Summary of articles defining social inclusion and occupational justice in mental illness and HIV						
**Author(s) Year**	**Participants**<br><br>**Setting**	**Design**	**Aim**	**Definition(s) of social inclusion and or occupational justice**	**How operationalized**	**Associated key terms used**
Clewes *et al.*<br><br>2013	Single Case-study with bipolar depression. In occupational therapy interventions under UK National Health Services (NHS)<br><br>United Kingdom	Case study	To illustrate how the combination of medical outpatient clinic and occupational therapy intervention together made a big difference in a person's life.	Social inclusion was taken as meaningful inclusion in the areas of life where the participant wished to be.	As personal experience.<br><br>As a degree of autonomy, ownership, responsibility, and independence.	stigma, recovery, social policy, empowerment, rights, engagement, spirituality, client leading.
Nieminen *et al.*<br><br>2012	23 mental health service users. In an intervention group follow up study<br><br>The European Union	Qualitative study	To describe how the mental health service users experienced social inclusion and employment in the European Union EMILIA project	Social inclusion is a subjective sense of belonging and active citizenship that enhances social integration	As an experience and feeling of active citizenship	empowerment, social network, finance and housing employment. social exclusion. stigma, prejudice
Fieldhouse<br><br>2012	Eight mental health service users in a two-year action research project<br><br>United Kingdom	Qualitative research	To describe an action research project that explored the recovery journeys of a group of assertive outreach service users who had progressed from being socially excluded and occupationally deprived to being participants in their local communities and to use this knowledge to inform local service development	Social inclusion is when people with mental health challenges enjoy rewarding social relations through renewed engagement in mainstream occupations resulting in fuller community participation.	As active participation in context or local community<br><br>As an outcome of occupation-based interventions.	social participation, social relationships, stigma, social exclusion, community participation, belonging, social capital

For peer review only - http://bmjopen.bmj.com/site/about/guidelines.xhtml

Table 3: Summary of articles defining social inclusion and occupational justice in mental illness and HIV						
**Author(s) Year**	**Participants**<br><br>**Setting**	**Design**	**Aim**	**Definition(s) of social inclusion and or occupational justice**	**How operationalized**	**Associated key terms used**
Stain *et al.*<br><br>2012	1825 adults with psychosis in an Australian national survey of psychosis.<br><br>Australia	Quantitative study	"To explore the impact of psychosis on an individual's social and community participation" (p. 879)	"Social inclusion refers to the participation of a person in society and is evidenced by an individual having the opportunities, resources and abilities to build and maintain relationships, engage in education and employment, and participate in community events and organisations" (p.880)	As participation in mainstream life as empowered individuals	social isolation, social anxiety, stigma, social participation, community participation
Smyth *et al.*<br><br>2011	Eight mental health service users in mental health rehabilitation services in inner-city area<br><br>United Kingdom	Qualitative study	To explore the experiences of social inclusion for mental health service users and factors associated when engaging in everyday community occupations	Social inclusion is developing fair access to opportunity in key social and economic spheres for marginalised groups.	As an issue of participation and access to services and opportunities	stigma occupational deprivation, discrimination, social networks
Ramon *et al.*<br><br>2011	27 key informants in an evaluation study of the EMILIA project<br><br>The European Union	Qualitative study	To identify how participation in the EMILIA project affects the lives of mental health service users in relation to social inclusion, employment and recovery	Social inclusion is a subjective sense of belonging and active citizenship that enhances social integration	As an experience and feeling about participation in society	employment, well-being, quality of life, social networks.

For peer review only - http://bmjopen.bmj.com/site/about/guidelines.xhtml

Table 3: Summary of articles defining social inclusion and occupational justice in mental illness and HIV

**Author(s) Year**	**Participants**<br><br>**Setting**	**Design**	**Aim**	**Definition(s) of social inclusion and or occupational justice**	**How operationalized**	**Associated key terms used**
Hamer *et al.*<br><br>2017	82 mental health service users<br><br>New Zealand & Brazil	Qualitative study	To present service users' stories of distressing exclusion that interrupted their rights to occupational justice, and marginalised them from occupation.<br>The paper also presents the practices of inclusion that service users engaged in that restored their rights and responsibilities as occupied and active citizens	Social inclusion is the extent to which people are confident about and able to exercise their rights and participate, by choice, in the ordinary activities of citizens<br><br>Occupational justice recognises the person's right to inclusive participation in everyday occupations regardless of age, ability, gender, social class, or other differences.	Social inclusion as a policy issue<br>As a dynamic concept, derived from subjective experiences<br><br>Social inclusion is enhanced through occupational justice<br><br>As inclusive participation<br>As a human right issue	occupational injustice, citizenship, stigma, discrimination
Fieldhouse<br><br>2012	Eight mental health service users in a two-year action research project<br><br>United Kingdom	Qualitative Study	To examine the impact of community participation on their recovery and social inclusion and how service users' experiences informed joint planning between mental health services and the learning community to promote social inclusion.	Social inclusion as the process of enabling citizenship through fuller community participation.<br><br>Occupational justice defined as the process of lobbying for the occupational needs of individuals and communities as part of a fair, inclusive, and empowering society; as a community reintegration issue.	As active participation in mainstream society	social participation, social relationships, stigma, social exclusion, community participation, belonging

For peer review only - http://bmjopen.bmj.com/site/about/guidelines.xhtml

Table 3: Summary of articles defining social inclusion and occupational justice in mental illness and HIV						
**Author(s) Year**	**Participants**<br><br>**Setting**	**Design**	**Aim**	**Definition(s) of social inclusion and or occupational justice**	**How operationalized**	**Associated key terms used**
Farrell & Bryant<br><br>2009	Nine recruiters of volunteers who had mental illness.<br><br>United Kingdom	Qualitative study	To explore the recruiters' understanding of mental health problems, drawing on their experiences	Occupational justice is an intrinsic part of social justice, permitting equitable opportunity and the means to choose, organise and perform meaningful occupations<br><br>Social inclusion stated but not defined	As a process and an outcome<br><br>Occupational justice as an intrinsic aspect of social inclusion	volunteering, discrimination, stigmatization, social exclusion, social attitudes, prejudice, occupational deprivation, occupational marginalisation, occupational apartheid
3b: Review, commentary, lectureship and opinion papers						
Le Boutillier & Croucher<br><br>2010	Mental health service users	Opinion paper	To present an alternative to the polarised view of social inclusion	Social inclusion is a multidimensional virtuous circle aimed at improving rights of access to the social and economic world, new opportunities, recovery of social identity and meaningful life and also reduced impact of disability on everyday life.	As a multidimensional system	Social exclusion, occupational justice, occupational balance, occupational alienation, occupational deprivation

For peer review only - http://bmjopen.bmj.com/site/about/guidelines.xhtml

Table 3: Summary of articles defining social inclusion and occupational justice in mental illness and HIV

**Author(s) Year**	**Participants**<br><br>**Setting**	**Design**	**Aim**	**Definition(s) of social inclusion and or occupational justice**	**How operationalized**	**Associated key terms used**
Cobigo & Stuart<br><br>2010	Mental health service users	Review	To review recent research on approaches to improving social inclusion for people with mental disabilities	Social inclusion is when one feels accepted and recognized as an individual beyond the disability; has positive personal relationships with family, friends, and acquaintances; is actively involved in recreation, leisure, and other social activities; has appropriate living accommodations; has healthy employment;<br>and has appropriate formal (service system) and informal (family and caregiver) supports.	As an acceptance, human rights, outcome of interventions issue	stigma, discrimination, legislation, community support, disability rights, justice, human rights
Caceres *et al.*<br><br>2008	Global literature on MSM with HIV	Review	To analyse reasons for continued risk of HIV and its consequences in MSM globally	"a social inclusion perspective on HIV prevention and AIDS care implies the adoption of a broad range of strategies to understand and confront social vulnerability" (p.11)	As a perspective to addressing vulnerability	social exclusion _ describes the alienation or disenfranchisement that certain individuals or groups experience within society.<br>stigma discrimination, prejudice, human rights, poverty, migration, employment participation, sexuality
Lloyd *et al.*<br><br>2006	Mental health service users	Review	To describe a selected number of activities that promote social inclusion.	Social inclusion involves being able to re-join or participate in leisure, friendship and work communities.	Participating and accessing services and opportunities	connectedness and interdependence

For peer review only - http://bmjopen.bmj.com/site/about/guidelines.xhtml

Table 3: Summary of articles defining social inclusion and occupational justice in mental illness and HIV						
**Author(s) Year**	**Participants<br><br>Setting**	**Design**	**Aim**	**Definition(s) of social inclusion and or occupational justice**	**How operationalized**	**Associated key terms used**
Farone<br><br>2006	Mental health service users, with a focus on schizophrenia	Review	To examine empirical evidence describing experiences with social or community integration for people with psychiatric disabilities, with a particular interest in schizophrenia	Social inclusion discussed but not defined	A link to mental and emotional well-being	community integration, community inclusion, social integration, stigma, social support, social networks
Evans & Repper<br><br>2000	Mental health service users	Review	To challenge common misconceptions surrounding employment, work, and mental health problems of mental health service users.	Social inclusion is defined as a need, aspiration, and citizenship issue among mental health service users. Social inclusion as an outcome of work participation.	A social need. An outcome of work participation	social exclusion, stigma, unemployment, poverty
Mandiberg 2012	People with psychiatric disabilities	Commentary	To describe the failure of social inclusion as a concept and present an alternative approach through community development	Social inclusion refers to full participation in the broader community for people with severe mental illnesses	As experience of participation in the broader community	work integration, community development, social enterprises
Townsend<br><br>2012	Mental health service users<br><br>Canada	Lectureship	To propose for an interdisciplinary knowledge exchange with a critical occupational perspective on the question: What lessons on boundaries and bridges to adult mental health can be drawn by connecting the capabilities and occupational frameworks of justice?	Occupational justice as the enjoyment of the 'occupational rights' of all people to engage and be socially included in their desired occupations, and thereby to contribute positively to their own well-being and the well-being of their communities.	As a human rights, capabilities and justice issue	occupational rights, occupational possibilities occupational deprivation, alienation, imbalance and marginalisation

For peer review only - http://bmjopen.bmj.com/site/about/guidelines.xhtml

Table 3: Summary of articles defining social inclusion and occupational justice in mental illness and HIV

Author(s) Year	Participants<br><br>Setting	Design	Aim	Definition(s) of social inclusion and or occupational justice	How operationalized	Associated key terms used
Harrison & Sellers<br><br>2008	Mental health service users and mental health team<br><br>United Kingdom	Opinion Paper	To explore the implications and challenges for occupational therapy roles in mental health services regarding socially inclusive practice and to discuss policy that is designed to broaden professional roles.	Occupational justice identifies inequalities in opportunities to participate in occupations.	As a human rights issue about participation in occupations<br><br>As a policy issue	social exclusion, participation, poverty, occupational deprivation
Hamer<br><br>2017	Mental health service users	Review	To discuss how social inclusion for mental health service users can be enhanced through occupational justice and the protection of their rights as citizens to have meaningful employment.	Social inclusion can be defined as the extent to which people are confident about and able to exercise their rights and participate, by choice, in the ordinary activities of citizens.<br><br>Occupational justice stated but not defined	As a human right issue	social exclusion, stigma, occupational justice, employment participation, poverty

For peer review only - http://bmjopen.bmj.com/site/about/guidelines.xhtml

For peer review only

Table 3: Summary of articles defining social inclusion and occupational justice in mental illness and HIV						
**Author(s) Year**	**Participants<br><br>Setting**	**Design**	**Aim**	**Definition(s) of social inclusion and or occupational justice**	**How operationalized**	**Associated key terms used**
Pettican & Bryant<br><br>2007	Mental health service users	Theoretical analysis	To explore the potential of occupational justice and its related concepts. To provide the occupational therapy profession with a theoretical justification for occupational therapists adopting an occupation-focused role in community mental health teams	Social inclusion is a drive aiming to overcome discrimination and stigma faced by people with mental health problems, in order to facilitate their having equal access to mainstream employment, education and leisure opportunities.<br><br>Occupational justice is recognising and providing for the occupational needs of individuals and communities as part of a fair and empowering society	Social inclusion conceptualized as a policy drive issue<br><br>As a justice and human rights issue<br>As an occupational participation issue	occupational deprivation, occupational imbalance, occupational alienation, social justice

For peer review only - http://bmjopen.bmj.com/site/about/guidelines.xhtml

Social inclusion: concepts and definitions

Social inclusion was defined with high variability, with only two studies using the same definition by defining social inclusion as a subjective sense of belonging and active citizenship that enhances social integration.[40 42] Conceptualisations and definitions used ambiguous words such as 'community,' 'participation,' and 'integration' to define social inclusion.. Some studies defined social inclusion in terms of paid work and participation in community events,[38 39] others focussed on social acceptance and absence of stigma,[40 42 45 56] while still others saw it as a political discourse.[41] Stain et al.[47] tried to capture these varieties, and defined social inclusion as

> "the participation of a person in society, evidenced by an individual having the opportunities, resources and abilities to build and maintain relationships, engage in education and employment, and participate in community events and organisations" (p.880).

Notable thematic areas emerged from the analysis of the definitions, namely community participation; human rights; and social relations that enhances a sense of acceptance and belonging.

Community participation

The most prominent shared features of the definitions of social inclusion in mental illness and HIV research focussed on it being a process and an experience centred on community participation. However, the terms 'community participation' and 'community' were used in many ways without clear descriptions. These varied interpretations of community participation included reference to people with HIV or mental illness were wide ranging and included individuals with opportunities to participate in key activities in their communities like paid employment[38 39 47 57]; being integrated into the community[37 41 45]; having a sense of belonging within the community[33 40 42 51]; and exercising active citizenship.[39 55 58]

Human rights

Social inclusion was also defined and conceptualised as a human rights issue, [39 46 49 56 58] even though community participation was the penultimate indicator for social inclusion. When discussing human rights, the authors highlighted an individual's right to access resources and opportunities for personal and community growth. Social inclusion was also conceptualised as

For peer review only - http://bmjopen.bmj.com/site/about/guidelines.xhtml

the right to engage in productive occupations, with full access to work and or educational activities within the community despite one's health concerns.[58]

Social relations

Social inclusion was further conceptualised and defined as a subjective experience[33 40 42 43 45 47] where those who are socially included should experience positive relationships. While a number of authors[33 40 42 43 45 47] talked about relationships as being a key component, this was not defined or discussed in depth. Instead it was emphasised that for social inclusion to be a reality, an individual should experience positive social relationships with their significant others, family, friends and acquaintances. [33 40 42 43 45 47] Social inclusion was also conceptualised as experiencing social support and having positive support networks.[45]

Diversity in definitions reflected in measurement tools

The diversity in definitions and features was also present in the measurement tool used. Quantitative studies included one of the following social inclusion measures: i) Social Inclusion Questionnaire;[43] ii) Social Relationships Scale and Social Inclusion Scale;[33] iii) Social Inclusion Interview Schedule;[45]; and iv) Social Inclusion Questionnaire User Experience.[38] Social inclusion was also portrayed in some definitions as a subjective personal concept where it is the individual with mental illness or person living with HIV, who subjectively experiences inclusion and should have a choice on what determines their experience of such inclusion.[34 40 42 44 51] It is their perception of the quality of their relationships, their acceptability to others, and how integrated they are, which was emphasised.

In summary, social inclusion was conceptualised as processes and experiences of empowered and equitable community participation for all, in which there is respect for human rights and healthy social relations and well-being are promoted.

Conceptualising and defining occupational justice

In the last two decades, only six research papers on mental illness used the concept of occupational justice and provided a definition. [35 36 46 50 52 54] (Table 3) Five different definitions were found (Table 3). As with social inclusion, occupational justice was defined with great variability, with it being referred to as both a process and as an experience. [35 36 46 50 52 54] Two major themes emerged pointing to social justice in which occupational justice was framed as an occupational rights issue and as a matter of community participation.

For peer review only - http://bmjopen.bmj.com/site/about/guidelines.xhtml

Occupational rights

A key concept of occupational justice for individuals with mental illness was experiencing or enjoying one's occupational rights. [35 36 46 50 52 54] Occupational rights were taken to mean an individual's rights to participate in a range of meaningful and contextual occupations enabling them to flourish, fulfil their potential and experience life satisfaction in ways consistent with their contexts. Townsend (2012) defined occupational justice as the enjoyment of 'occupational rights' by all people to engage in occupations and feel socially included in their desired occupations, thereby contributing positively to their own well-being and to that of their communities.[52] Occupational justice was also highlighted as an advocacy process where individuals could lobby for the occupational rights and needs of individuals and communities as part of an equitable, inclusive, and empowering society.[36]

Community participation

The process of promoting occupational justice was viewed as related to promoting social inclusion and community participation through advancing participation.[46] In another paper community participation was emphasised, with occupational justice defined as situations when people are seen as having the opportunity to choose to participate in the community.[35] The core emphasis in the definitions was the acknowledgement of occupational justice as a means of actioning community participation and social justice.[35 46]

However, in all the definitions there was an overuse of the term 'occupation' which forms part of the term we are working to define. While 'occupation' was framed as purposeful everyday activities people engage in, it carries diverse meanings outside occupational science and occupational therapy. This rendered many of the definitions and arguments cyclical in nature.

Commonalities between social inclusion and occupational justice

** Insert Figure 2: **Commonalities between occupational justice and social inclusion definitions – associated terms**

Although not clearly articulated, all the papers which discussed the two concepts together seemed to infer that occupational justice was an important ingredient of social inclusion.[35 36 46 52] Notwithstanding the considerable variability in definitions and conceptualisations of the two concepts, some commonalities were identified (Figure 2). Both social inclusion and

For peer review only - http://bmjopen.bmj.com/site/about/guidelines.xhtml

occupational justice seek to promote equitable access to opportunities for engagement and to seek for fairness and justice in an individual's community participation.[35 36 46 52]

Hamer et al. provided the closest account of the relationship between the concepts positing that social inclusion was enhanced through occupational justice.[46] Here they argued that recognition of people's right to inclusive participation in everyday occupations (occupational justice) enhanced the extent to which the person became confident about and was able to exercise their rights and participate by choice in the ordinary activities as citizens (social inclusion).[46] Both concepts were centred on key thematic areas of human rights, equality, inclusivity, and community participation.

Significantly, both social inclusion and occupational justice had a social justice focus, emphasizing the right to inclusive participation in a community and individuals exercising choice of participation as part of their citizenry beyond their health conditions. Occupational justice specifies that the participation in meaningful occupations is central, while social inclusion highlights community participation. Both concepts also highlight the need to address discriminatory practices, by doing away with stigma.[45 46] Hamer and colleagues highlight that social inclusion is the process of experiencing inclusive participation in the community as a citizen, while occupational justice promotes social inclusion through participation in meaningful and valued activities.[46]

Discussion

We explored and appraised the definitions, current utilisation, and relationships between social inclusion and occupational justice in the literature on mental illness or HIV. We identified and critically appraised 27 articles that presented a variety of definitions. Most studies were on mental illness and conducted in high-income countries. Despite the great variability, key thematic concepts used to define social inclusion and occupational justice included community participation, human rights, and relationships. The two concepts are theoretically related through a social justice focus, putting the emphasis on treating people with HIV and those with mental illness in a more respectful and equitable manner. The highlighted thematic concepts are central in directing research and practice toward the moral imperative of addressing exclusions and injustices experienced by people living with HIV, those with mental illnesses and other stigmatised groups. [21] There is also emphasis on the multidimensional nature of the concepts framed as both a process and a personal experience, also allowing a broader horizon

For peer review only - http://bmjopen.bmj.com/site/about/guidelines.xhtml

of their application, from policy to practice. The main source of these definitions were qualitative studies using service user's experiences and experts opinions.

While definitions of social inclusion vary, our scoping review findings confirm that the definitions are still in line with the concept of poverty reduction and a focus on reducing stigma and discrimination.[44 45 55] Social inclusion emerged from European societies, in response to a welfare crisis and desire to fight disadvantage.[59] We propose that this focus on welfare and fighting disadvantage should remain and should be taken up in the mental health and HIV fields, with poverty reduction, justice, and equality as pillars of social inclusion. This would be even more effective if poverty reduction was prioritised as a specific focus of social inclusion when informing mental health and HIV policies, particularly in sub-Saharan Africa, where these problems are more prevalent and driven by poverty. [3]

The most striking observation was the lack of clarity in the definition of social inclusion, which is still evident, despite its existence in the literature for almost five decades. [59] Efforts to be all inclusive, multidimensional, person-centred and contextual can explain the variations and, thus, ambiguity in the definitions.[59 60] However, this lack of definitional clarity could hamper its universal concept utilisation, measurement, and further exploration with a common goal.[61 62] This lack of a single, universal understanding has positive and negative implications for research and application of the concept in clinical practice. Without a universally agreed upon definition, comparisons between studies and practice remains difficult.[62] This was echoed in the variety of social inclusion measures applied in the quantitative studies.[33 38 43 45] Given the variation in measures, a common understanding of what constitutes social inclusion in mental health has not been developed, despite calls for this in recent literature.[61-63] Therefore construct validity of measures is difficult to test. For the meantime, measures with a broader scope and cross-cultural validation like the Social and Community Opportunities Profile (SCOPE),[64 65] can be instrumental in developing standardized measures.

In contrast, the variations in defining the concepts reflects diversity and the importance of contexts, rather than a singular adoption of a "universal" idea. Given the diversities in experiences of mental illness and living with HIV, where different regions have their own social, economic, political, cultural, and historical realities influencing mental illness and HIV, conceptualisations of social inclusion may benefit from remaining open to multiple definitions in order to reflect the realities of different regions. It is possible to have multiple, but mutual

For peer review only - http://bmjopen.bmj.com/site/about/guidelines.xhtml

interpretations that could be understood as complementary or even contesting, in line with contextual realities.

Embracing plural definitions may be especially important in low and middle-income countries, where such research needs further growth.[59] For example different activities purported to enhance social inclusion of people with mental health challenges, like paid employment, have different meanings and impact across regions and would influence how it is conceptualised as part of social inclusion. We found a preference towards paid employment as a key determinant of community participation in social inclusion amongst people with mental health challenges.[40 47 49 57 66] This can show the dominant knowledge systems of capitalism in societies[59] where most of the studies were conducted. Other communal ways of engagement outside of a neo-liberal market structure could be explored as they may facilitate new ways of understanding inclusion in the context of HIV and mental illness. In regions where HIV and mental illness are prevalent and complex health and social care problems, we advocate for exploration of social inclusion to generate contextual knowledge, that would inform socially inclusive policies, practice and further research.

Some of the social inclusion definitions had an individual focus, for example the individual had to be actively participating at the expense of the collective found in communal societies.[38 39 43] Given that the studies were mainly from high-income regions, there remains some missing voices in informing the definitions, given the largely communal orientation found in indigenous communities in low and middle-income settings. The challenge in some of the reviewed definitions was to try to focus on the individual and the impact of HIV and or mental illness, yet the social justice agenda may better be approached from a population level with a focus on broader social determinants of health which has been the case in many countries addressing developmental and intellectual disabilities. [67] Defining social inclusion of people with mental ill health and HIV, also needs to be done from the perspective of people who are in low-income countries, experiencing poverty, unemployment, social inequality and forms of violence since people with these conditions are usually stigmatised. Discrimination and consequent social problems usually affect people as part of a collective rather than only as individuals. Hence a collective perspective that considers how groups of people are affected can strengthen the concept of social inclusion and promote its possible utility in low-resource practice contexts.

Though the concept of occupational justice has been present in the literature for about three decades,[32] we found limited evidence of its conceptualisation and application in mental health

For peer review only - http://bmjopen.bmj.com/site/about/guidelines.xhtml

and no studies in HIV. [35 36 46 50 52 54] Despite the global-justice theoretical orientation of the concept, the studies found were also exclusively from high-income countries,.[35 36 46] We found key features that could guide occupational justice utilisation and further theorisation in HIV and mental health: community participation by having one's occupational rights upheld, occupational needs met, empowerment and equity in occupational participation. To some extent, the lack of diversity in regions informing the concept offers an opportunity to strengthen it by adding insights from regional contexts with potential for different realities, experiences and viewpoints, such as Africa where HIV and mental illness are prevalent and intertwined.[68] Also some authors conceptualised occupational justice as participation in occupation(s) in an equitable manner[46], they took participation as synonymous with justice, without spelling out the nature of the occupation and position of the person accessing the occupations. These have great potential in influencing how the accessed occupation impacts health, well-being and feeling of social inclusion amongst those with HIV and mental illnesses.

Despite the definitional lack of clarity, social inclusion and occupational justice are related concepts that can be used together to frame research and practice and inform policy in HIV and mental health. The commonality between the concepts is the need to promote equitable access to opportunities for community participation with fairness and equity for people with HIV and those suffering from mental illness. [35 36 46 52] The relationship between the concepts could be further developed using diverse communities to build evidence on how engagement in meaningful everyday activities underlie inclusive communities for people with mental illness and those living with HIV. This focus on occupational justice perspective presents an opportunity to routinely explore the nuances of everyday occupational participation and what that may mean for the process and experiences of social inclusion of those involved.

We therefore propose an expansion of the relationship between the conceptualisation of the two concepts, using most affected population groups, such as people with comorbid mental health disorders like substance use disorders and HIV in low-income contexts. This population group is known to have unmet broader health and social care needs hinged to the double stigma associated with substance abuse and HIV.[69] That expansion should reflect the contextual realities influencing community participation, respect of human rights, and having healthy relationships, actioned through engagement in meaningful occupations. These contextually refined concepts of social inclusion and occupational justice should then be used together to inform policy, research and practice, for a just and inclusive society for those with stigmatised conditions like HIV and mental illnesses. The occupational justice and socially inclusive

For peer review only

For peer review only - http://bmjopen.bmj.com/site/about/guidelines.xhtml

approach from policy through to practice, will ensure health and social well-being outcomes are addressed beyond the medical management of mental illness and or HIV. Health-related quality of life as a holistic construct for promoting continuum of care and health and well-being beyond viral suppression in HIV,[22 23] will also be made practical with a social justice lens.

Conclusion

To our knowledge, this scoping review is the first to appraise the concepts of occupational justice and social inclusion in populations afflicted by mental illness and HIV. Our findings have the potential to initiate critical conversations in the field and expand understanding and utilisation of occupational justice and social inclusion to critique and enhance global mental health. We have also presented commonalities which will give us a better theoretical foundation to inform further research, practice, and training, especially from underrepresented societies.

Author Contributions:

All authors have made substantive intellectual contributions to the conduct and write-up of this review. CN and RG conceptualised the review approach and provided general guidance to the research team. Then CN and EM were involved in systematic and independent screening and data extraction. CN provided primary input at all stages, developed all draft documents and had overall responsibility for the review. LL, RG and RH gave substantial review and critique through the review process and manuscript. All the authors reviewed and commented on the drafts of the manuscript and they all read and approved the final manuscript.

Acknowledgements:

We would like to thank Mary Shelton – UCT librarian for assistance in developing our search strategy. This article also benefited from writing soft skills and reviews by Dr Helen Jack through African Mental Health Research Initiative (AMARI).

Funding: This work was supported through the DELTAS Africa Initiative [DEL-15-01]. The DELTAS Africa Initiative is an independent funding scheme of the African Academy of Sciences (AAS)'s Alliance for Accelerating Excellence in Science in Africa (AESA) and supported by the New Partnership for Africa's Development Planning and Coordinating Agency (NEPAD Agency) with funding from the Wellcome Trust [DEL-15-01] and the UK government. The views expressed in this publication are those of the author(s) and not necessarily those of AAS, NEPAD Agency, Wellcome Trust or the UK government.

For peer review only - http://bmjopen.bmj.com/site/about/guidelines.xhtml

For peer review only

Competing interests: None.

Data availability statement: No additional data available.

References

1. UNAIDS. UNAIDS data 2019. Geneva, 2019.
2. Pandey A, Galvani AP. The global burden of HIV and prospects for control. *The Lancet HIV* doi: 10.1016/S2352-3018(19)30230-9
3. UNAIDS. Global AIDS update 2019 — Communities at the centre — Defending rights, breaking barriers, reaching people with HIV services. Geneva, 2019.
4. Bauer-Staeb C, Jorgensen L, Lewis G, et al. Prevalence and risk factors for HIV, hepatitis B, and hepatitis C in people with severe mental illness: a total population study of Sweden. *Lancet Psychiatry* 2017;4(9):685-93. doi: 10.1016/s2215-0366(17)30253-5 [published Online First: 2017/07/09]
5. Hughes E, Bassi S, Gilbody S, et al. Prevalence of HIV, hepatitis B, and hepatitis C in people with severe mental illness: a systematic review and meta-analysis. *Lancet Psychiatry* 2016;3(1):40-48. doi: 10.1016/s2215-0366(15)00357-0 [published Online First: 2015/12/02]
6. Patel P, Rose CE, Collins PY, et al. Noncommunicable diseases among HIV-infected persons in low-income and middle-income countries: a systematic review and meta-analysis. *AIDS* 2018;32(Suppl 1):S5.
7. Bhatia R, Hartman C, Kallen MA, et al. Persons newly diagnosed with HIV infection are at high risk for depression and poor linkage to care: results from the Steps Study. *AIDS and Behavior* 2011;15(6):1161-70.
8. Mayston R, Kinyanda E, Chishinga N, et al. Mental disorder and the outcome of HIV/AIDS in low-income and middle-income countries: a systematic review. *AIDS* 2012;26:S117-S35.
9. Nyongesa MK, Mwangala PN, Mwangi P, et al. Neurocognitive and mental health outcomes and association with quality of life among adults living with HIV: a cross-sectional focus on a low-literacy population from coastal Kenya. *BMJ Open* 2018;8(9):e023914.
10. Lowther K, Selman L, Harding R, et al. Experience of persistent psychological symptoms and perceived stigma among people with HIV on antiretroviral therapy (ART): a systematic review. *Int J Nurs Stud* 2014;51(8):1171-89. doi: 10.1016/j.ijnurstu.2014.01.015 [published Online First: 2014/03/08]
11. Sin NL, DiMatteo MR. Depression treatment enhances adherence to antiretroviral therapy: a meta-analysis. *Annals of Behavioral Medicine* 2014;47(3):259-69.
12. UNAIDS. THEMATIC SEGMENT: MENTAL HEALTH AND HIV/AIDS PROMOTING HUMAN RIGHTS, AN INTEGRATED AND PERSON-CENTRED APPROACH TO IMPROVING ART ADHERENCE, WELL-BEING AND QUALITY OF LIFE. Geneva, 2018.
13. Jackson-Best F, Edwards N. Stigma and intersectionality: a systematic review of systematic reviews across HIV/AIDS, mental illness, and physical disability. *BMC Public Health* 2018;18(1):919. doi: 10.1186/s12889-018-5861-3
14. Aidala AA, Wilson MG, Shubert V, et al. Housing Status, Medical Care, and Health Outcomes Among People Living With HIV/AIDS: A Systematic Review. *American Journal of Public Health* 2016;106(1):e1-e23. doi: 10.2105/ajph.2015.302905
15. Breuer E, Myer L, Struthers H, et al. HIV/AIDS and mental health research in sub-Saharan Africa: a systematic review. *Afr J AIDS Res* 2011;10(2):101-22. doi: 10.2989/16085906.2011.593373 [published Online First: 2011/06/01]
16. Levi-Minzi MA, Surratt HL. HIV stigma among substance abusing people living with HIV/AIDS: implications for HIV treatment. *AIDS patient care and STDs* 2014;28(8):442-51.
17. Durocher E, Rappolt S, Gibson BE. Occupational justice: Future directions. *J Occup Sci* 2013;21(4) doi: 10.1080/14427591.2013.775693

For peer review only - http://bmjopen.bmj.com/site/about/guidelines.xhtml

18. Nilsson I, Townsend E. Occupational Justice—Bridging theory and practice. *Scand J Occup Ther* 2010;17(1):57-63. doi: 10.3109/11038120903287182
19. Cobigo V, Stuart H. Social inclusion and mental health. *Curr Opin Psychiatry* 2010;23(5):453-57. doi: 10.1097/YCO.0b013e32833bb305
20. Pereira RB, Whiteford GE. Understanding social inclusion as an international discourse: Implications for enabling participation. *Br J Occup Ther* 2013;76(2):112-15. doi: 10.4276/030802213X13603244419392
21. Nilsson I, Townsend E. Occupational justice—Bridging theory and practice. *Scandinavian journal of occupational therapy* 2010;17(1):57-63.
22. Lazarus JV, Safreed-Harmon K, Barton SE, et al. Beyond viral suppression of HIV – the new quality of life frontier. *BMC Medicine* 2016;14(1):94. doi: 10.1186/s12916-016-0640-4
23. Kall M, Marcellin F, Harding R, et al. Patient-reported outcomes to enhance person-centred HIV care. *The Lancet HIV* doi: 10.1016/S2352-3018(19)30345-5
24. Baumgartner JN, Burns JK. Measuring social inclusion-a key outcome in global mental health. *Int J Epidemiol* 2014;43(2):354-64. doi: 10.1093/ije/dyt224
25. Patel V, Saxena S, Frankish H, et al. Sustainable development and global mental health—a Lancet Commission. *Lancet* 2016;387(10024):1143-45. doi: 10.1016/S0140-6736(16)00208-7
26. McKinstry C, Brown T, Gustafsson L. Scoping reviews in occupational therapy: The what, why, and how to. *Australian occupational therapy journal* 2014;61(2):58-66.
27. Nhunzvi C, Langhaug L, Mavindidze E, et al. Occupational justice and social inclusion in mental illness and HIV: a scoping review protocol. *BMJ open* 2019;9(3):e024049.
28. Grant MJ, Booth A. A typology of reviews: An analysis of 14 review types and associated methodologies. *Health Info Libr J* 2009;26:91-108. doi: 10.1111/j.1471-1842.2009.00848.x
29. Arksey H, Malley LO. Scoping Studies: Towards a Methodological Framework. *Int J Soc Res Methodol* 2005;8(1):19-32. doi: 10.1080/1364557032000119616
30. Halas G, Schultz AS, Rothney J, et al. A scoping review protocol to map the research foci trends in tobacco control over the last decade. *BMJ open* 2015;5(1):e006643.
31. Tricco AC, Lillie E, Zarin W, et al. PRISMA Extension for Scoping Reviews (PRISMA-ScR): Checklist and Explanation. *Ann Intern Med* 2018;169(7):467-73. doi: 10.7326/m18-0850 [published Online First: 2018/09/05]
32. Serrata Malfitano AP, Gomes da Mota de Souza R, Esquerdo Lopes R. Occupational justice and its related concepts: An historical and thematic scoping review. *OTJR: Occupation, participation and health* 2016;36(4):167-78.
33. Berry C, Greenwood K. Direct and indirect associations between dysfunctional attitudes, self-stigma, hopefulness and social inclusion in young people experiencing psychosis. *Schizophrenia Research* 2017 doi: 10.1016/j.schres.2017.06.037
34. Clewes J, Shivamurthy S, Wrigley S. Brightening His Days: A Reflective Single Case-Study of Social Integration and Recovery. *Occupational Therapy in Mental Health* 2013;29(2):159-80.
35. Farrell C, Bryant W. Voluntary work for adults with mental health problems: An exploration of the perspectives of recruiters. *The British Journal of Occupational Therapy* 2009;72(5):188-96. doi: 10.1177/030802260907200502
36. Fieldhouse J. Community participation and recovery for mental health service users: an action research inquiry. *British Journal of Occupational Therapy* 2012;75(9):419-28.
37. Fieldhouse J. Mental health, social inclusion, and community development: lessons from Bristol. *Community Development Journal* 2012;47(4):571-87. doi: 10.1093/cdj/bss028
38. Killaspy H, White S, Lalvani N, et al. The impact of psychosis on social inclusion and associated factors. *International Journal of Social Psychiatry* 2014;60(2):148-54. doi: 10.1177/0020764012471918
39. Mazzi F, Baccari F, Mungai F, et al. Effectiveness of a social inclusion program in people with non-affective psychosis. *BMC Psychiatry* 2018;18(1) doi: 10.1186/s12888-018-1728-5

For peer review only - http://bmjopen.bmj.com/site/about/guidelines.xhtml

40. Nieminen I, Ramon S, Dawson I, et al. Experiences of social inclusion and employment of mental health service users in a european union project. *International Journal of Mental Health* 2012;41(4):3-23. doi: 10.2753/IMH0020-7411410401
41. Raitakari S, Haahtela R, Juhila K. Tackling community integration in mental health home visit integration in Finland. *Health & Social Care in the Community* 2016;24(5):E53-E62. doi: 10.1111/hsc.12246
42. Ramon S, Griffiths CA, Nieminen I, et al. Towards social inclusion through lifelong learning in mental health: Analysis of change in the lives of the EMILIA project service users. *International Journal of Social Psychiatry* 2011;57(3):211-23. doi: 10.1177/0020764009354943
43. Saavedra J, Pérez E, Crawford P, et al. Recovery and creative practices in people with severe mental illness: evaluating well-being and social inclusion. *Disability and Rehabilitation* 2018;40(8):905-11. doi: 10.1080/09638288.2017.1278797
44. Smyth G, Harries P, Dorer G. Exploring mental health service users' experiences of social inclusion in their community occupations. *British Journal of Occupational Therapy* 2011;74(7):323-31. doi: 10.4276/030802211X13099513661072
45. Turner N, Ferguson L, Hill M, et al. An exploratory study of the extent of social inclusion among people with psychosis and psychotic-related conditions. *International Journal of Social Psychiatry* 2017;63(3):195-202. doi: 10.1177/0020764017691551
46. Hamer HP, Kidd J, Clarke S, et al. Citizens un-interrupted: Practices of inclusion by mental health service users. *Journal of Occupational Science* 2017;24(1):76-87. doi: 10.1080/14427591.2016.1253497
47. Stain HJ, Galletly CA, Clark S, et al. Understanding the social costs of psychosis: The experience of adults affected by psychosis identified within the second Australian national survey of psychosis. *Australian and New Zealand Journal of Psychiatry* 2012;46(9):879-89. doi: 10.1177/0004867412449060
48. Moraes Salles M, Barros Sn. THE SOCIAL EXCLUSION/INCLUSION OF USERS OF A PSYCHOSOCIAL CARE CENTER IN EVERYDAY LIFE. *Texto & Contexto Enfermagem* 2013;22(3):704-12.
49. Salles MM, Barros S. Social representation of users of a Psychosocial Care Center and those in their social network on mental illness and social inclusion. *Saude E Sociedade* 2013;22(4):1059-71. doi: 10.1590/s0104-12902013000400009
50. Harrison D, Sellers A. Occupation for mental health and social inclusion. *British Journal of Occupational Therapy* 2008;71(5):216-19.
51. Le Boutillier C, Croucher A. Social inclusion and mental health. *British Journal of Occupational Therapy* 2010;73(3):136-39.
52. Townsend EA. Boundaries and bridges to adult mental health: Critical occupational and capabilities perspectives of justice. *Journal of Occupational Science* 2012;19(1):8-24.
53. Mandiberg JM. Commentary: the failure of social inclusion: an alternative approach through community development. *Psychiatric Services* 2012;63(5):458-60.
54. Pettican A, Bryant W. Sustaining a focus on occupation in community mental health practice. *British Journal of Occupational Therapy* 2007;70(4):140-46.
55. Cáceres CF, Aggleton P, Galea JT. Sexual diversity, social inclusion and HIV/AIDS

AIDS 2008;22(Suppl2):S45-S55.
56. Cobigo V, Stuart H. Social inclusion and mental health. *Current Opinion in Psychiatry* 2010;23(5):453-57. doi: 10.1097/YCO.0b013e32833bb305
57. Evans J, Repper J. Employment, social inclusion and mental health. *Journal of Psychiatric and Mental Health Nursing* 2000;7(1):15-24. doi: 10.1046/j.1365-2850.2000.00260.x
58. Hamer H. Returning citizens: Social inclusion and occupational justice for mental health service users. *Cadernos Brasileiros de Saúde Mental/Brazilian Journal of Mental Health* 2017;9(21):89-113.
59. Rawal N. Social inclusion and exclusion: A review. *Dhaulagiri Journal of Sociology and Anthropology* 2008;2:161-80.

For peer review only

60. Silver H. The contexts of social inclusion. 2015.
61. Baumgartner JN, Burns JK. Measuring social inclusion—a key outcome in global mental health. *International journal of epidemiology* 2013;43(2):354-64.
62. O'Donnell P, O'Donovan D, Elmusharaf K. Measuring social exclusion in healthcare settings: a scoping review. *International journal for equity in health* 2018;17(1):15.
63. Huxley P, Thornicroft G. Social inclusion, social quality and mental illness. *The British Journal of Psychiatry* 2003;182(4):289-90.
64. Huxley PJ, Chan K, Chiu M, et al. The social and community opportunities profile social inclusion measure: Structural equivalence and differential item functioning in community mental health residents in Hong Kong and the United Kingdom. *International Journal of Social Psychiatry* 2016;62(2):133-40.
65. Chan K, Evans S, Ng YL, et al. A Concept Mapping Study on Social Inclusion in Hong Kong. *Social Indicators Research* 2014;119(1):121-37. doi: 10.1007/s11205-013-0498-1
66. Lloyd C, Tse S, Deane FP. Community participation and social inclusion: How practitioners can make a difference. *AeJAMH (Australian e-Journal for the Advancement of Mental Health)* 2006;5(3):1-10. doi: 10.5172/jamh.5.3.185
67. Martin L, Cobigo V. Definitions matter in understanding social inclusion. *Journal of Policy and Practice in Intellectual Disabilities* 2011;8(4):276-82.
68. Brandt R. The mental health of people living with HIV/AIDS in Africa: a systematic review. *African Journal of AIDS Research* 2009;8(2):123-33.
69. Duffy M, Sharer M, Cornman H, et al. Integrating Mental Health and HIV Services in Zimbabwean Communities: A Nurse and Community-led Approach to Reach the Most Vulnerable. *Journal of the Association of Nurses in AIDS Care* 2015:1-13. doi: 10.1016/j.jana.2015.09.003

For peer review only

For peer review only - http://bmjopen.bmj.com/site/about/guidelines.xhtml

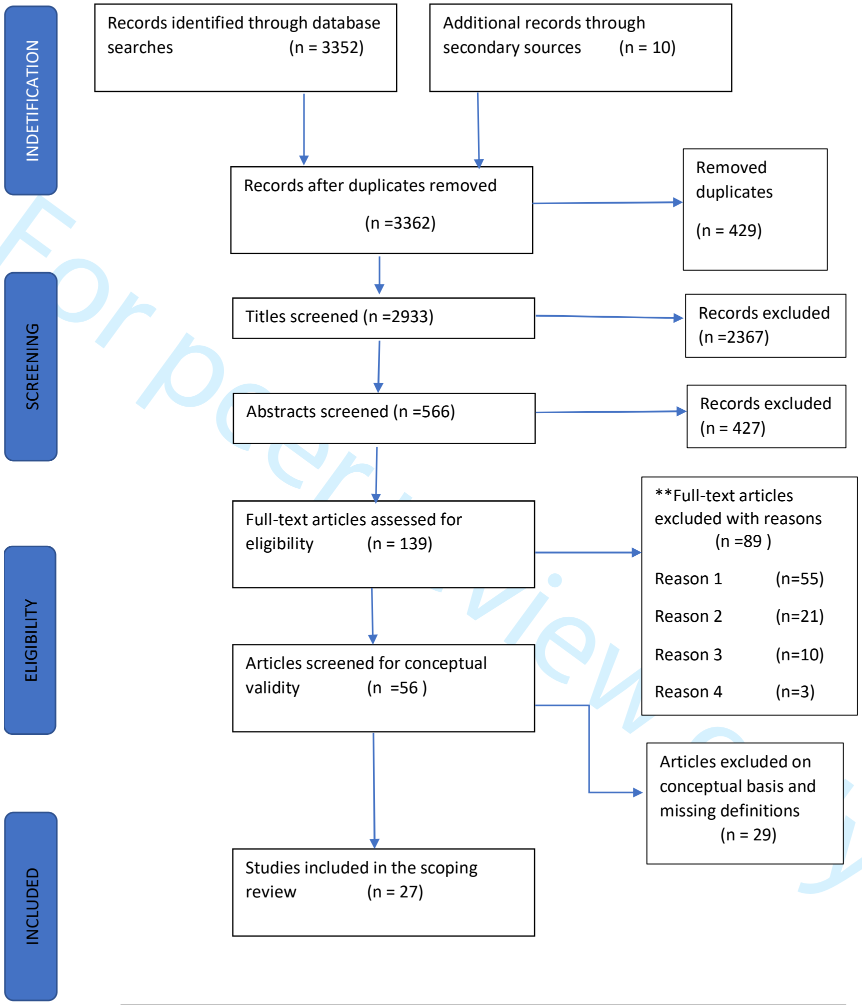

Reasons for full-text articles exclusion
(n =89)

Reason 1 (n=55): No explicit mention of either of the concepts or their related terms

Reason 2 (n=21): Not applied to target population groups, that is HIV and or mental illness

Reason 3 (n=10): No English version of full article

Reason 4 (n=3): Book reviews/chapters

Figure 1: Scoping review flow diagram

For peer review only

Figure 2: Commonalities between occupational justice and social inclusion definitions – associated terms

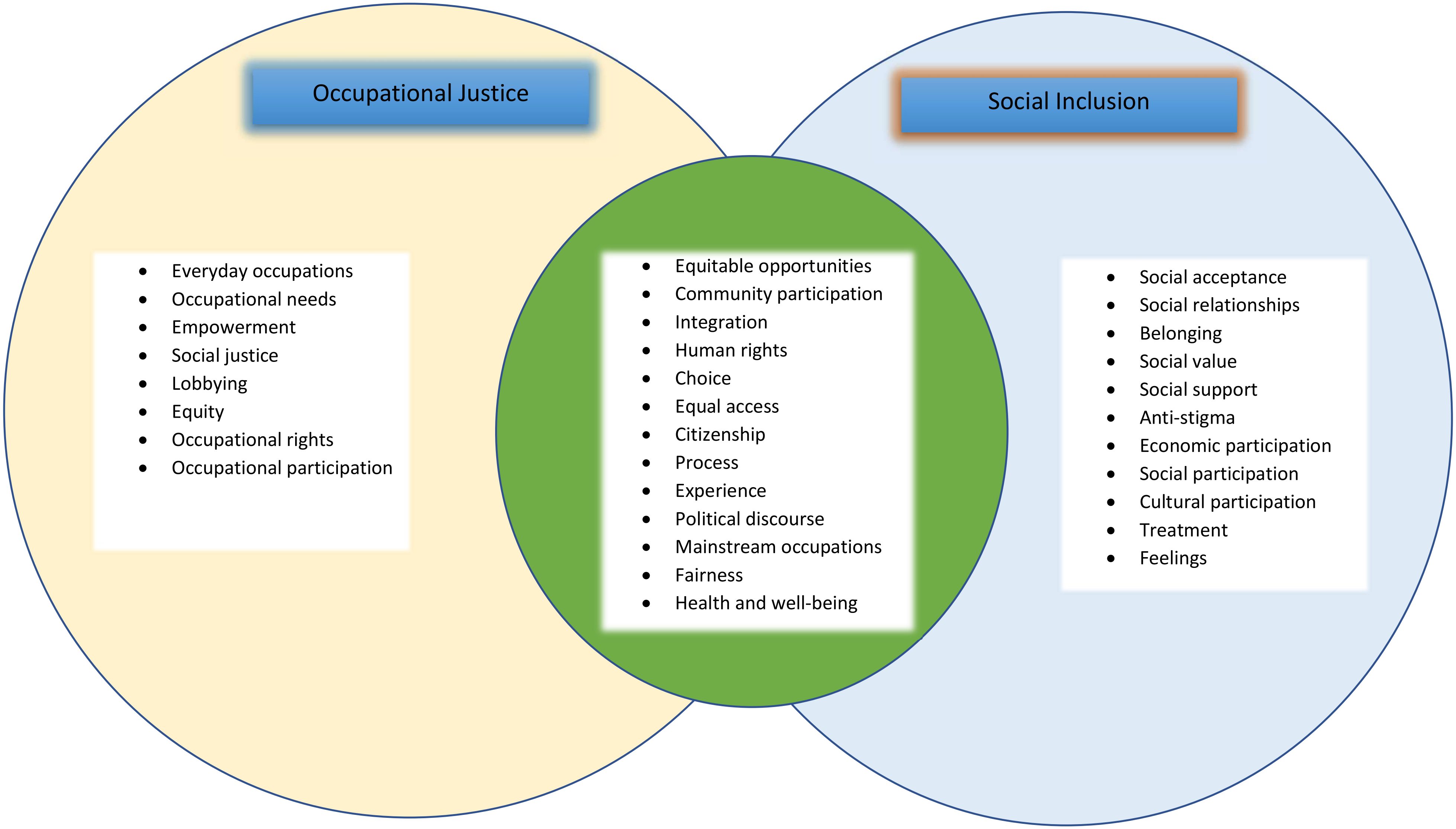

Appendix 1. PubMed Search Strategy

PubMed

Set 1

1. Social Justice [MeSH]

2. Social Isolation [MeSH]

3. Social Marginalization [MeSH]

4. Social Participation [MeSH]

5. Rehabilitation, Vocational [MeSH]

6. Injustice OR justice OR social inclusion OR social exclusion OR social isolation OR social separation OR social barriers OR social distance OR social acceptance OR social rejection OR social participation OR deprivation OR marginalization OR alienation

7. 1 OR 2 OR 3 OR 4 OR 5 OR 6 *(represents Social inclusion set)*

Set 2

1. Social stigma [MeSH]

2. Prejudice [MeSH]

3. Stigma OR prejudice OR stigmatise OR stigmatisation OR stigmatize OR stigmatization OR discrimination

4. 1 OR 2 OR 3 *(represents Stigma set)*

Set 3

1. Occupational justice OR Occupational injustice OR Occupational deprivation OR Occupational alienation OR Occupational marginalisation OR Occupational imbalance OR Occupational OR occupation OR occupations OR activities OR work OR employment OR unemployment OR engagement (*this set is used to narrow search to occupation as defined by OT not PubMed's definition)*

Set 4

1. Mental Disorders [MeSH] *(this heading includes substance-related disorders)*

2. Mentally Ill Persons [MeSH]

For peer review only

3. Mental disorders OR mental illness OR mentally ill OR Psychiatric disorder OR psychiatric illness OR psychological disorder OR Developmental Disability OR Intellectual Development Disorder OR Intellectual disability OR Mental retardation OR Mental deficiency
4. 1 OR 2 OR 3 *(represents Mental Disorders set)*

Set 5

1. HIV [MeSH]
2. HIV Infections [MeSH]
3. Acquired Immunodeficiency Syndrome (MeSH)
4. HIV OR human immune deficiency virus OR AIDS OR acquired immunodeficiency syndrome OR acquired immune deficiency syndrome OR HIV/AIDS
5. 1 OR 2 OR 3 OR 4

Now combine Sets, 1 AND 2 AND 3 AND 4 or Sets 1 AND 2 AND 3 AND 5
Limit to last 20 years

English

Date: 31/01/19

For peer review only

Preferred Reporting Items for Systematic reviews and Meta-Analyses extension for Scoping Reviews (PRISMA-ScR) Checklist

SECTION	ITEM	PRISMA-ScR CHECKLIST ITEM	REPORTED ON PAGE #
**TITLE**			
Title	1	Identify the report as a scoping review.	Page 1
**ABSTRACT**			
Structured summary	2	Provide a structured summary that includes (as applicable): background, objectives, eligibility criteria, sources of evidence, charting methods, results, and conclusions that relate to the review questions and objectives.	Page 2
**INTRODUCTION**			
Rationale	3	Describe the rationale for the review in the context of what is already known. Explain why the review questions/objectives lend themselves to a scoping review approach.	Page 6
Objectives	4	Provide an explicit statement of the questions and objectives being addressed with reference to their key elements (e.g., population or participants, concepts, and context) or other relevant key elements used to conceptualize the review questions and/or objectives.	Page 6
**METHODS**			
Protocol and registration	5	Indicate whether a review protocol exists; state if and where it can be accessed (e.g., a Web address); and if available, provide registration information, including the registration number.	Yes ref 27
Eligibility criteria	6	Specify characteristics of the sources of evidence used as eligibility criteria (e.g., years considered, language, and publication status), and provide a rationale.	Page 7
Information sources*	7	Describe all information sources in the search (e.g., databases with dates of coverage and contact with authors to identify additional sources), as well as the date the most recent search was executed.	Page 7
Search	8	Present the full electronic search strategy for at least 1 database, including any limits used, such that it could be repeated.	Page 7. Appendix 1
Selection of sources of evidence†	9	State the process for selecting sources of evidence (i.e., screening and eligibility) included in the scoping review.	Page 7
Data charting process‡	10	Describe the methods of charting data from the included sources of evidence (e.g., calibrated forms or forms that have been tested by the team before their use, and whether data charting was done independently or in duplicate) and any processes for obtaining and confirming data from investigators.	Page 8
Data items	11	List and define all variables for which data were sought and any assumptions and simplifications made.	Page 11. Table 3
Critical appraisal of individual sources of evidence§	12	If done, provide a rationale for conducting a critical appraisal of included sources of evidence; describe the methods used and how this information was used in any data synthesis (if appropriate).	Click here to enter text.

St. Michael's
Inspired Care. Inspiring Science.

For peer review only - http://bmjopen.bmj.com/site/about/guidelines.xhtml

SECTION	ITEM	PRISMA-ScR CHECKLIST ITEM	REPORTED ON PAGE #
Synthesis of results	13	Describe the methods of handling and summarizing the data that were charted.	Page 8
**RESULTS**			
Selection of sources of evidence	14	Give numbers of sources of evidence screened, assessed for eligibility, and included in the review, with reasons for exclusions at each stage, ideally using a flow diagram.	Page 9. Figure 1
Characteristics of sources of evidence	15	For each source of evidence, present characteristics for which data were charted and provide the citations.	Page 11-22. Table 3
Critical appraisal within sources of evidence	16	If done, present data on critical appraisal of included sources of evidence (see item 12).	Click here to enter text.
Results of individual sources of evidence	17	For each included source of evidence, present the relevant data that were charted that relate to the review questions and objectives.	Table 3
Synthesis of results	18	Summarize and/or present the charting results as they relate to the review questions and objectives.	Page 11-22
**DISCUSSION**			
Summary of evidence	19	Summarize the main results (including an overview of concepts, themes, and types of evidence available), link to the review questions and objectives, and consider the relevance to key groups.	Page 26-29
Limitations	20	Discuss the limitations of the scoping review process.	Page 2
Conclusions	21	Provide a general interpretation of the results with respect to the review questions and objectives, as well as potential implications and/or next steps.	Page 30
**FUNDING**			
Funding	22	Describe sources of funding for the included sources of evidence, as well as sources of funding for the scoping review. Describe the role of the funders of the scoping review.	Page 31

JBI = Joanna Briggs Institute; PRISMA-ScR = Preferred Reporting Items for Systematic reviews and Meta-Analyses extension for Scoping Reviews.

* Where *sources of evidence* (see second footnote) are compiled from, such as bibliographic databases, social media platforms, and Web sites.

† A more inclusive/heterogeneous term used to account for the different types of evidence or data sources (e.g., quantitative and/or qualitative research, expert opinion, and policy documents) that may be eligible in a scoping review as opposed to only studies. This is not to be confused with *information sources* (see first footnote).

‡ The frameworks by Arksey and O'Malley (6) and Levac and colleagues (7) and the JBI guidance (4, 5) refer to the process of data extraction in a scoping review as data charting.

§ The process of systematically examining research evidence to assess its validity, results, and relevance before using it to inform a decision. This term is used for items 12 and 19 instead of "risk of bias" (which is more applicable to systematic reviews of interventions) to include and acknowledge the various sources of evidence that may be used in a scoping review (e.g., quantitative and/or qualitative research, expert opinion, and policy document).

From: Tricco AC, Lillie E, Zarin W, O'Brien KK, Colquhoun H, Levac D, et al. PRISMA Extension for Scoping Reviews (PRISMAScR): Checklist and Explanation. Ann Intern Med. 2018;169:467–473. doi: 10.7326/M18-0850.

St. Michael's
Inspired Care.
Inspiring Science.

For peer review only - http://bmjopen.bmj.com/site/about/guidelines.xhtml

BMJ Open

Occupational justice and social inclusion amongst people living with HIV and people with mental illness: A scoping review

Journal:	*BMJ Open*
Manuscript ID	bmjopen-2020-036916.R2
Article Type:	Original research
Date Submitted by the Author:	31-May-2020
Complete List of Authors:	NHUNZVI, CLEMENT; University of Zimbabwe, College of Health Sciences, Rehabilitation Department<br>Langhaug, Lisa; African Mental Health Research Initiative (AMARI) , Department of Psychiatry, College of Health Sciences. University of Zimbabwe.<br>Mavindidze, Edwin; Ingutsheni Central Hospital, Occupational Therapy<br>Harding, Richard; King's College London, Florence Nightingale Faculty of Nursing Midwifery and Palliative Care, Cicely Saunders Institute.<br>Galvaan, Roshan ; University of Cape Town, Health and Rehabilitation Sciences
**Primary Subject Heading**:	Mental health
Secondary Subject Heading:	HIV/AIDS, Mental health, Public health, Rehabilitation medicine
Keywords:	MENTAL HEALTH, HIV & AIDS < INFECTIOUS DISEASES, REHABILITATION MEDICINE, PUBLIC HEALTH

SCHOLARONE™
Manuscripts

I, the Submitting Author has the right to grant and does grant on behalf of all authors of the Work (as defined in the below author licence), an exclusive licence and/or a non-exclusive licence for contributions from authors who are: i) UK Crown employees; ii) where BMJ has agreed a CC-BY licence shall apply, and/or iii) in accordance with the terms applicable for US Federal Government officers or employees acting as part of their official duties; on a worldwide, perpetual, irrevocable, royalty-free basis to BMJ Publishing Group Ltd ("BMJ") its licensees and where the relevant Journal is co-owned by BMJ to the co-owners of the Journal, to publish the Work in this journal and any other BMJ products and to exploit all rights, as set out in our licence.

The Submitting Author accepts and understands that any supply made under these terms is made by BMJ to the Submitting Author unless you are acting as an employee on behalf of your employer or a postgraduate student of an affiliated institution which is paying any applicable article publishing charge ("APC") for Open Access articles. Where the Submitting Author wishes to make the Work available on an Open Access basis (and intends to pay the relevant APC), the terms of reuse of such Open Access shall be governed by a Creative Commons licence – details of these licences and which Creative Commons licence will apply to this Work are set out in our licence referred to above.

Other than as permitted in any relevant BMJ Author's Self Archiving Policies, I confirm this Work has not been accepted for publication elsewhere, is not being considered for publication elsewhere and does not duplicate material already published. I confirm all authors consent to publication of this Work and authorise the granting of this licence.

For peer review only

Title: Occupational justice and social inclusion amongst people living with HIV and people with mental illness: A scoping review.

*Clement Nhunzvi[1,2,3]; Lisa Langhaug[3]; Edwin Mavindidze[4]; Richard Harding[5] & Roshan Galvaan[2]

* **Corresponding author: Clement Nhunzvi**

Department of Rehabilitation
University of Zimbabwe, College of Health Sciences
P.O. Box A178
Avondale, Harare
Zimbabwe
clemynhu@gmail.com; nhncle001@myuct.ac.za
TEL: +263 4 797800 Cell: +263 775 814 587

Affiliations

[1] Department of Rehabilitation, College of Health Sciences. University of Zimbabwe. Zimbabwe.

[2] Division of Occupational Therapy, Department of Health and Rehabilitation Sciences, Faculty of Health Sciences, University of Cape Town. South Africa.

[3] African Mental Health Research Initiative (AMARI), Department of Psychiatry, College of Health Sciences. University of Zimbabwe. Zimbabwe.

[4] Occupational Therapy Department, Ingutsheni Central Hospital, Bulawayo. Zimbabwe.

[5] Florence Nightingale Faculty of Nursing Midwifery and Palliative Care, Kings College London, Cicely Saunders Institute. UK.

Keywords: social inclusion, occupational justice, scoping review, HIV, mental illness

Word count: 4587

For peer review only

Abstract

Objective To explore ways in which occupational justice and social inclusion are conceptualised, defined, and operationalised in highly stigmatised and chronic conditions of mental illness and HIV.

Design This scoping review protocol followed Arksey and O`Malley`s (2005) Scoping Review Framework.

Data sources and eligibility. The following databases were searched for the period January 1997 – January 2019: Medline via PubMed, Scopus, Academic Search Premier, Cumulative Index to Nursing and Allied Health Literature (CINAHL), Africa-Wide Information, Humanities International Complete, Web of Science, PsychInfo, SocINDEX, and grey literature.

Eligible articles were primary studies, reviews or theoretical papers which conceptualised, defined and/or operationalised social inclusion or occupational justice in mental illness or HIV.

Study appraisal and synthesis. We undertook a three-part article screening process. Screening and data extraction were undertaken independently by two researchers. Arksey's framework and thematic analysis informed the collation and synthesis of included papers.

Results. From 3352 records, we reviewed 139 full articles and retained 27 for this scoping review. Definitions of social inclusion and occupational justice in the domains of mental illness and HIV were heterogeneous and lacked definitional clarity. The two concepts were conceptualised as either processes or personal experiences, with key features of community participation, respect for human rights, and establishment and maintenance of healthy relationships. Conceptual commonalities between social inclusion and occupational justice were premised on social justice.

Conclusions To address lack of clarity, we propose further and concurrent exploration of these concepts, specifically with reference to persons with comorbid mental health disorders such as substance use disorders and HIV living in low-income countries. This should reflect contextual realities influencing community participation, respect for human rights, and meaningful occupational participation. From this broadened understanding, quantitative measures should be applied to improve the standardization of measurements for occupational justice and social inclusion in policy, research and practice.

For peer review only - http://bmjopen.bmj.com/site/about/guidelines.xhtml

Title: Occupational justice and social inclusion among people living with HIV and people with mental illness: A scoping review.

Article Summary

Strengths and limitations of this study

- The methodology as provided by the scoping review design, facilitated comprehensive mapping of the literature, and presented a foundation for further exploration utilisation of the concepts to inform policy, research and practice.
- We used a rigorous strategy to explore research foci, definitions and utilisations of the concepts of social inclusion and occupational justice in mental illness and HIV.
- Data synthesis was limited to work published in English originally or with available English translated copies.
- We focused on mental illness, which is made up of several different conditions and could have introduced generalisation bias. However, most of the included studies were also not condition specific, fitting our primary aim for conceptual review.
- Focus was limited to conceptual and theoretical aspects of the concepts more than interventions and outcomes of interventions.

For peer review only

Introduction -

The global burden of disease from HIV remains substantially high with approximately 37.9 million people living with HIV[1] However, in the last two decades, the world has seen a combination of a significantly decreased mortality and a small decrease in incidence leading to an increase in the number of people living with HIV from 8·74 million (1990) to 36·9 million (2017).[2]This increase in the number of people living with HIV and decreased mortality rates are largely a result of the scaling up of HIV treatments.[1 3] However, what remains as a concern are persisting gaps in the treatment continuum towards the UNAIDS 90-90-90 target. Among those living with HIV who knew their status globally, 17% were still not on life-saving antiretroviral therapy UNAIDS Global AIDS update 2019.[1] Moreover, only 53% of those on treatment were virally suppressed. [1 3] One reason for these discrepancies, mainly seen in key populations, is the rising and ever complex relationship between mental illness and HIV.[4 5] Secondary to both biological and psychosocial factors, people living with HIV are at an increased risk of experiencing poor mental health [6 7] which negatively impacts on their health-seeking behaviours, adherence to antiretroviral treatments [8] and quality of life.[9] The prevalence of common mental disorders is also significantly higher among people living with HIV, irrespective of their being on ART, and is further impounded by stigma.[10] There is also a known bidirectional relationship between HIV and mental health, worsened by associated health and social inequalities. This often leaves people with severe mental illnesses at an increased risk for HIV infection.[11 12]

Individuals with chronic and usually stigmatised conditions such as mental illnesses, physical disabilities and HIV, face barriers to full participation in their communities.[13] Poverty, lack of access to education, lack of suitable housing, and unemployment are some of the social and economic barriers to accessing adequate and sustained healthcare faced by this group.[14] These barriers may similarly be experienced by people who face discrimination based on their class, race or gender identity or sexuality and thus, when persons with mental illness also share these characteristics they may be severely stigmatised or discriminated against.[15 16]

To sustain the aforementioned progress in the management of people with HIV and its comorbidities such as mental illness, there is a renewed call to take a community-led, equality and social justice approach[3 12] with concepts like occupational justice and social inclusion holding promise to inform this agenda. Occupational justice is an advanced form of social justice, concerned with equity and fairness for individuals, groups and communities access to

For peer review only - http://bmjopen.bmj.com/site/about/guidelines.xhtml

resources and opportunities that supports their engagement in diverse, healthy, and meaningful occupations.[17 18] On the other hand, social inclusion entails multi-dimensional processes or states where prevailing contextual conditions enable full and active participation in all aspects of everyday life.[19 20] This can include civic, social, economic, and political activities, as well as participation in decision making processes irrespective of personal characteristic differences.[19 20]

Social inclusion and occupational justice form aspects of social justice are therefore relevant to direct research and practice as we address exclusions and injustices experienced by stigmatised groups[21]. Focus on these social justice outcomes ensures that health and social well-being are addressed beyond the clinical management of the disease. Health-related quality of life that includes social inclusion and occupational justice will be a holistic construct for promoting continuum of care and health and well-being beyond viral suppression in HIV. [22 23] These concepts direct the health and social care communities to view individuals with mental illness and HIV as being part of marginalized groups at risk of being deprived of respect, rights and opportunities to achieve optimal health-related quality of life.

Social inclusion and occupational justice are potentially key concepts that can inform the promotion of human-rights based, sustainable, person and community-centred interventions that promote recovery for persons with chronic, and stigmatised conditions.[24 25] In order to aid integration, and operationalisation of occupational justice and social inclusion in practice, we need to understand how the concepts are conceptualised and applied in population groups affected by chronic and stigmatised conditions. Synthesised summaries of research evidence can inform primary research and implementation science,[26] therefore we selected a scoping review design to help advance this field.[27] This was a particularly appropriate method for this area due to the diverse disciplinary locations of the existing literature.[28 29] This scoping review aimed to explore and appraise the definitions, current utilisation, and relationships between the concepts of social inclusion and occupational justice in mental illness and HIV literature.

Methods

This scoping review followed our published study protocol[27], developed using Arksey and O'Malley's Scoping Review framework,[29] as well as guidelines for scoping review protocols in occupational therapy.[26 30] In this paper, a scoping review is taken to be a form of knowledge synthesis that addresses an exploratory research question rather than the highly focused question in a systematic review. [28 29] For reporting, we followed the Preferred Reporting Items

For peer review only - http://bmjopen.bmj.com/site/about/guidelines.xhtml

for Systematic reviews and Meta-Analyses extension for Scoping Reviews (PRISMA-ScR) Checklist. [31]

We followed an iterative process to develop and refine the research question.[29] Based on the subject area terminology, literature, and our understanding of current practice trends in managing conditions that are chronic, and stigmatised, we asked the following question:

How are occupational justice and social inclusion conceptualised, defined, and operationalised, and how are these concepts related in the highly stigmatised chronic conditions of mental illness and HIV?

The objectives of our scoping review were:

- To identify articles that define or conceptualise occupational justice and social inclusion related to mental illness and or HIV.
- To describe how these are operationalised or utilised.
- To identify and describe relationships between occupational justice and social inclusion.
- To determine potential areas for further development, integration, and application of these concepts.

Search Strategy

With the aid of a subject librarian, we identified appropriate databases using a journal indexing system. We searched twelve databases in January2019: i) PubMed; ii) Scopus; iii) Academic Search Premier; iv) the Cumulative Index to Nursing and Allied Health Literature (CINAHL); v) Africa-Wide Information; vi) Humanities International Complete; vii) Web of Science; viii) PsychInfo; ix) SocINDEX; x) Grey Literature Report; xi) Web of Science Conference Proceedings; and, xii) Open Grey. We used PubMed as the free platform for accessing articles indexed on Medline database. The selected databases captured a comprehensive sample of literature from a variety of disciplines including social work, psychiatry, nursing and occupational therapy.

The first and last authors (CN & RG) worked with the librarian, through an iterative process, to develop an inclusive list of search terms and applicable filtering methods including Boolean phrases and MESH terms for each database.[27] We developed a general search strategy with primary search terms related to the primary concepts of occupational justice and social

For peer review only - http://bmjopen.bmj.com/site/about/guidelines.xhtml

inclusion while secondary search terms encompassed the broader terms of mental health, occupational therapy, mental illness, HIV, and rehabilitation (Table 1). For the purposes of search strategy development, we restricted our search to literature published between January 1997 and January 2019, a period which has seen the emergence and rapid growth in literature on occupational justice.[32] We conducted a preliminary search on PubMed and this enabled refinement of our search strategy to maximise sensitivity and specificity. We adapted the PubMed search strategy (Appendix 1 – provided as an online supplementary appendix) accordingly for other databases.

Table 1: General Search Strategy

Key Word	Alternative words
**Occupational therapy**	Occupational rehabilitation
	**AND**
**Mental illness**	Mental health OR Mental disorder OR Psychiatric disability
	**AND**
**Occupational justice**	Occupational injustice OR Occupational marginalisation OR Occupational alienation OR Occupational imbalance OR Occupational deprivation
	**AND**
**Social inclusion**	Social exclusion OR Social isolation OR social integration
	**AND**
**HIV**	HIV OR HIV/AIDS OR HIV infection OR AIDS

Study selection

The first author (CN) reviewed the titles identified in the search for eligibility. The aim was to identify articles that i) indicated a research focus on mental illness, or HIV, or both and ii) titles that included the key terms of occupational justice, social inclusion or both. Article types included primary studies, reviews, opinion papers and other theoretical papers without primary data. Articles were not eliminated where there was uncertainty with the title until it was examined more in-depth by looking at the abstract. Two independent reviewers, the first and third authors (CN & EM), reviewed titles and abstracts of preliminarily selected articles using predetermined inclusion and exclusion criteria (Table 2), detailed in our protocol.[27] These same reviewers each further screened full-text articles to determine if they met the inclusion/exclusion criteria. At this stage, articles were included if their explicit focus was on

For peer review only - http://bmjopen.bmj.com/site/about/guidelines.xhtml

social inclusion or occupational justice in mental illness and or HIV; concepts were defined and reported some operationalisation of the two concepts. Discrepancies were resolved by consensus or by seeking adjudication from the second author (LL). The Cohen's κ statistic to determine inter-rater agreement was calculated for the title and abstract review and the full article review stage, giving more than 90% agreement between reviewers with Cohen's κ statistic of 0.78 and 0.83 respectively.

Table 2: Inclusion and exclusion criteria for acceptable articles

**Criteria for inclusion**	**Criteria for exclusion**
Minimum criteria required in the abstract:<br>• Explicit mention of mental illness, and or HIV/AIDS.<br>• Explicit mention of either occupational justice and its varieties or social inclusion and its varieties. (varieties were informed by how articles with these terms were indexed in databases)<br>• Date range (January 1997 - January 2019).<br>• English language<br>• English translation of abstract and article available	• Used animal subjects.

Data Extraction

Guided by the study objectives, a common extraction table was designed, to extract standard bibliometric information study characteristic and main findings. The first five articles were reviewed by both the first and third authors, with the remaining articles divided between the two authors. We then checked for accuracy and completeness against each other's work. Discrepancies were resolved by revisiting the article, discussing, and reaching consensus.

For peer review only - http://bmjopen.bmj.com/site/about/guidelines.xhtml

Data Synthesis

Data were synthesised descriptively to give a structured summary of the dataset and to capture the characteristics of the studies included and the definitional range of social inclusion and occupational justice. Study grouping followed the publication trends over time and study designs used. Descriptive statistics were calculated using Microsoft Excel 2016 for frequencies. We used deductive thematic analysis to organise the extracted definitions and related concepts for occupational justice and social inclusion.

Patient and public involvement:

Patient and public involvement (PPI) representatives were not directly involved in the design of this scoping review protocol. However, experiences of the first author in working with adults afflicted with HIV and mental health disorders in Zimbabwe informed the need to explore issues faced by this population beyond biomedical care. We also built our research question from insights being generated in his doctoral studies exploring occupational perspectives on social inclusion among young adults dually afflicted with substance use disorders and HIV. Social inclusion speaks to life beyond medical management which was not being given sufficient attention and hence the need to conduct a scoping review.

Results

Retained studies' characteristics

As described in Figure 1, we screened (n=3352) titles and after reviewing (n=139) full articles, (n=27) were included in this scoping review. Of the (n=27) sources included for final review, (n=23) were published between 2009-2018, with the majority of these (n=6) published in 2012 (Table 3). Most publications were by authors in the mental health field and from high income countries, with 68% of the primary studies being conducted in Europe[33-45], and 9% in Australia.[46 47] No primary studies were conducted in North America, Africa, or Asia. More than a third of the studies (n=10) utilised a qualitative research design[34-37 40-42 44 46 48 49], five (16%) utilised a quantitative research design[33 38 39 45 47], and only one study utilised a mixed methods design.[43] Six (19%) were review papers and the remainder (n=5) were opinion[50 51], lectureship[52], commentary[53] and theoretical analysis papers.[54] The two concepts were predominantly explored around mental illness (n=26) with significantly less focus on HIV (n=1). The majority (n=21) of the published research investigated social inclusion as it related to mental health conditions, and all the occupational justice papers were focussed on mental

For peer review only - http://bmjopen.bmj.com/site/about/guidelines.xhtml

illness. Only one paper was included which discussed social inclusion in relation to people living with HIV.[55] We found no published literature that explored occupational justice and social inclusion in populations with comorbid mental illness and HIV.

Insert Figure 1: **Scoping review flow diagram

For peer review only

For peer review only - http://bmjopen.bmj.com/site/about/guidelines.xhtml

Table 3: Summary of articles defining social inclusion and occupational justice in mental illness and HIV

**Author(s) Year**	**Participants**<br><br>**Setting**	**Design**	**Aim**	**Definition(s) of social inclusion and or occupational justice**	**How operationalized**	**Associated key terms used**
3a: Studies with primary data collection						
Mazzi *et al.*<br><br>2018	30 people with non-affective psychosis. In a social inclusion intervention program<br><br>Italy	Cross-sectional study	"To assess whether a social inclusion intervention is associated with better outcomes in terms of personal and social recovery, with particular reference to the areas of social functioning and activity, and subjective dimensions such as self-esteem, self-stigma and perceived quality of life" (p.1)	Social inclusion is the opportunity for an individual to participate in key functions or activities and in the economic, social and cultural life of his/her community, exercising the rights of his/her citizenship and enjoying an adequate standard of living and wellbeing.	As an outcome and intervention<br>As a human rights issue	social withdrawal, quality of life, discrimination, social recovery, social inclusion activities, social disadvantage
Saavedra *et al.*<br><br>2018	31 service users with severe mental illness. In workshops for social integration<br><br>Spain	Mixed methods study	To evaluate the impact of an artistic workshop on a group of people diagnosed/screened for with severe mental illness with focus on the impact of creative practices on well-being and social inclusion outcomes.	Social inclusion stated as a personal construct measured through perception of social isolation, social relationship and social acceptability	As an outcome to be evaluated based on personal perception<br><br>Measured using Social Inclusion Questionnaire	stigma, wellbeing, recovery, social isolation, social relationship, social acceptability

For peer review only - http://bmjopen.bmj.com/site/about/guidelines.xhtml

Table 3: Summary of articles defining social inclusion and occupational justice in mental illness and HIV						
**Author(s) Year**	**Participants**<br><br>**Setting**	**Design**	**Aim**	**Definition(s) of social inclusion and or occupational justice**	**How operationalized**	**Associated key terms used**
Berry & Greenwood<br><br>2017	51 young outpatient service users with first episode psychosis. In Early Intervention in Psychosis, Community Mental Health and Assertive Outreach services<br><br>United Kingdom	Longitud-inal study	"To investigate the direct and indirect associations between dysfunctional attitudes, self-stigma, hopefulness, social inclusion and vocational activity for young people with psychosis" (p.197).	Social inclusion comprises social activity and community belonging.	As measured by levels of social activity and community belonging using Social Relationships Scale and Social Inclusion Scale	self-stigma, hopefulness, social activity, community belonging, social network, social contact, vocational activity
Turner *et al.*<br><br>2017	71 people with psychotic-related conditions. In a research programme on schizophrenia<br><br>Ireland	Quantita-tive descriptive study	To explore "the level of social inclusion among people with psychotic-related conditions using a standardised interview" (p.195)	Social inclusion is a multi-dimensional phenomenon with a number of domains including socially valued role functioning, social support, absence of stigma, and integration in rehabilitation community and wider community	As a multi-dimensional phenomenon<br>As both objective and subjective outcome<br>Assessed using Social Inclusion Interview Schedule	supportive relationships, stigma, integration, social exclusion, social support, rehabilitation.

For peer review only - http://bmjopen.bmj.com/site/about/guidelines.xhtml

Table 3: Summary of articles defining social inclusion and occupational justice in mental illness and HIV

**Author(s) Year**	**Participants**<br><br>**Setting**	**Design**	**Aim**	**Definition(s) of social inclusion and or occupational justice**	**How operationalized**	**Associated key terms used**
Raitakari *et al.*<br><br>2016	16 mental health service users. In two mental health floating support services (FSS)<br><br>Finland	Qualitative study	To explore how community integration is understood and tackled in mental health floating support services (FSSs) and, more precisely, in service user–practitioner home visit interaction	Social inclusion is articulated in political discourse as a way to tackle social exclusion and to increase citizens' participation and activity in society	As supported integration in community life.<br><br>As participation in what marginalised individuals are usually excluded from	social exclusion, citizenship, community integration, housing, interaction, mental health, recovery
Killapsy *et al.*<br><br>2014	67 mental health service users with psychosis. Living in the boroughs of London and the inner-city<br><br>United Kingdom	Quantita-tive study	To investigate change in social inclusion after the development of a psychotic illness (clinically diagnosed) and associated factors	"Social inclusion refers to the opportunities that individuals have to participate in key areas of economic, social and cultural life" (p.148)	Focus on participation and access to services and opportunities<br><br>Five domains of social inclusion: social integration, consumption, access to services, productivity, political engagement<br>Assessed using the Social Inclusion Questionnaire User Experience	social exclusion, social disadvantage, social integration, employment, social isolation

Table 3: Summary of articles defining social inclusion and occupational justice in mental illness and HIV						
**Author(s) Year**	**Participants**<br><br>**Setting**	**Design**	**Aim**	**Definition(s) of social inclusion and or occupational justice**	**How operationalized**	**Associated key terms used**
Salles & Barros<br><br>2013a	17 mental health service users and 12 individuals from their social networks. In Psychosocial Care Centres<br><br>Brazil	Qualitative study	"To identify and analyse the conceptions expressed by the interviewees about social inclusion and mental illness" (p.37)	"Social inclusion is a process of promoting rights, access, choice and participation. For individuals with mental health problems, this also means access to the best possible forms of treatment" (p.37)	As a dynamic, multi-dimensional process.	work participation poverty
Salles & Barros<br><br>2013b	17 mental health service users and 12 individuals from their social networks. In Psychosocial Care Centres<br><br>Brazil	Qualitative study	"To identify the daily life experiences of users of a psychosocial care center related to processes of social exclusion and inclusion" (p.704).	Social inclusion is a process of the individual's choice on how to live her daily life" (p.710) and not just about experiencing full participation in society.	As a multidimensional process<br>As a human rights issue<br>As subjective experience centred on work participation	social exclusion, discrimination, prejudice, stigma, social isolation, social participation, empowerment, poverty

For peer review only - http://bmjopen.bmj.com/site/about/guidelines.xhtml

Table 3: Summary of articles defining social inclusion and occupational justice in mental illness and HIV						
**Author(s) Year**	**Participants**<br><br>**Setting**	**Design**	**Aim**	**Definition(s) of social inclusion and or occupational justice**	**How operationalized**	**Associated key terms used**
Clewes *et al.*<br><br>2013	Single Case-study with bipolar depression. In occupational therapy interventions under UK National Health Services (NHS)<br><br>United Kingdom	Case study	To illustrate how the combination of medical outpatient clinic and occupational therapy intervention together made a big difference in a person's life.	Social inclusion was taken as meaningful inclusion in the areas of life where the participant wished to be.	As personal experience.<br><br>As a degree of autonomy, ownership, responsibility, and independence.	stigma, recovery, social policy, empowerment, rights, engagement, spirituality, client leading.
Nieminen *et al.*<br><br>2012	23 mental health service users. In an intervention group follow up study<br><br>The European Union	Qualitative study	To describe how the mental health service users experienced social inclusion and employment in the European Union EMILIA project	Social inclusion is a subjective sense of belonging and active citizenship that enhances social integration	As an experience and feeling of active citizenship	empowerment, social network, finance and housing employment. social exclusion. stigma, prejudice
Fieldhouse<br><br>2012	Eight mental health service users in a two-year action research project<br><br>United Kingdom	Qualitative research	To describe an action research project that explored the recovery journeys of a group of assertive outreach service users who had progressed from being socially excluded and occupationally deprived to being participants in their local communities and to use this knowledge to inform local service development	Social inclusion is when people with mental health challenges enjoy rewarding social relations through renewed engagement in mainstream occupations resulting in fuller community participation.	As active participation in context or local community<br><br>As an outcome of occupation-based interventions.	social participation, social relationships, stigma, social exclusion, community participation, belonging, social capital

For peer review only - http://bmjopen.bmj.com/site/about/guidelines.xhtml

Table 3: Summary of articles defining social inclusion and occupational justice in mental illness and HIV						
**Author(s) Year**	**Participants**<br><br>**Setting**	**Design**	**Aim**	**Definition(s) of social inclusion and or occupational justice**	**How operationalized**	**Associated key terms used**
Stain *et al.*<br><br>2012	1825 adults with psychosis in an Australian national survey of psychosis.<br><br>Australia	Quantitative study	"To explore the impact of psychosis on an individual's social and community participation" (p. 879)	"Social inclusion refers to the participation of a person in society and is evidenced by an individual having the opportunities, resources and abilities to build and maintain relationships, engage in education and employment, and participate in community events and organisations" (p.880)	As participation in mainstream life as empowered individuals	social isolation, social anxiety, stigma, social participation, community participation
Smyth *et al.*<br><br>2011	Eight mental health service users in mental health rehabilitation services in inner-city area<br><br>United Kingdom	Qualitative study	To explore the experiences of social inclusion for mental health service users and factors associated when engaging in everyday community occupations	Social inclusion is developing fair access to opportunity in key social and economic spheres for marginalised groups.	As an issue of participation and access to services and opportunities	stigma occupational deprivation, discrimination, social networks
Ramon *et al.*<br><br>2011	27 key informants in an evaluation study of the EMILIA project<br><br>The European Union	Qualitative study	To identify how participation in the EMILIA project affects the lives of mental health service users in relation to social inclusion, employment and recovery	Social inclusion is a subjective sense of belonging and active citizenship that enhances social integration	As an experience and feeling about participation in society	employment, well-being, quality of life, social networks.

For peer review only

Table 3: Summary of articles defining social inclusion and occupational justice in mental illness and HIV

**Author(s) Year**	**Participants**<br><br>**Setting**	**Design**	**Aim**	**Definition(s) of social inclusion and or occupational justice**	**How operationalized**	**Associated key terms used**
Hamer *et al.*<br><br>2017	82 mental health service users<br><br>New Zealand & Brazil	Qualitative study	To present service users' stories of distressing exclusion that interrupted their rights to occupational justice, and marginalised them from occupation.<br>The paper also presents the practices of inclusion that service users engaged in that restored their rights and responsibilities as occupied and active citizens	Social inclusion is the extent to which people are confident about and able to exercise their rights and participate, by choice, in the ordinary activities of citizens<br><br>Occupational justice recognises the person's right to inclusive participation in everyday occupations regardless of age, ability, gender, social class, or other differences.	Social inclusion as a policy issue<br>As a dynamic concept, derived from subjective experiences<br><br>Social inclusion is enhanced through occupational justice<br><br>As inclusive participation<br>As a human right issue	occupational injustice, citizenship, stigma, discrimination
Fieldhouse<br><br>2012	Eight mental health service users in a two-year action research project<br><br>United Kingdom	Qualitative Study	To examine the impact of community participation on their recovery and social inclusion and how service users' experiences informed joint planning between mental health services and the learning community to promote social inclusion.	Social inclusion as the process of enabling citizenship through fuller community participation.<br><br>Occupational justice defined as the process of lobbying for the occupational needs of individuals and communities as part of a fair, inclusive, and empowering society; as a community reintegration issue.	As active participation in mainstream society	social participation, social relationships, stigma, social exclusion, community participation, belonging

For peer review only

Table 3: Summary of articles defining social inclusion and occupational justice in mental illness and HIV

Author(s) Year	Participants<br><br>Setting	Design	Aim	Definition(s) of social inclusion and or occupational justice	How operationalized	Associated key terms used
Farrell & Bryant<br><br>2009	Nine recruiters of volunteers who had mental illness.<br><br>United Kingdom	Qualitative study	To explore the recruiters' understanding of mental health problems, drawing on their experiences	Occupational justice is an intrinsic part of social justice, permitting equitable opportunity and the means to choose, organise and perform meaningful occupations<br><br>Social inclusion stated but not defined	As a process and an outcome<br><br>Occupational justice as an intrinsic aspect of social inclusion	volunteering, discrimination, stigmatization, social exclusion, social attitudes, prejudice, occupational deprivation, occupational marginalisation, occupational apartheid
3b: Review, commentary, lectureship and opinion papers						
Le Boutillier & Croucher<br><br>2010	Mental health service users	Opinion paper	To present an alternative to the polarised view of social inclusion	Social inclusion is a multidimensional virtuous circle aimed at improving rights of access to the social and economic world, new opportunities, recovery of social identity and meaningful life and also reduced impact of disability on everyday life.	As a multidimensional system	Social exclusion, occupational justice, occupational balance, occupational alienation, occupational deprivation

For peer review only - http://bmjopen.bmj.com/site/about/guidelines.xhtml

Table 3: Summary of articles defining social inclusion and occupational justice in mental illness and HIV

**Author(s) Year**	**Participants**<br><br>**Setting**	**Design**	**Aim**	**Definition(s) of social inclusion and or occupational justice**	**How operationalized**	**Associated key terms used**
Cobigo & Stuart<br><br>2010	Mental health service users	Review	To review recent research on approaches to improving social inclusion for people with mental disabilities	Social inclusion is when one feels accepted and recognized as an individual beyond the disability; has positive personal relationships with family, friends, and acquaintances; is actively involved in recreation, leisure, and other social activities; has appropriate living accommodations; has healthy employment;<br>and has appropriate formal (service system) and informal (family and caregiver) supports.	As an acceptance, human rights, outcome of interventions issue	stigma, discrimination, legislation, community support, disability rights, justice, human rights
Caceres *et al.*<br><br>2008	Global literature on MSM with HIV	Review	To analyse reasons for continued risk of HIV and its consequences in MSM globally	"a social inclusion perspective on HIV prevention and AIDS care implies the adoption of a broad range of strategies to understand and confront social vulnerability" (p.11)	As a perspective to addressing vulnerability	social exclusion _ describes the alienation or disenfranchisement that certain individuals or groups experience within society.<br>stigma discrimination, prejudice, human rights, poverty, migration, employment participation, sexuality
Lloyd *et al.*<br><br>2006	Mental health service users	Review	To describe a selected number of activities that promote social inclusion.	Social inclusion involves being able to re-join or participate in leisure, friendship and work communities.	Participating and accessing services and opportunities	connectedness and interdependence

For peer review only - http://bmjopen.bmj.com/site/about/guidelines.xhtml

Table 3: Summary of articles defining social inclusion and occupational justice in mental illness and HIV

Author(s) Year	Participants<br><br>Setting	Design	Aim	Definition(s) of social inclusion and or occupational justice	How operationalized	Associated key terms used
Farone<br><br>2006	Mental health service users, with a focus on schizophrenia	Review	To examine empirical evidence describing experiences with social or community integration for people with psychiatric disabilities, with a particular interest in schizophrenia	Social inclusion discussed but not defined	A link to mental and emotional well-being	community integration, community inclusion, social integration, stigma, social support, social networks
Evans & Repper<br><br>2000	Mental health service users	Review	To challenge common misconceptions surrounding employment, work, and mental health problems of mental health service users.	Social inclusion is defined as a need, aspiration, and citizenship issue among mental health service users. Social inclusion as an outcome of work participation.	A social need. An outcome of work participation	social exclusion, stigma, unemployment, poverty
Mandiberg 2012	People with psychiatric disabilities	Commentary	To describe the failure of social inclusion as a concept and present an alternative approach through community development	Social inclusion refers to full participation in the broader community for people with severe mental illnesses	As experience of participation in the broader community	work integration, community development, social enterprises
Townsend<br><br>2012	Mental health service users<br><br>Canada	Lectureship	To propose for an interdisciplinary knowledge exchange with a critical occupational perspective on the question: What lessons on boundaries and bridges to adult mental health can be drawn by connecting the capabilities and occupational frameworks of justice?	Occupational justice as the enjoyment of the 'occupational rights' of all people to engage and be socially included in their desired occupations, and thereby to contribute positively to their own well-being and the well-being of their communities.	As a human rights, capabilities and justice issue	occupational rights, occupational possibilities occupational deprivation, alienation, imbalance and marginalisation

For peer review only - http://bmjopen.bmj.com/site/about/guidelines.xhtml

Table 3: Summary of articles defining social inclusion and occupational justice in mental illness and HIV

**Author(s) Year**	**Participants**<br><br>**Setting**	**Design**	**Aim**	**Definition(s) of social inclusion and or occupational justice**	**How operationalized**	**Associated key terms used**
Harrison & Sellers<br><br>2008	Mental health service users and mental health team<br><br>United Kingdom	Opinion Paper	To explore the implications and challenges for occupational therapy roles in mental health services regarding socially inclusive practice and to discuss policy that is designed to broaden professional roles.	Occupational justice identifies inequalities in opportunities to participate in occupations.	As a human rights issue about participation in occupations<br><br>As a policy issue	social exclusion, participation, poverty, occupational deprivation
Hamer<br><br>2017	Mental health service users	Review	To discuss how social inclusion for mental health service users can be enhanced through occupational justice and the protection of their rights as citizens to have meaningful employment.	Social inclusion can be defined as the extent to which people are confident about and able to exercise their rights and participate, by choice, in the ordinary activities of citizens.<br><br>Occupational justice stated but not defined	As a human right issue	social exclusion, stigma, occupational justice, employment participation, poverty

For peer review only - http://bmjopen.bmj.com/site/about/guidelines.xhtml

For peer review only

Table 3: Summary of articles defining social inclusion and occupational justice in mental illness and HIV

**Author(s) Year**	**Participants**<br><br>**Setting**	**Design**	**Aim**	**Definition(s) of social inclusion and or occupational justice**	**How operationalized**	**Associated key terms used**
Pettican & Bryant<br><br>2007	Mental health service users	Theoretical analysis	To explore the potential of occupational justice and its related concepts. To provide the occupational therapy profession with a theoretical justification for occupational therapists adopting an occupation-focused role in community mental health teams	Social inclusion is a drive aiming to overcome discrimination and stigma faced by people with mental health problems, in order to facilitate their having equal access to mainstream employment, education and leisure opportunities.<br><br>Occupational justice is recognising and providing for the occupational needs of individuals and communities as part of a fair and empowering society	Social inclusion conceptualized as a policy drive issue<br><br>As a justice and human rights issue<br>As an occupational participation issue	occupational deprivation, occupational imbalance, occupational alienation, social justice

For peer review only

For peer review only - http://bmjopen.bmj.com/site/about/guidelines.xhtml

Social inclusion: concepts and definitions

Social inclusion was defined with high variability, with only two studies using the same definition by defining social inclusion as a subjective sense of belonging and active citizenship that enhances social integration.[40 42] Conceptualisations and definitions used ambiguous words such as 'community,' 'participation,' and 'integration' to define social inclusion.. Some studies defined social inclusion in terms of paid work and participation in community events,[38 39] others focussed on social acceptance and absence of stigma,[40 42 45 56] while still others saw it as a political discourse.[41] Stain et al.[47] tried to capture these varieties, and defined social inclusion as

> "the participation of a person in society, evidenced by an individual having the opportunities, resources and abilities to build and maintain relationships, engage in education and employment, and participate in community events and organisations" (p.880).

Notable thematic areas emerged from the analysis of the definitions, namely community participation; human rights; and social relations that enhances a sense of acceptance and belonging.

Community participation

The most prominent shared features of the definitions of social inclusion in mental illness and HIV research focussed on it being a process and an experience centred on community participation. However, the terms 'community participation' and 'community' were used in many ways without clear descriptions. These varied interpretations of community participation included reference to people with HIV or mental illness were wide ranging and included individuals with opportunities to participate in key activities in their communities like paid employment[38 39 47 57]; being integrated into the community[37 41 45]; having a sense of belonging within the community[33 40 42 51]; and exercising active citizenship.[39 55 58]

Human rights

Social inclusion was also defined and conceptualised as a human rights issue, [39 46 49 56 58] even though community participation was the penultimate indicator for social inclusion. When discussing human rights, the authors highlighted an individual's right to access resources and opportunities for personal and community growth. Social inclusion was also conceptualised as

For peer review only - http://bmjopen.bmj.com/site/about/guidelines.xhtml

the right to engage in productive occupations, with full access to work and or educational activities within the community despite one's health concerns.[58]

Social relations

Social inclusion was further conceptualised and defined as a subjective experience[33 40 42 43 45 47] where those who are socially included should experience positive relationships. While a number of authors[33 40 42 43 45 47] talked about relationships as being a key component, this was not defined or discussed in depth. Instead it was emphasised that for social inclusion to be a reality, an individual should experience positive social relationships with their significant others, family, friends and acquaintances. [33 40 42 43 45 47] Social inclusion was also conceptualised as experiencing social support and having positive support networks.[45]

Diversity in definitions reflected in measurement tools

The diversity in definitions and features was also present in the measurement tool used. Quantitative studies included one of the following social inclusion measures: i) Social Inclusion Questionnaire;[43] ii) Social Relationships Scale and Social Inclusion Scale;[33] iii) Social Inclusion Interview Schedule;[45]; and iv) Social Inclusion Questionnaire User Experience.[38] Social inclusion was also portrayed in some definitions as a subjective personal concept where it is the individual with mental illness or person living with HIV, who subjectively experiences inclusion and should have a choice on what determines their experience of such inclusion.[34 40 42 44 51] It is their perception of the quality of their relationships, their acceptability to others, and how integrated they are, which was emphasised.

In summary, social inclusion was conceptualised as processes and experiences of empowered and equitable community participation for all, in which there is respect for human rights and healthy social relations and well-being are promoted.

Conceptualising and defining occupational justice

In the last two decades, only six research papers on mental illness used the concept of occupational justice and provided a definition. [35 36 46 50 52 54] (Table 3) Five different definitions were found (Table 3). As with social inclusion, occupational justice was defined with great variability, with it being referred to as both a process and as an experience. [35 36 46 50 52 54] Two major themes emerged pointing to social justice in which occupational justice was framed as an occupational rights issue and as a matter of community participation.

For peer review only - http://bmjopen.bmj.com/site/about/guidelines.xhtml

Occupational rights

A key concept of occupational justice for individuals with mental illness was experiencing or enjoying one's occupational rights. [35 36 46 50 52 54] Occupational rights were taken to mean an individual's rights to participate in a range of meaningful and contextual occupations enabling them to flourish, fulfil their potential and experience life satisfaction in ways consistent with their contexts. Townsend (2012) defined occupational justice as the enjoyment of 'occupational rights' by all people to engage in occupations and feel socially included in their desired occupations, thereby contributing positively to their own well-being and to that of their communities.[52] Occupational justice was also highlighted as an advocacy process where individuals could lobby for the occupational rights and needs of individuals and communities as part of an equitable, inclusive, and empowering society.[36]

Community participation

The process of promoting occupational justice was viewed as related to promoting social inclusion and community participation through advancing participation.[46] In another paper community participation was emphasised, with occupational justice defined as situations when people are seen as having the opportunity to choose to participate in the community.[35] The core emphasis in the definitions was the acknowledgement of occupational justice as a means of actioning community participation and social justice.[35 46]

However, in all the definitions there was an overuse of the term 'occupation' which forms part of the term we are working to define. While 'occupation' was framed as purposeful everyday activities people engage in, it carries diverse meanings outside occupational science and occupational therapy. This rendered many of the definitions and arguments cyclical in nature.

<u>Commonalities between social inclusion and occupational justice</u>

** Insert Figure 2: **Commonalities between occupational justice and social inclusion definitions – associated terms**

Although not clearly articulated, all the papers which discussed the two concepts together seemed to infer that occupational justice was an important ingredient of social inclusion.[35 36 46 52] Notwithstanding the considerable variability in definitions and conceptualisations of the two concepts, some commonalities were identified (Figure 2). Both social inclusion and

For peer review only - http://bmjopen.bmj.com/site/about/guidelines.xhtml

occupational justice seek to promote equitable access to opportunities for engagement and to seek for fairness and justice in an individual's community participation.[35 36 46 52]

Hamer et al. provided the closest account of the relationship between the concepts positing that social inclusion was enhanced through occupational justice.[46] Here they argued that recognition of people's right to inclusive participation in everyday occupations (occupational justice) enhanced the extent to which the person became confident about and was able to exercise their rights and participate by choice in the ordinary activities as citizens (social inclusion).[46] Both concepts were centred on key thematic areas of human rights, equality, inclusivity, and community participation.

Significantly, both social inclusion and occupational justice had a social justice focus, emphasizing the right to inclusive participation in a community and individuals exercising choice of participation as part of their citizenry beyond their health conditions. Occupational justice specifies that the participation in meaningful occupations is central, while social inclusion highlights community participation. Both concepts also highlight the need to address discriminatory practices, by doing away with stigma.[45 46] Hamer and colleagues highlight that social inclusion is the process of experiencing inclusive participation in the community as a citizen, while occupational justice promotes social inclusion through participation in meaningful and valued activities.[46]

Discussion

We explored and appraised the definitions, current utilisation, and relationships between social inclusion and occupational justice in the literature on mental illness and or HIV. We identified and critically appraised 27 articles that presented a variety of definitions. Although we primarily sought out to describe the conceptualisations and utilisations of these concepts in the duality of mental illness and HIV, we found out that literature is scant and focuses on a single diagnosis. Most studies were on mental illness and conducted in high-income countries. Despite the great variability, key thematic concepts used to define social inclusion and occupational justice included community participation, human rights, and relationships. The two concepts are theoretically related through a social justice focus, putting the emphasis on treating people with HIV and those with mental illness in a more respectful and equitable manner. The highlighted thematic concepts are central in directing research and practice toward the moral imperative of addressing exclusions and injustices experienced by people living with HIV, those with mental illnesses and other stigmatised groups. [21] There is also emphasis on the

For peer review only - http://bmjopen.bmj.com/site/about/guidelines.xhtml

multidimensional nature of the concepts framed as both a process and a personal experience, also allowing a broader horizon of their application, from policy to practice. The main source of these definitions were qualitative studies using service user's experiences and experts opinions.

While definitions of social inclusion vary, our scoping review findings confirm that the definitions are still in line with the concept of poverty reduction and a focus on reducing stigma and discrimination.[44 45 55] Social inclusion emerged from European societies, in response to a welfare crisis and desire to fight disadvantage.[59] We propose that this focus on welfare and fighting disadvantage should remain and should be taken up in the mental health and HIV fields, with poverty reduction, justice, and equality as pillars of social inclusion. This would be even more effective if poverty reduction was prioritised as a specific focus of social inclusion when informing mental health and HIV policies, particularly in sub-Saharan Africa, where these problems are more prevalent and driven by poverty. [3]

The most striking observation was the lack of clarity in the definition of social inclusion, which is still evident, despite its existence in the literature for almost five decades. [59] Efforts to be all inclusive, multidimensional, person-centred and contextual can explain the variations and, thus, ambiguity in the definitions.[59 60] However, this lack of definitional clarity could hamper its universal concept utilisation, measurement, and further exploration with a common goal.[61 62] This lack of a single, universal understanding has positive and negative implications for research and application of the concept in clinical practice. Without a universally agreed upon definition, comparisons between studies and practice remains difficult.[62] This was echoed in the variety of social inclusion measures applied in the quantitative studies.[33 38 43 45] Given the variation in measures, a common understanding of what constitutes social inclusion in mental health has not been developed, despite calls for this in recent literature.[61-63] Therefore construct validity of measures is difficult to test. For the meantime, measures with a broader scope and cross-cultural validation like the Social and Community Opportunities Profile (SCOPE),[64 65] can be instrumental in developing standardized measures.

In contrast, the variations in defining the concepts reflects diversity and the importance of contexts, rather than a singular adoption of a "universal" idea. Given the diversities in experiences of mental illness and living with HIV, where different regions have their own social, economic, political, cultural, and historical realities influencing mental illness and HIV, conceptualisations of social inclusion may benefit from remaining open to multiple definitions

For peer review only - http://bmjopen.bmj.com/site/about/guidelines.xhtml

in order to reflect the realities of different regions. It is possible to have multiple, but mutual interpretations that could be understood as complementary or even contesting, in line with contextual realities.

Embracing plural definitions may be especially important in low and middle-income countries, where such research needs further growth.[59] For example different activities purported to enhance social inclusion of people with mental health challenges, like paid employment, have different meanings and impact across regions and would influence how it is conceptualised as part of social inclusion. We found a preference towards paid employment as a key determinant of community participation in social inclusion amongst people with mental health challenges.[40 47 49 57 66] This can show the dominant knowledge systems of capitalism in societies[59] where most of the studies were conducted. Other communal ways of engagement outside of a neo-liberal market structure could be explored as they may facilitate new ways of understanding inclusion in the context of HIV and mental illness. In regions where HIV and mental illness are prevalent and complex health and social care problems, we advocate for exploration of social inclusion to generate contextual knowledge, that would inform socially inclusive policies, practice and further research.

Some of the social inclusion definitions had an individual focus, for example the individual had to be actively participating at the expense of the collective found in communal societies.[38 39 43] Given that the studies were mainly from high-income regions, there remains some missing voices in informing the definitions, given the largely communal orientation found in indigenous communities in low and middle-income settings. The challenge in some of the reviewed definitions was to try to focus on the individual and the impact of HIV and or mental illness, yet the social justice agenda may better be approached from a population level with a focus on broader social determinants of health which has been the case in many countries addressing developmental and intellectual disabilities. [67] Defining social inclusion of people with mental ill health and HIV, also needs to be done from the perspective of people who are in low-income countries, experiencing poverty, unemployment, social inequality and forms of violence since people with these conditions are usually stigmatised. Discrimination and consequent social problems usually affect people as part of a collective rather than only as individuals. Hence a collective perspective that considers how groups of people are affected can strengthen the concept of social inclusion and promote its possible utility in low-resource practice contexts.

For peer review only - http://bmjopen.bmj.com/site/about/guidelines.xhtml

Though the concept of occupational justice has been present in the literature for about three decades,[32] we found limited evidence of its conceptualisation and application in mental health and no studies in HIV. [35 36 46 50 52 54] Despite the global-justice theoretical orientation of the concept, the studies found were also exclusively from high-income countries,.[35 36 46] We found key features that could guide occupational justice utilisation and further theorisation in HIV and mental health: community participation by having one's occupational rights upheld, occupational needs met, empowerment and equity in occupational participation. To some extent, the lack of diversity in regions informing the concept offers an opportunity to strengthen it by adding insights from regional contexts with potential for different realities, experiences and viewpoints, such as Africa where HIV and mental illness are prevalent and intertwined.[68] Also some authors conceptualised occupational justice as participation in occupation(s) in an equitable manner[46], they took participation as synonymous with justice, without spelling out the nature of the occupation and position of the person accessing the occupations. These have great potential in influencing how the accessed occupation impacts health, well-being and feeling of social inclusion amongst those with HIV and mental illnesses.

Despite the definitional lack of clarity, social inclusion and occupational justice are related concepts that can be used together to frame research and practice and inform policy in HIV and mental health. The commonality between the concepts is the need to promote equitable access to opportunities for community participation with fairness and equity for people with HIV and those suffering from mental illness. [35 36 46 52] The relationship between the concepts could be further developed using diverse communities to build evidence on how engagement in meaningful everyday activities underlie inclusive communities for people with mental illness and those living with HIV. This focus on occupational justice perspective presents an opportunity to routinely explore the nuances of everyday occupational participation and what that may mean for the process and experiences of social inclusion of those involved.

We therefore propose an expansion of the relationship between the conceptualisation of the two concepts, using most affected population groups, such as people with comorbid mental health disorders like substance use disorders and HIV in low-income contexts. This population group is known to have unmet broader health and social care needs hinged to the double stigma associated with substance abuse and HIV.[69] That expansion should reflect the contextual realities influencing community participation, respect of human rights, and having healthy relationships, actioned through engagement in meaningful occupations. These contextually refined concepts of social inclusion and occupational justice should then be used together to

For peer review only - http://bmjopen.bmj.com/site/about/guidelines.xhtml

inform policy, research and practice, for a just and inclusive society for those with stigmatised conditions like HIV and mental illnesses. The occupational justice and socially inclusive approach from policy through to practice, will ensure health and social well-being outcomes are addressed beyond the medical management of mental illness and or HIV. Health-related quality of life as a holistic construct for promoting continuum of care and health and well-being beyond viral suppression in HIV,[22 23] will also be made practical with a social justice lens.

Conclusion

To our knowledge, this scoping review is the first to appraise the concepts of occupational justice and social inclusion in populations afflicted by mental illness and HIV. Our findings have the potential to initiate critical conversations in the field and expand understanding and utilisation of occupational justice and social inclusion to critique and enhance global mental health. We have also presented commonalities which will give us a better theoretical foundation to inform further research, practice, and training, especially from underrepresented societies.

Author Contributions:

All authors have made substantive intellectual contributions to the conduct and write-up of this review. CN and RG conceptualised the review approach and provided general guidance to the research team. Then CN and EM were involved in systematic and independent screening and data extraction. CN provided primary input at all stages, developed all draft documents and had overall responsibility for the review. LL, RG and RH gave substantial review and critique through the review process and manuscript. All the authors reviewed and commented on the drafts of the manuscript and they all read and approved the final manuscript.

Acknowledgements:

We would like to thank Mary Shelton – UCT librarian for assistance in developing our search strategy. This article also benefited from writing soft skills and reviews by Dr Helen Jack through African Mental Health Research Initiative (AMARI).

Funding: This work was supported through the DELTAS Africa Initiative [DEL-15-01]. The DELTAS Africa Initiative is an independent funding scheme of the African Academy of Sciences (AAS)'s Alliance for Accelerating Excellence in Science in Africa (AESA) and supported by the New Partnership for Africa's Development Planning and Coordinating Agency (NEPAD Agency) with funding from the Wellcome Trust [DEL-15-01] and the UK

For peer review only - http://bmjopen.bmj.com/site/about/guidelines.xhtml

For peer review only

government. The views expressed in this publication are those of the author(s) and not necessarily those of AAS, NEPAD Agency, Wellcome Trust or the UK government.

Competing interests: None.

Data availability statement: No additional data available.

References

1. UNAIDS. UNAIDS data 2019. Geneva, 2019.
2. Pandey A, Galvani AP. The global burden of HIV and prospects for control. *The Lancet HIV* doi: 10.1016/S2352-3018(19)30230-9
3. UNAIDS. Global AIDS update 2019 — Communities at the centre — Defending rights, breaking barriers, reaching people with HIV services. Geneva, 2019.
4. Bauer-Staeb C, Jorgensen L, Lewis G, et al. Prevalence and risk factors for HIV, hepatitis B, and hepatitis C in people with severe mental illness: a total population study of Sweden. *Lancet Psychiatry* 2017;4(9):685-93. doi: 10.1016/s2215-0366(17)30253-5 [published Online First: 2017/07/09]
5. Hughes E, Bassi S, Gilbody S, et al. Prevalence of HIV, hepatitis B, and hepatitis C in people with severe mental illness: a systematic review and meta-analysis. *Lancet Psychiatry* 2016;3(1):40-48. doi: 10.1016/s2215-0366(15)00357-0 [published Online First: 2015/12/02]
6. Patel P, Rose CE, Collins PY, et al. Noncommunicable diseases among HIV-infected persons in low-income and middle-income countries: a systematic review and meta-analysis. *AIDS* 2018;32(Suppl 1):S5.
7. Bhatia R, Hartman C, Kallen MA, et al. Persons newly diagnosed with HIV infection are at high risk for depression and poor linkage to care: results from the Steps Study. *AIDS and Behavior* 2011;15(6):1161-70.
8. Mayston R, Kinyanda E, Chishinga N, et al. Mental disorder and the outcome of HIV/AIDS in low-income and middle-income countries: a systematic review. *AIDS* 2012;26:S117-S35.
9. Nyongesa MK, Mwangala PN, Mwangi P, et al. Neurocognitive and mental health outcomes and association with quality of life among adults living with HIV: a cross-sectional focus on a low-literacy population from coastal Kenya. *BMJ Open* 2018;8(9):e023914.
10. Lowther K, Selman L, Harding R, et al. Experience of persistent psychological symptoms and perceived stigma among people with HIV on antiretroviral therapy (ART): a systematic review. *Int J Nurs Stud* 2014;51(8):1171-89. doi: 10.1016/j.ijnurstu.2014.01.015 [published Online First: 2014/03/08]
11. Sin NL, DiMatteo MR. Depression treatment enhances adherence to antiretroviral therapy: a meta-analysis. *Annals of Behavioral Medicine* 2014;47(3):259-69.
12. UNAIDS. THEMATIC SEGMENT: MENTAL HEALTH AND HIV/AIDS PROMOTING HUMAN RIGHTS, AN INTEGRATED AND PERSON-CENTRED APPROACH TO IMPROVING ART ADHERENCE, WELL-BEING AND QUALITY OF LIFE. Geneva, 2018.
13. Jackson-Best F, Edwards N. Stigma and intersectionality: a systematic review of systematic reviews across HIV/AIDS, mental illness, and physical disability. *BMC Public Health* 2018;18(1):919. doi: 10.1186/s12889-018-5861-3
14. Aidala AA, Wilson MG, Shubert V, et al. Housing Status, Medical Care, and Health Outcomes Among People Living With HIV/AIDS: A Systematic Review. *American Journal of Public Health* 2016;106(1):e1-e23. doi: 10.2105/ajph.2015.302905
15. Breuer E, Myer L, Struthers H, et al. HIV/AIDS and mental health research in sub-Saharan Africa: a systematic review. *Afr J AIDS Res* 2011;10(2):101-22. doi: 10.2989/16085906.2011.593373 [published Online First: 2011/06/01]

For peer review only - http://bmjopen.bmj.com/site/about/guidelines.xhtml

16. Levi-Minzi MA, Surratt HL. HIV stigma among substance abusing people living with HIV/AIDS: implications for HIV treatment. *AIDS patient care and STDs* 2014;28(8):442-51.
17. Durocher E, Rappolt S, Gibson BE. Occupational justice: Future directions. *J Occup Sci* 2013;21(4) doi: 10.1080/14427591.2013.775693
18. Nilsson I, Townsend E. Occupational Justice—Bridging theory and practice. *Scand J Occup Ther* 2010;17(1):57-63. doi: 10.3109/11038120903287182
19. Cobigo V, Stuart H. Social inclusion and mental health. *Curr Opin Psychiatry* 2010;23(5):453-57. doi: 10.1097/YCO.0b013e32833bb305
20. Pereira RB, Whiteford GE. Understanding social inclusion as an international discourse: Implications for enabling participation. *Br J Occup Ther* 2013;76(2):112-15. doi: 10.4276/030802213X13603244419392
21. Nilsson I, Townsend E. Occupational justice—Bridging theory and practice. *Scandinavian journal of occupational therapy* 2010;17(1):57-63.
22. Lazarus JV, Safreed-Harmon K, Barton SE, et al. Beyond viral suppression of HIV – the new quality of life frontier. *BMC Medicine* 2016;14(1):94. doi: 10.1186/s12916-016-0640-4
23. Kall M, Marcellin F, Harding R, et al. Patient-reported outcomes to enhance person-centred HIV care. *The Lancet HIV* doi: 10.1016/S2352-3018(19)30345-5
24. Baumgartner JN, Burns JK. Measuring social inclusion-a key outcome in global mental health. *Int J Epidemiol* 2014;43(2):354-64. doi: 10.1093/ije/dyt224
25. Patel V, Saxena S, Frankish H, et al. Sustainable development and global mental health—a Lancet Commission. *Lancet* 2016;387(10024):1143-45. doi: 10.1016/S0140-6736(16)00208-7
26. McKinstry C, Brown T, Gustafsson L. Scoping reviews in occupational therapy: The what, why, and how to. *Australian occupational therapy journal* 2014;61(2):58-66.
27. Nhunzvi C, Langhaug L, Mavindidze E, et al. Occupational justice and social inclusion in mental illness and HIV: a scoping review protocol. *BMJ open* 2019;9(3):e024049.
28. Grant MJ, Booth A. A typology of reviews: An analysis of 14 review types and associated methodologies. *Health Info Libr J* 2009;26:91-108. doi: 10.1111/j.1471-1842.2009.00848.x
29. Arksey H, Malley LO. Scoping Studies: Towards a Methodological Framework. *Int J Soc Res Methodol* 2005;8(1):19-32. doi: 10.1080/1364557032000119616
30. Halas G, Schultz AS, Rothney J, et al. A scoping review protocol to map the research foci trends in tobacco control over the last decade. *BMJ open* 2015;5(1):e006643.
31. Tricco AC, Lillie E, Zarin W, et al. PRISMA Extension for Scoping Reviews (PRISMA-ScR): Checklist and Explanation. *Ann Intern Med* 2018;169(7):467-73. doi: 10.7326/m18-0850 [published Online First: 2018/09/05]
32. Serrata Malfitano AP, Gomes da Mota de Souza R, Esquerdo Lopes R. Occupational justice and its related concepts: An historical and thematic scoping review. *OTJR: Occupation, participation and health* 2016;36(4):167-78.
33. Berry C, Greenwood K. Direct and indirect associations between dysfunctional attitudes, self-stigma, hopefulness and social inclusion in young people experiencing psychosis. *Schizophrenia Research* 2017 doi: 10.1016/j.schres.2017.06.037
34. Clewes J, Shivamurthy S, Wrigley S. Brightening His Days: A Reflective Single Case-Study of Social Integration and Recovery. *Occupational Therapy in Mental Health* 2013;29(2):159-80.
35. Farrell C, Bryant W. Voluntary work for adults with mental health problems: An exploration of the perspectives of recruiters. *The British Journal of Occupational Therapy* 2009;72(5):188-96. doi: 10.1177/030802260907200502
36. Fieldhouse J. Community participation and recovery for mental health service users: an action research inquiry. *British Journal of Occupational Therapy* 2012;75(9):419-28.
37. Fieldhouse J. Mental health, social inclusion, and community development: lessons from Bristol. *Community Development Journal* 2012;47(4):571-87. doi: 10.1093/cdj/bss028

For peer review only - http://bmjopen.bmj.com/site/about/guidelines.xhtml

38. Killaspy H, White S, Lalvani N, et al. The impact of psychosis on social inclusion and associated factors. *International Journal of Social Psychiatry* 2014;60(2):148-54. doi: 10.1177/0020764012471918
39. Mazzi F, Baccari F, Mungai F, et al. Effectiveness of a social inclusion program in people with non-affective psychosis. *BMC Psychiatry* 2018;18(1) doi: 10.1186/s12888-018-1728-5
40. Nieminen I, Ramon S, Dawson I, et al. Experiences of social inclusion and employment of mental health service users in a european union project. *International Journal of Mental Health* 2012;41(4):3-23. doi: 10.2753/IMH0020-7411410401
41. Raitakari S, Haahtela R, Juhila K. Tackling community integration in mental health home visit integration in Finland. *Health & Social Care in the Community* 2016;24(5):E53-E62. doi: 10.1111/hsc.12246
42. Ramon S, Griffiths CA, Nieminen I, et al. Towards social inclusion through lifelong learning in mental health: Analysis of change in the lives of the EMILIA project service users. *International Journal of Social Psychiatry* 2011;57(3):211-23. doi: 10.1177/0020764009354943
43. Saavedra J, Pérez E, Crawford P, et al. Recovery and creative practices in people with severe mental illness: evaluating well-being and social inclusion. *Disability and Rehabilitation* 2018;40(8):905-11. doi: 10.1080/09638288.2017.1278797
44. Smyth G, Harries P, Dorer G. Exploring mental health service users' experiences of social inclusion in their community occupations. *British Journal of Occupational Therapy* 2011;74(7):323-31. doi: 10.4276/030802211X13099513661072
45. Turner N, Ferguson L, Hill M, et al. An exploratory study of the extent of social inclusion among people with psychosis and psychotic-related conditions. *International Journal of Social Psychiatry* 2017;63(3):195-202. doi: 10.1177/0020764017691551
46. Hamer HP, Kidd J, Clarke S, et al. Citizens un-interrupted: Practices of inclusion by mental health service users. *Journal of Occupational Science* 2017;24(1):76-87. doi: 10.1080/14427591.2016.1253497
47. Stain HJ, Galletly CA, Clark S, et al. Understanding the social costs of psychosis: The experience of adults affected by psychosis identified within the second Australian national survey of psychosis. *Australian and New Zealand Journal of Psychiatry* 2012;46(9):879-89. doi: 10.1177/0004867412449060
48. Moraes Salles M, Barros Sn. THE SOCIAL EXCLUSION/INCLUSION OF USERS OF A PSYCHOSOCIAL CARE CENTER IN EVERYDAY LIFE. *Texto & Contexto Enfermagem* 2013;22(3):704-12.
49. Salles MM, Barros S. Social representation of users of a Psychosocial Care Center and those in their social network on mental illness and social inclusion. *Saude E Sociedade* 2013;22(4):1059-71. doi: 10.1590/s0104-12902013000400009
50. Harrison D, Sellers A. Occupation for mental health and social inclusion. *British Journal of Occupational Therapy* 2008;71(5):216-19.
51. Le Boutillier C, Croucher A. Social inclusion and mental health. *British Journal of Occupational Therapy* 2010;73(3):136-39.
52. Townsend EA. Boundaries and bridges to adult mental health: Critical occupational and capabilities perspectives of justice. *Journal of Occupational Science* 2012;19(1):8-24.
53. Mandiberg JM. Commentary: the failure of social inclusion: an alternative approach through community development. *Psychiatric Services* 2012;63(5):458-60.
54. Pettican A, Bryant W. Sustaining a focus on occupation in community mental health practice. *British Journal of Occupational Therapy* 2007;70(4):140-46.
55. Cáceres CF, Aggleton P, Galea JT. Sexual diversity, social inclusion and HIV/AIDS

AIDS 2008;22(Suppl2):S45-S55.
56. Cobigo V, Stuart H. Social inclusion and mental health. *Current Opinion in Psychiatry* 2010;23(5):453-57. doi: 10.1097/YCO.0b013e32833bb305
57. Evans J, Repper J. Employment, social inclusion and mental health. *Journal of Psychiatric and Mental Health Nursing* 2000;7(1):15-24. doi: 10.1046/j.1365-2850.2000.00260.x

For peer review only - http://bmjopen.bmj.com/site/about/guidelines.xhtml

58. Hamer H. Returning citizens: Social inclusion and occupational justice for mental health service users. *Cadernos Brasileiros de Saúde Mental/Brazilian Journal of Mental Health* 2017;9(21):89-113.
59. Rawal N. Social inclusion and exclusion: A review. *Dhaulagiri Journal of Sociology and Anthropology* 2008;2:161-80.
60. Silver H. The contexts of social inclusion. 2015.
61. Baumgartner JN, Burns JK. Measuring social inclusion—a key outcome in global mental health. *International journal of epidemiology* 2013;43(2):354-64.
62. O'Donnell P, O'Donovan D, Elmusharaf K. Measuring social exclusion in healthcare settings: a scoping review. *International journal for equity in health* 2018;17(1):15.
63. Huxley P, Thornicroft G. Social inclusion, social quality and mental illness. *The British Journal of Psychiatry* 2003;182(4):289-90.
64. Huxley PJ, Chan K, Chiu M, et al. The social and community opportunities profile social inclusion measure: Structural equivalence and differential item functioning in community mental health residents in Hong Kong and the United Kingdom. *International Journal of Social Psychiatry* 2016;62(2):133-40.
65. Chan K, Evans S, Ng YL, et al. A Concept Mapping Study on Social Inclusion in Hong Kong. *Social Indicators Research* 2014;119(1):121-37. doi: 10.1007/s11205-013-0498-1
66. Lloyd C, Tse S, Deane FP. Community participation and social inclusion: How practitioners can make a difference. *AeJAMH (Australian e-Journal for the Advancement of Mental Health)* 2006;5(3):1-10. doi: 10.5172/jamh.5.3.185
67. Martin L, Cobigo V. Definitions matter in understanding social inclusion. *Journal of Policy and Practice in Intellectual Disabilities* 2011;8(4):276-82.
68. Brandt R. The mental health of people living with HIV/AIDS in Africa: a systematic review. *African Journal of AIDS Research* 2009;8(2):123-33.
69. Duffy M, Sharer M, Cornman H, et al. Integrating Mental Health and HIV Services in Zimbabwean Communities: A Nurse and Community-led Approach to Reach the Most Vulnerable. *Journal of the Association of Nurses in AIDS Care* 2015:1-13. doi: 10.1016/j.jana.2015.09.003

For peer review only

For peer review only - http://bmjopen.bmj.com/site/about/guidelines.xhtml

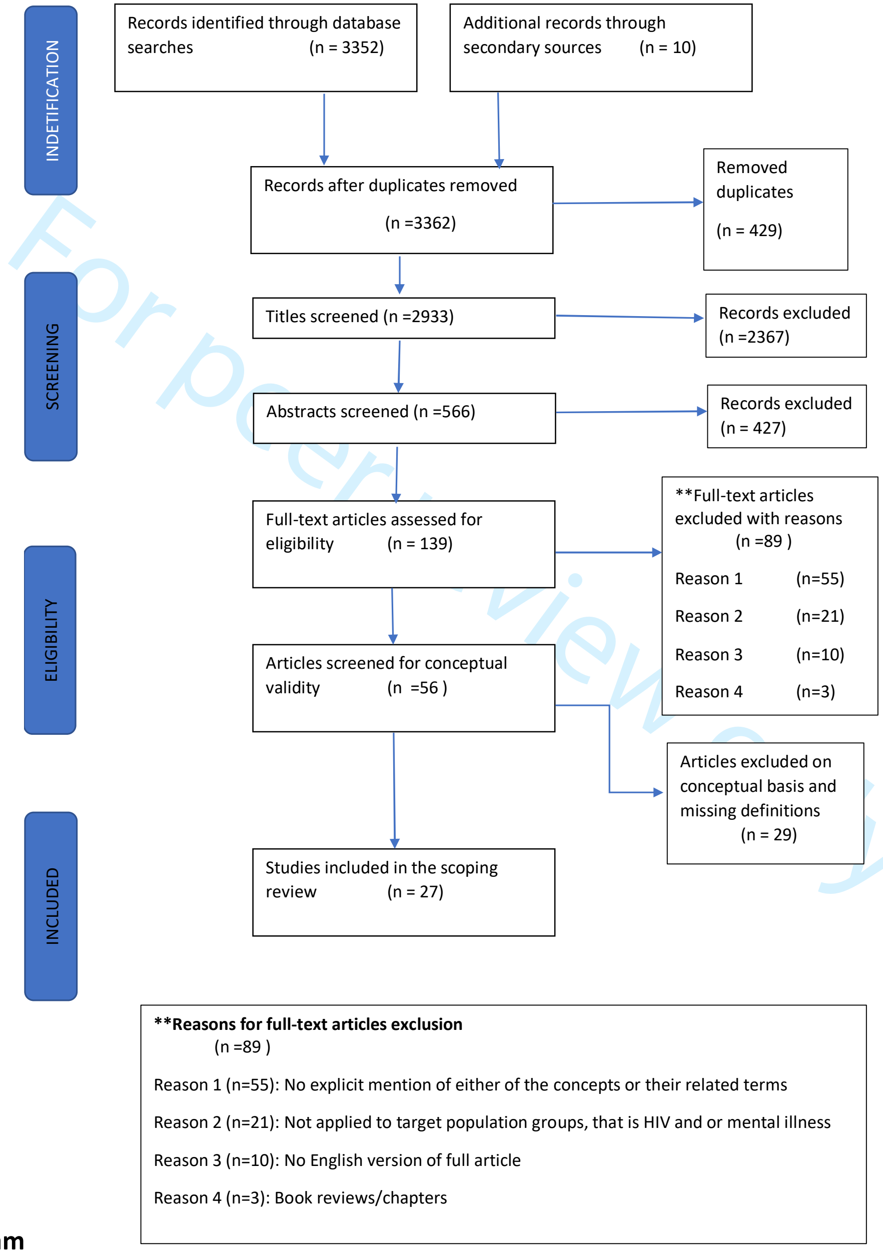

Figure 1: Scoping review flow diagram

For peer review only - http://bmjopen.bmj.com/site/about/guidelines.xhtml

Figure 2: Commonalities between occupational justice and social inclusion definitions – associated terms

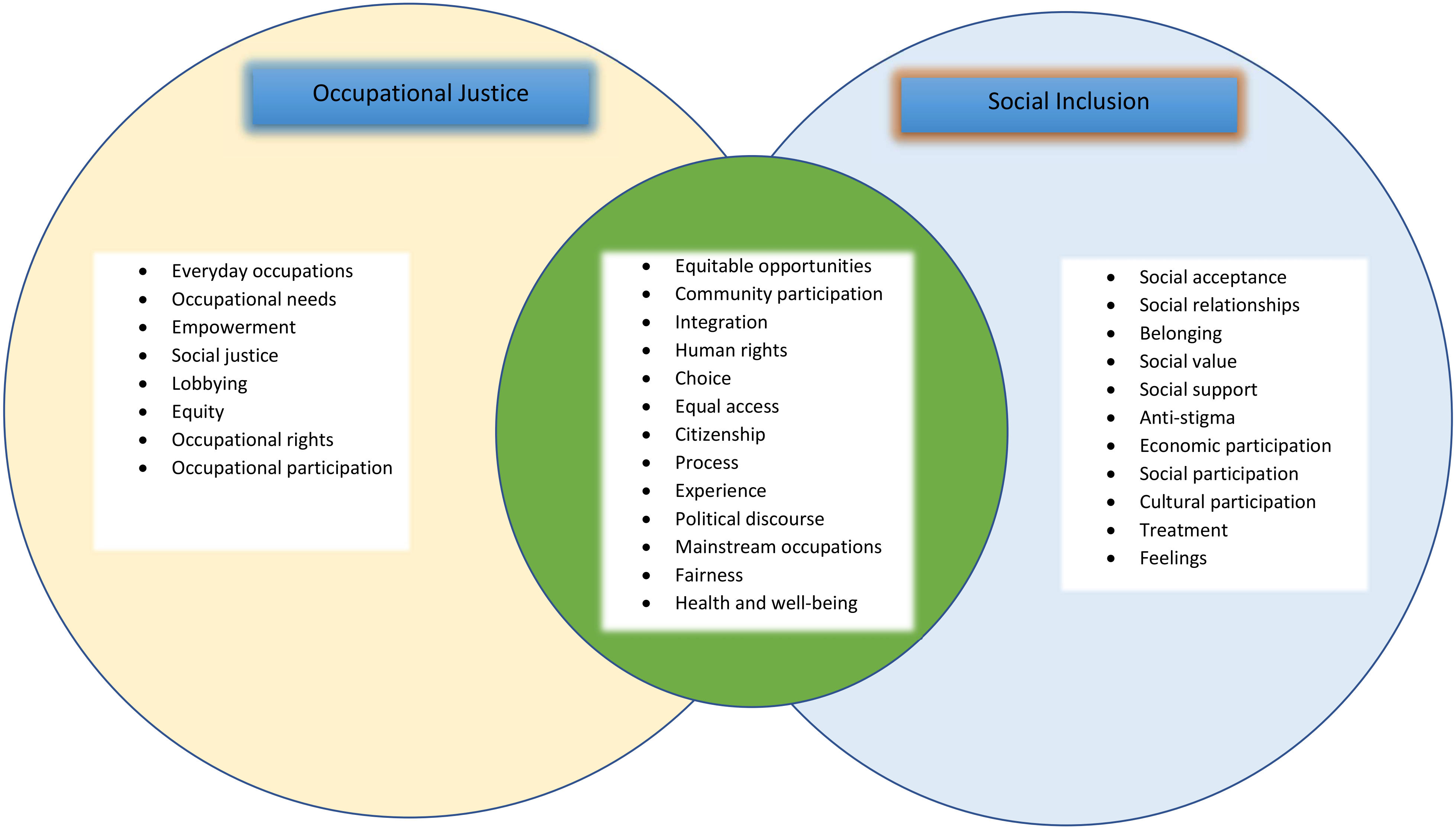

Appendix 1. PubMed Search Strategy

PubMed

Set 1

1. Social Justice [MeSH]

2. Social Isolation [MeSH]

3. Social Marginalization [MeSH]

4. Social Participation [MeSH]

5. Rehabilitation, Vocational [MeSH]

6. Injustice OR justice OR social inclusion OR social exclusion OR social isolation OR social separation OR social barriers OR social distance OR social acceptance OR social rejection OR social participation OR deprivation OR marginalization OR alienation

7. 1 OR 2 OR 3 OR 4 OR 5 OR 6 *(represents Social inclusion set)*

Set 2

1. Social stigma [MeSH]

2. Prejudice [MeSH]

3. Stigma OR prejudice OR stigmatise OR stigmatisation OR stigmatize OR stigmatization OR discrimination

4. 1 OR 2 OR 3 *(represents Stigma set)*

Set 3

1. Occupational justice OR Occupational injustice OR Occupational deprivation OR Occupational alienation OR Occupational marginalisation OR Occupational imbalance OR Occupational OR occupation OR occupations OR activities OR work OR employment OR unemployment OR engagement (*this set is used to narrow search to occupation as defined by OT not PubMed's definition)*

Set 4

1. Mental Disorders [MeSH] *(this heading includes substance-related disorders)*

2. Mentally Ill Persons [MeSH]

For peer review only

3. Mental disorders OR mental illness OR mentally ill OR Psychiatric disorder OR psychiatric illness OR psychological disorder OR Developmental Disability OR Intellectual Development Disorder OR Intellectual disability OR Mental retardation OR Mental deficiency
4. 1 OR 2 OR 3 *(represents Mental Disorders set)*

Set 5

1. HIV [MeSH]
2. HIV Infections [MeSH]
3. Acquired Immunodeficiency Syndrome (MeSH)
4. HIV OR human immune deficiency virus OR AIDS OR acquired immunodeficiency syndrome OR acquired immune deficiency syndrome OR HIV/AIDS
5. 1 OR 2 OR 3 OR 4

Now combine Sets, 1 AND 2 AND 3 AND 4 or Sets 1 AND 2 AND 3 AND 5
Limit to last 20 years

English

Date: 31/01/19

For peer review only

Preferred Reporting Items for Systematic reviews and Meta-Analyses extension for Scoping Reviews (PRISMA-ScR) Checklist

SECTION	ITEM	PRISMA-ScR CHECKLIST ITEM	REPORTED ON PAGE #
**TITLE**			
Title	1	Identify the report as a scoping review.	Page 1
**ABSTRACT**			
Structured summary	2	Provide a structured summary that includes (as applicable): background, objectives, eligibility criteria, sources of evidence, charting methods, results, and conclusions that relate to the review questions and objectives.	Page 2
**INTRODUCTION**			
Rationale	3	Describe the rationale for the review in the context of what is already known. Explain why the review questions/objectives lend themselves to a scoping review approach.	Page 6
Objectives	4	Provide an explicit statement of the questions and objectives being addressed with reference to their key elements (e.g., population or participants, concepts, and context) or other relevant key elements used to conceptualize the review questions and/or objectives.	Page 6
**METHODS**			
Protocol and registration	5	Indicate whether a review protocol exists; state if and where it can be accessed (e.g., a Web address); and if available, provide registration information, including the registration number.	Yes ref 27
Eligibility criteria	6	Specify characteristics of the sources of evidence used as eligibility criteria (e.g., years considered, language, and publication status), and provide a rationale.	Page 7
Information sources*	7	Describe all information sources in the search (e.g., databases with dates of coverage and contact with authors to identify additional sources), as well as the date the most recent search was executed.	Page 7
Search	8	Present the full electronic search strategy for at least 1 database, including any limits used, such that it could be repeated.	Page 7. Appendix 1
Selection of sources of evidence†	9	State the process for selecting sources of evidence (i.e., screening and eligibility) included in the scoping review.	Page 7
Data charting process‡	10	Describe the methods of charting data from the included sources of evidence (e.g., calibrated forms or forms that have been tested by the team before their use, and whether data charting was done independently or in duplicate) and any processes for obtaining and confirming data from investigators.	Page 8
Data items	11	List and define all variables for which data were sought and any assumptions and simplifications made.	Page 11. Table 3
Critical appraisal of individual sources of evidence§	12	If done, provide a rationale for conducting a critical appraisal of included sources of evidence; describe the methods used and how this information was used in any data synthesis (if appropriate).	Click here to enter text.

For peer review only

St. Michael's
Inspired Care.
Inspiring Science.

For peer review only - http://bmjopen.bmj.com/site/about/guidelines.xhtml

SECTION	ITEM	PRISMA-ScR CHECKLIST ITEM	REPORTED ON PAGE #
Synthesis of results	13	Describe the methods of handling and summarizing the data that were charted.	Page 8
**RESULTS**			
Selection of sources of evidence	14	Give numbers of sources of evidence screened, assessed for eligibility, and included in the review, with reasons for exclusions at each stage, ideally using a flow diagram.	Page 9. Figure 1
Characteristics of sources of evidence	15	For each source of evidence, present characteristics for which data were charted and provide the citations.	Page 11-22. Table 3
Critical appraisal within sources of evidence	16	If done, present data on critical appraisal of included sources of evidence (see item 12).	Click here to enter text.
Results of individual sources of evidence	17	For each included source of evidence, present the relevant data that were charted that relate to the review questions and objectives.	Table 3
Synthesis of results	18	Summarize and/or present the charting results as they relate to the review questions and objectives.	Page 11-22
**DISCUSSION**			
Summary of evidence	19	Summarize the main results (including an overview of concepts, themes, and types of evidence available), link to the review questions and objectives, and consider the relevance to key groups.	Page 26-29
Limitations	20	Discuss the limitations of the scoping review process.	Page 2
Conclusions	21	Provide a general interpretation of the results with respect to the review questions and objectives, as well as potential implications and/or next steps.	Page 30
**FUNDING**			
Funding	22	Describe sources of funding for the included sources of evidence, as well as sources of funding for the scoping review. Describe the role of the funders of the scoping review.	Page 31

JBI = Joanna Briggs Institute; PRISMA-ScR = Preferred Reporting Items for Systematic reviews and Meta-Analyses extension for Scoping Reviews.
* Where *sources of evidence* (see second footnote) are compiled from, such as bibliographic databases, social media platforms, and Web sites.
† A more inclusive/heterogeneous term used to account for the different types of evidence or data sources (e.g., quantitative and/or qualitative research, expert opinion, and policy documents) that may be eligible in a scoping review as opposed to only studies. This is not to be confused with *information sources* (see first footnote).
‡ The frameworks by Arksey and O'Malley (6) and Levac and colleagues (7) and the JBI guidance (4, 5) refer to the process of data extraction in a scoping review as data charting.
§ The process of systematically examining research evidence to assess its validity, results, and relevance before using it to inform a decision. This term is used for items 12 and 19 instead of "risk of bias" (which is more applicable to systematic reviews of interventions) to include and acknowledge the various sources of evidence that may be used in a scoping review (e.g., quantitative and/or qualitative research, expert opinion, and policy document).

From: Tricco AC, Lillie E, Zarin W, O'Brien KK, Colquhoun H, Levac D, et al. PRISMA Extension for Scoping Reviews (PRISMAScR): Checklist and Explanation. Ann Intern Med. 2018;169:467–473. doi: 10.7326/M18-0850.
